# Rules for Dibenzocyclooctadiene Conformational Dynamics

**DOI:** 10.1021/acs.jnatprod.5c01348

**Published:** 2026-01-31

**Authors:** Luke P. Robertson, Wen Xu, Louisa Brieskorn, Iro Chaitoglou, Jing Guo, Runyue Huang, Per-Johan Jakobsson, Gennaro Pescitelli, Ulf Göransson

**Affiliations:** † Pharmacognosy, Department of Pharmaceutical Biosciences, Uppsala University, Uppsala 75123, Sweden; ‡ State Key Laboratory of Traditional Chinese Medicine Syndrome, The Second Clinical College, 47879 Guangzhou University of Chinese Medicine, Guangzhou 510006, China; § Division of Rheumatology, Department of Medicine, Solna, Karolinska Institutet, and Karolinska University Hospital, Solna, and Center for Molecular Medicine, 59562 Karolinska University Hospital, Stockholm 17176, Sweden; ∥ Section of Rheumatology and Immunology Research, The Second Affiliated Hospital of Guangzhou University of Chinese Medicine (Guangdong Provincial Hospital of Chinese Medicine), Guangzhou 510006, China; ⊥ Dipartimento di Chimica e Chimica Industriale, Università di Pisa, Via Giuseppe Moruzzi, 13, Pisa 56124, Italy

## Abstract

The conformational dynamics of flexible compounds can meaningfully influence their NMR spectra and biological activities, yet these effects are easily overlooked. The dibenzocyclooctadiene lignans provide a clear example of this. Although >350 naturally occurring dibenzocyclooctadienes have been published, discrete spectral features have gone unnoticed, and misconceptions about their conformational dynamics pervade the literature. Our attention was drawn to this after observing ^13^C NMR signal broadening at several resonances in a series of new dibenzocyclooctadienes isolated from *Kadsura heteroclita*, (kadheterins I-K, **1**-**3**). To understand this, we reviewed the ^13^C NMR spectra of 71 published dibenzocyclooctadienes and found that >70% displayed the same broadening, yet the underlying cause had not been clearly rationalized. Systematic analysis revealed that this broadening is associated with key benzylic substituents at C-6. Computational and VT-NMR analyses revealed that these introduce destabilizing steric interactions in the twist-boat chair (TBC) conformation, promoting exchange with the less stable twist-boat (TB). In contrast, certain substituents (e.g., at C-7/C-8) were found to stabilize the TBC. Therefore, many dibenzocyclooctadienes previously described as adopting discrete TB/TBC conformers are interconverting mixtures. We have condensed our observations into a set of rules that predict how common substituents affect ring dynamics in this class of compounds.

## Graphical abstract

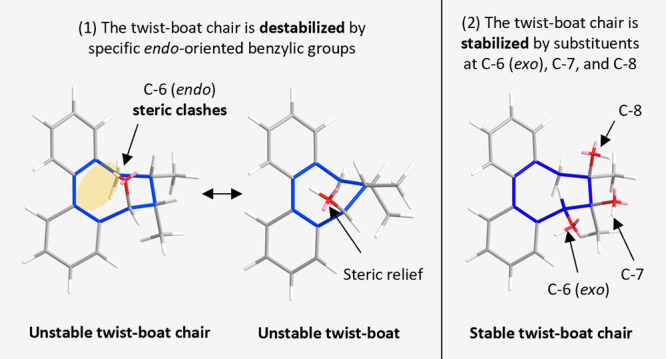

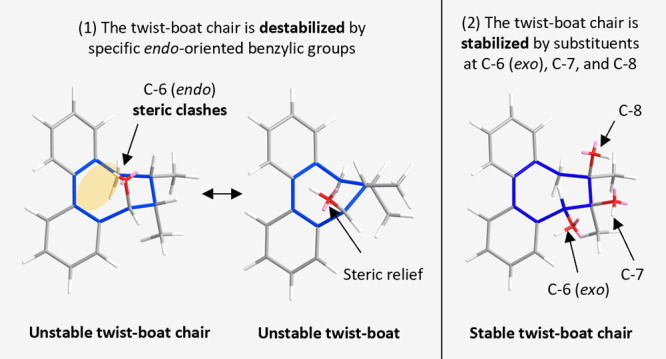

Conformational isomerism refers to compounds with the same atomic connectivity that adopt different spatial arrangements due to rotations around single bonds. Interconversion between conformers is often rapid, and whether individual species can be observed depends on a combination of (a) the time scale of measurement, (b) the relative energetic favorability of each, and (c) the energy barriers for interconversion. NMR is the most common method for studying conformational isomerism in solution: when interconversion is fast relative to the NMR time scale, signals from multiple conformers coalesce into a single averaged resonance. At intermediate rates, signals broaden and may appear partially merged, and when interconversion is slow, distinct resonances can be observed for each conformer (assuming interconversion occurs at all).^1,2^ While modern compound characterization routinely establishes atomic connectivity and absolute stereochemistry, the conformational behavior of flexible molecules can be more subtle and complex. Because the solution conformation of a compound is often vital to its biological function,^3,4^ understanding and predicting molecular conformational dynamics is crucial for both structure elucidation and drug design.

The dibenzocyclooctadiene lignans serve as a perfect model system for illustrating several aspects of conformational isomerism. To date, over 350 dibenzocyclooctadiene natural products have been isolated from the Schisandraceae,^5^ and a substructure search of the parent 7,8-dimethyldibenzocyclooctadiene backbone on SciFinder yields over 3200 results. They have drawn significant attention regarding their structures, synthesis,^6,7^ NMR spectral characteristics,^8^ and biological activities.^5,9^ The clinical relevance of this class is highlighted by the semisynthetic derivative bicyclol, which is approved by the Chinese FDA for the treatment of liver disorders and hepatitis.^10^ Structurally, the dibenzocyclooctadienes have two key features: atropisomerism about the biaryl bond, and conformational flexibility of the cyclooctadiene. Since the carbons *ortho-* to the biaryl bond are almost always substituted, the energy barrier for biaryl rotation is high (≥35 kcal/mol), and atropisomers are generally separable.^11^ The vast majority of dibenzocyclooctadienes adopt the (a*P*) axial configuration, corresponding to the axial chirality referred to as (a*S*) for this scaffold.^5^ While establishing the biaryl configuration is generally straightforward with electronic circular dichroism (ECD), elucidating the conformation of the cyclooctadiene ring is more challenging.

The cyclooctadiene ring can adopt one of two forms: the twist-boat chair (TBC) or the twist-boat (TB), corresponding to an axial/equatorial flip of C-17/C-18 (Figure 1). Of the two, the TBC is more commonly reported and energetically favorable,^11^ although some dibenzocyclooctadienes with TB conformations have been described.^6^ Distinguishing TBC and TB conformers is usually done with NMR, although some reports support their proposals with X-ray structures.^11–13^ However, while the absolute configurations of the chiral centers of the cyclooctadiene are reported for new compounds, their TB/TBC conformations are often not explicitly addressed. Seminal early work by Gottlieb et al. on dibenzocyclooctadiene conformational dynamics demonstrated that benzylic ketones can stabilize the TB form through conjugation with adjacent aromatic rings, provided the geometry allows for the required coplanarity.^11^ Benzylic ketones are, however, rare in naturally occurring dibenzocyclooctadienes. Beyond this, there are few clear descriptions in the literature about which substituents cause a preference for TB or TBC conformations – or which lead to structures with no strong predilection for either.

**Figure 1:**
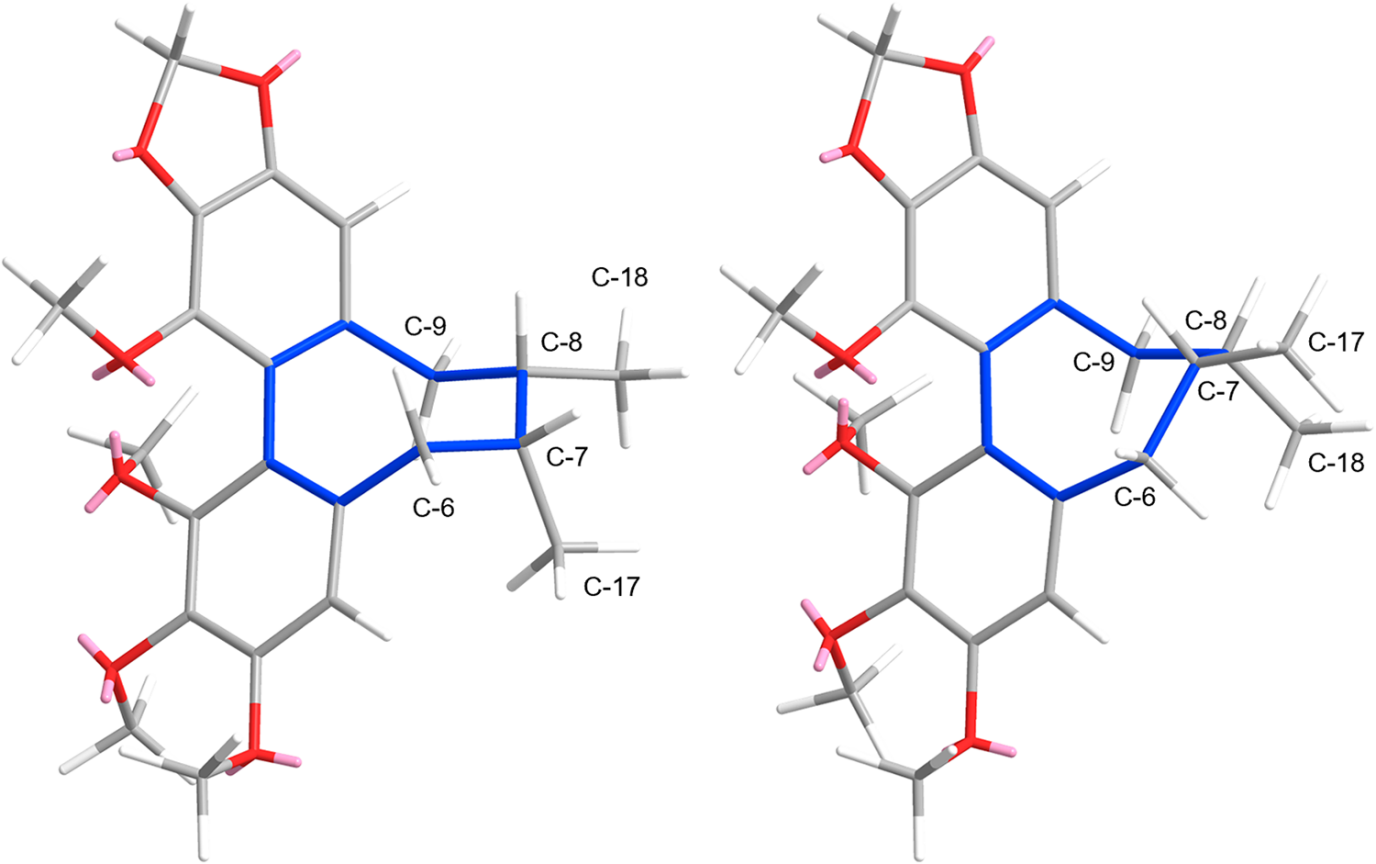
Example (a*S*)-configured dibenzocyclooctadienes in twist-boat chair (TBC, left) and twist-boat (TB, right) conformations, with the cyclooctadiene marked in blue.

Several *Kadsura* and *Schisandra* species are of importance in traditional Chinese medicine, where they are used to treat rheumatoid arthritis and other inflammatory disorders.^14^ As part of our work investigating the antirheumatic effects of traditional Chinese medicinal plant formulations,^15^ we found that *Kadsura heteroclita* (Schisandraceae) showed anti-inflammatory activities in our cell reporter assays. A subsequent chemical investigation yielded seven dibenzocyclooctadienes, of which three were new (**1**-**3**) and four known (**4**-**7**). We observed that four of these (**1**-**3**, **5**) showed broadened ^13^C resonances associated with specific cyclooctadiene carbons (C-7/C-8/C-9/C-17/C-18), prompting further investigation. We found that this broadening, although very frequently encountered in these compounds, has not been clearly rationalized in the literature. Through the systematic reanalysis of published NMR spectra combined with variable temperature NMR (VT-NMR) and density functional theory (DFT) studies, we demonstrate that this broadening arises from the presence of key benzylic substituents that cause steric clashes with proximal aryl rings and promote interconversion between TBC and TB conformers. α-Oriented substituents at C-6, and groups at C-7/C-8 were found to mitigate this effect and stabilize the TBC. Our findings have been distilled into the first set of rules that can serve as the basis for conformational and structural analysis of this distinct class of compounds.

## Results and Discussion

Kadheterin I (**1**) was isolated as a yellow amorphous solid from the stem bark of *K. heteroclita*. Comprehensive spectroscopic analysis (Supporting Information, S1) was used to identify **1** as a dibenzocyclooctadiene with an *S*-configured biphenyl core, bearing 2-methylbutyryl and acetyl groups at C-6 and C-9, respectively, and an absolute configuration of (a*S*,6*R*,7*S*,8*R*,9*R*)-**1** (Figure 2). Although this structure could be established with confidence, we observed several unexplained NMR data anomalies in **1**. Neither C-17 nor C-18 show clear crosspeaks in the HSQC spectrum (with C-17 also obscured by signal overlap with C-5′), nor could they be observed in its ^13^C NMR spectrum. The peaks associated with carbons at C-7/C-8/C-9 were also broadened or absent, however these could be observed by increasing the temperature to 323 K (Figure 3). The ^13^C and HSQC spectra of **2** and **3** also show the same behavior, wherein resonances at C-7/C-8/C-9/C-17/C-18 are either broad or absent at 298 K. These observations suggested the possibility of TB/TBC conformational equilibrium in **1**-**3** (new compounds reported herein, named kadheterins I, J, and K), and thus we sought evidence of this using NOESY data. While TB/TBC conformations can be distinguished by NOE correlations from H_3_-17 and H_3_-18 to other cyclooctadiene protons, this was hindered by signal overlap in several of the compounds: H_3_-17 overlaps with H_3_-5′ in **1** (δ_H_ 0.91/0.92), and H_3_-17/H_3_-18 overlap in **2** (δ_H_ 0.94/0.96). Fortunately, H_3_-17/H_3_-18 were well resolved in **3** (δ_H_ 0.96/1.02), facilitating NOESY analysis. The NOESY spectrum of **3** showed a strong cross-peak between H-4/H_3_-17, which would be observable in both TB and TBC conformations (interproton distances of 2.5 Å in TBC vs 4.3 Å in TB) (Figure 4). Weak cross-peaks between H-4/H_3_-18 (5.5 Å in TBC vs 2.9 Å in TB), H-6/H_3_-18 (5.0 Å in TBC vs 2.7 Å in TB), and H-7/H-11 (5.0 Å in TBC vs 3.2 Å in TB) indicated some TB population. However, a strong correlation between H-4/H_3_-17 (2.5 Å in TBC vs 4.3 Å in TB) indicated a large TBC population. Taken together, the NOESY and ^13^C NMR data of **1**-**3** indicated that TB/TBC conformational exchange was occurring. To assess whether TB/TBC exchange and/or ^13^C NMR signal broadening are commonly reported features of this compound class, we surveyed the literature on recently published dibenzocyclooctadienes.

**Figure 2:**
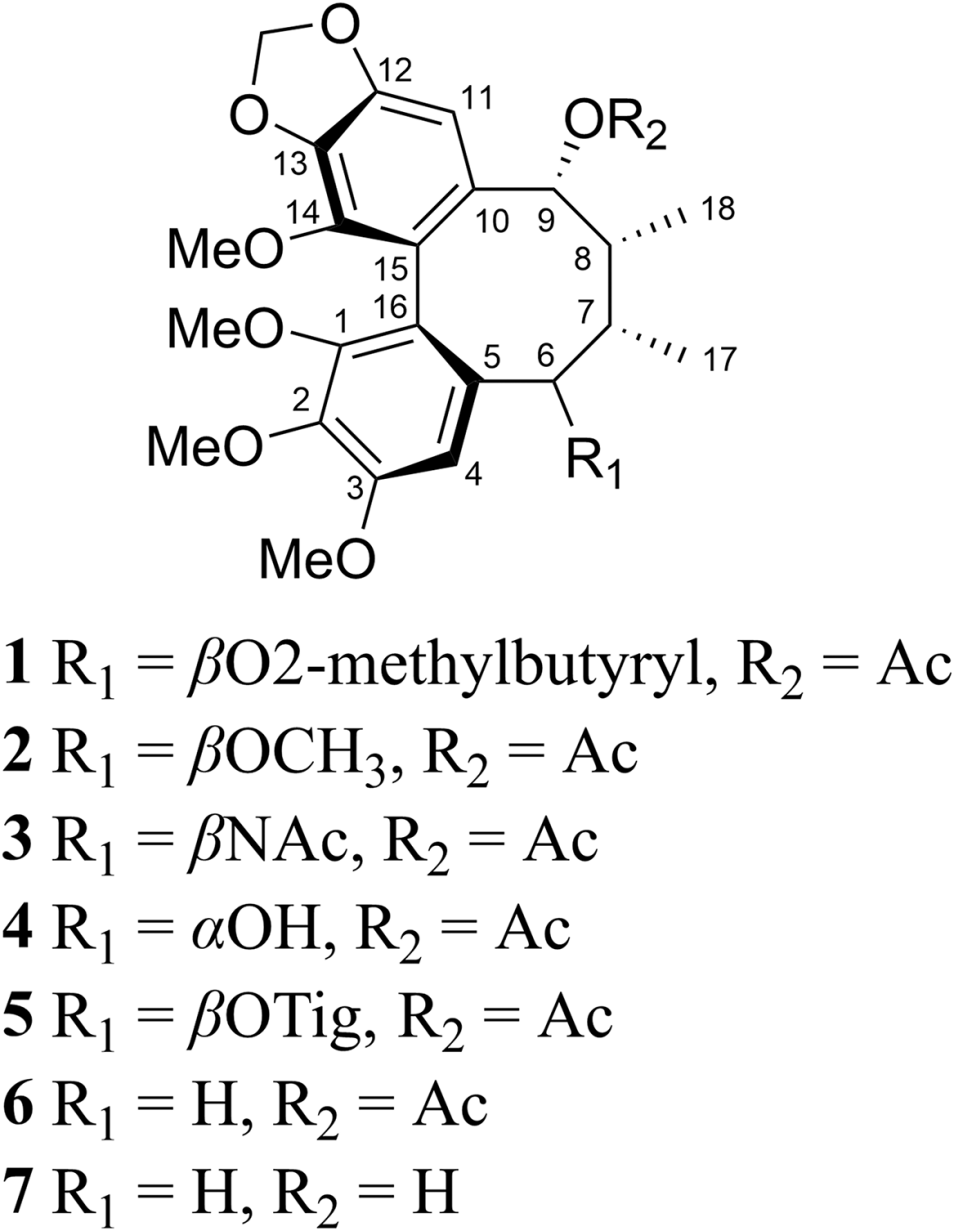
Dibenzocyclooctadienes isolated from *Kadsura heteroclita* in the current study. Compounds **1–3** are new, while **4**-**7** are previously reported.

**Figure 3:**
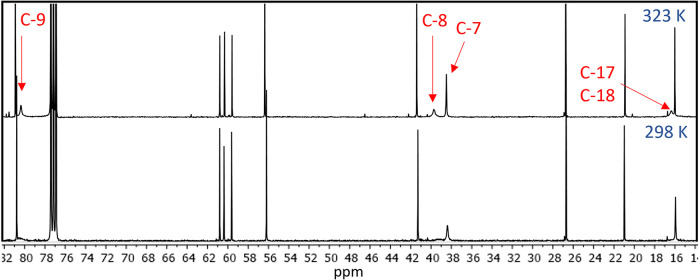
^13^C NMR spectra of **1** acquired at 323 K (top) and 298 K (bottom) in CDCl_3_. The peaks associated with C-7/C-8/C-9/C-17/C-18 are broad or absent 298 K, but these can be observed by increasing the temperature to 323 K.

**Figure 4:**
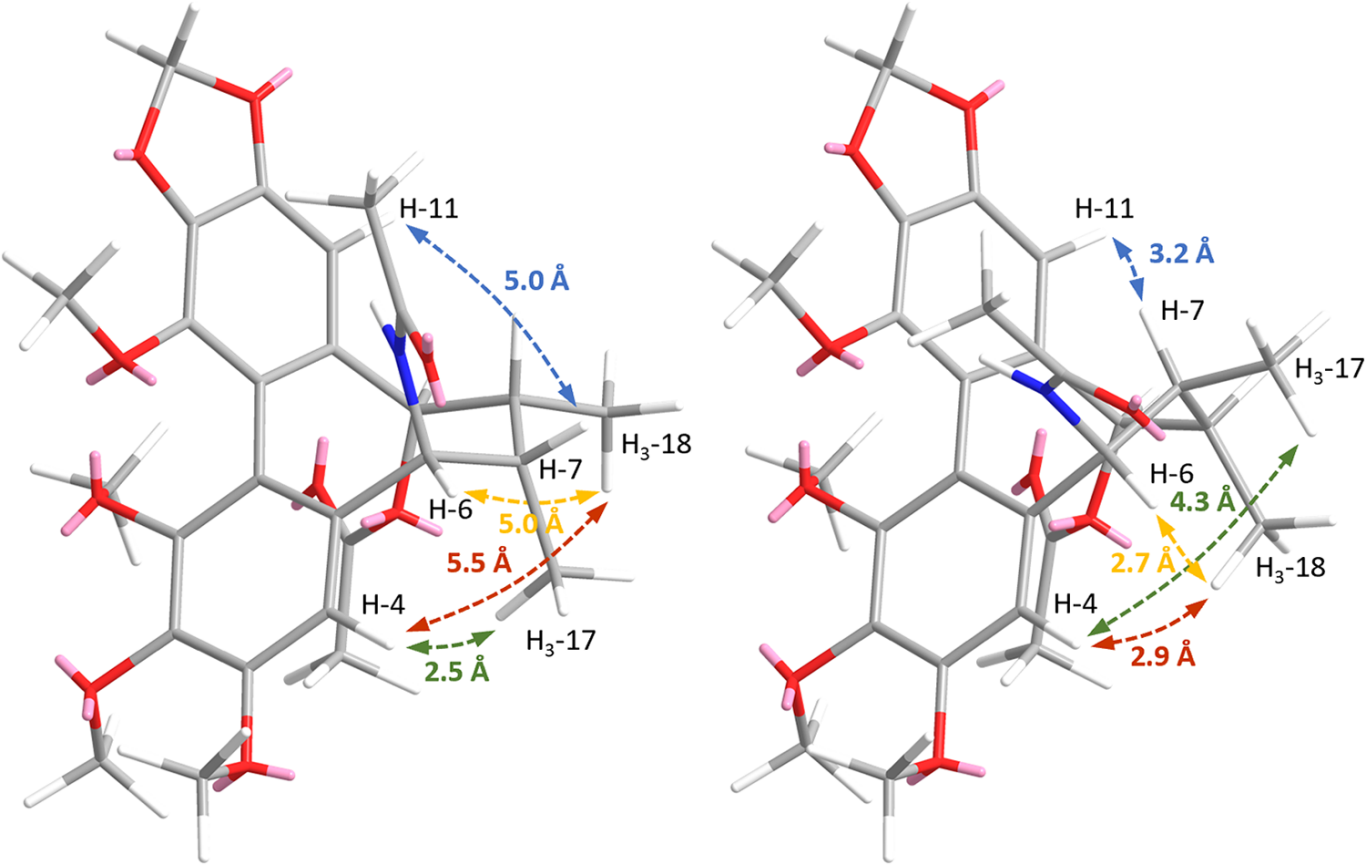
Key interproton distances in TBC (left) and TB (right) conformers of **3**. Distances between H-4/H_3_-17 are marked in green, H-4/H_3_-18 in red, H-6/H_3_18 in yellow, and H-7/H-11 in blue.

Seven recently published sets of dibenzocyclooctadienes were analyzed, totalling 71 compounds. These included polysperlignans A-K (from *Kadsura polysperma*),^12^ kadheterins A-H (*K. heteroclita*),^13^ heilaohuguosus A-M (*K. coccinea*),^16^ ananolignans A-N,^17^ ananonins A-N (both *K. ananosma*),^18^ marlignans M-S (*Schisandra wilsoniana*),^19^ and kadsuindutains A-E (*K. induta*).^20^ Recent papers were chosen from standard natural product journals (*Journal of Natural Products, Tetrahedron, Fitoterapia, Phytochemistry*, and *RSC Advances)* wherein authors had provided NMR supplementary data and the ^13^C spectra could be manually examined. Of 71 compounds, 50 showed similar peak broadening of C-7/C-8/C-9/C-17/C-18 resonances, with many of them not visible or picked in the ^13^C NMR spectra (Figure 5). The ^13^C spectrum of marlignan R, in which C-6 is substituted by a β-oriented methoxyl group, was missing from the supplementary data, although analysis of the assigned chemical shifts indicates that the same phenomenon occurred.^19^ Surprisingly, in none of these papers were the broadened signals commented upon by the authors, and many assigned carbon resonances were absent from the supplementary ^13^C spectra. A 2021 review on the NMR characteristics of dibenzocyclooctadienes also fails to mention this phenomenon,^8^ and no other explanations could be found in the literature. After comparing these data with our own observations, a pattern emerged wherein it was established that compounds with a β-oriented substituent on C-6 show broadened resonances for C-7/C-8/C-9/C-17/C-18. This effect was not seen in two key cases:

(1) If the substituent at C-6 is α-oriented (ananolignans C and D, heilaohuguosus I and M)
(2) If C-7 is substituted by another or different group, besides CH_3_-17 (e.g., a hydroxyl, see kadheterins C–H; a methylidene, see kadheterin A; or an oxirane, see kadheterin B) (Figure 5). Examples of the hydroxyl group at C-7 being both α- and β-oriented can be observed (e.g., kadheterins C–H).

**Figure 5:**
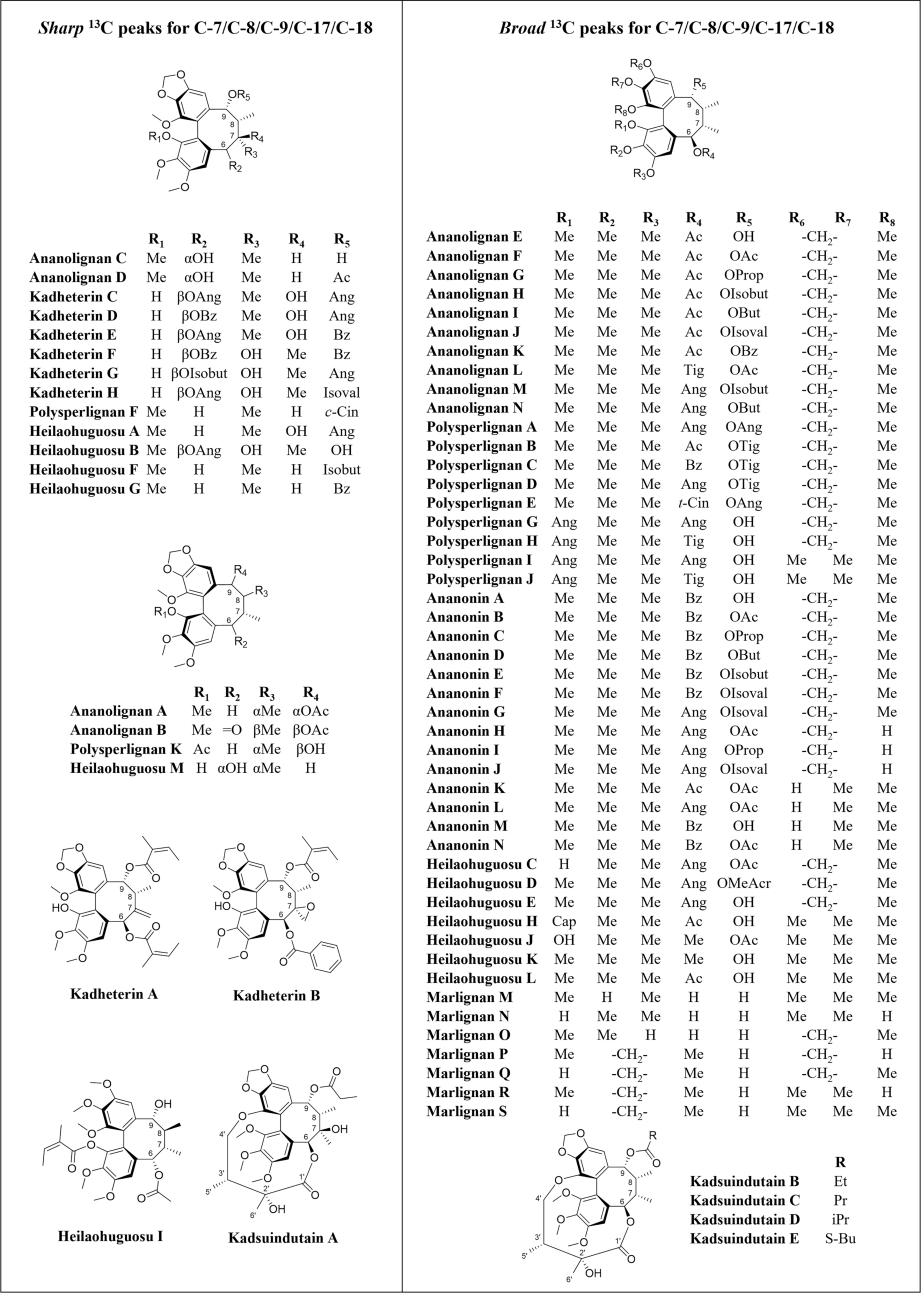
Seventy-one previously isolated dibenzocyclooctadienes from the Schisandraceae. Compounds on the left show (21) typical ^13^C NMR spectra, with all peaks sharp and clearly detectable, while those on the right (50) show broadened peaks associated with C-7/C-8/C-9/C-17/C-18. In the case of kandsuindutains B–E (bottom-right), the carbon resonances at C-1′/C-2′/C-3′/C-5′/C-6′ are also broadened or reduced in size.

Interestingly, in the case of kandsuindutains B-E, resonances at C-1′/C-2′/C-3′/C-5′/C-6′ are also broadened or reduced in size, indicating that cyclization of β-oriented C-6 substituents with one of the aromatic rings does not provide a stabilizing effect, while a C-7 substituent (kandsuindutain A) does.

To investigate the mechanisms behind this signal broadening, a computational study was conducted. Eleven dibenzocyclooctadienes were chosen, comprising five model compounds (Figure 6A) and six natural products (Figure 6C,D). The natural products included three with well-resolved ^13^C resonances (ananolignan A, C, and heilaohuguosu B) and three with broadened ^13^C resonances (ananolignan E, marlignan O, and kadheterin J).

**Figure 6:**
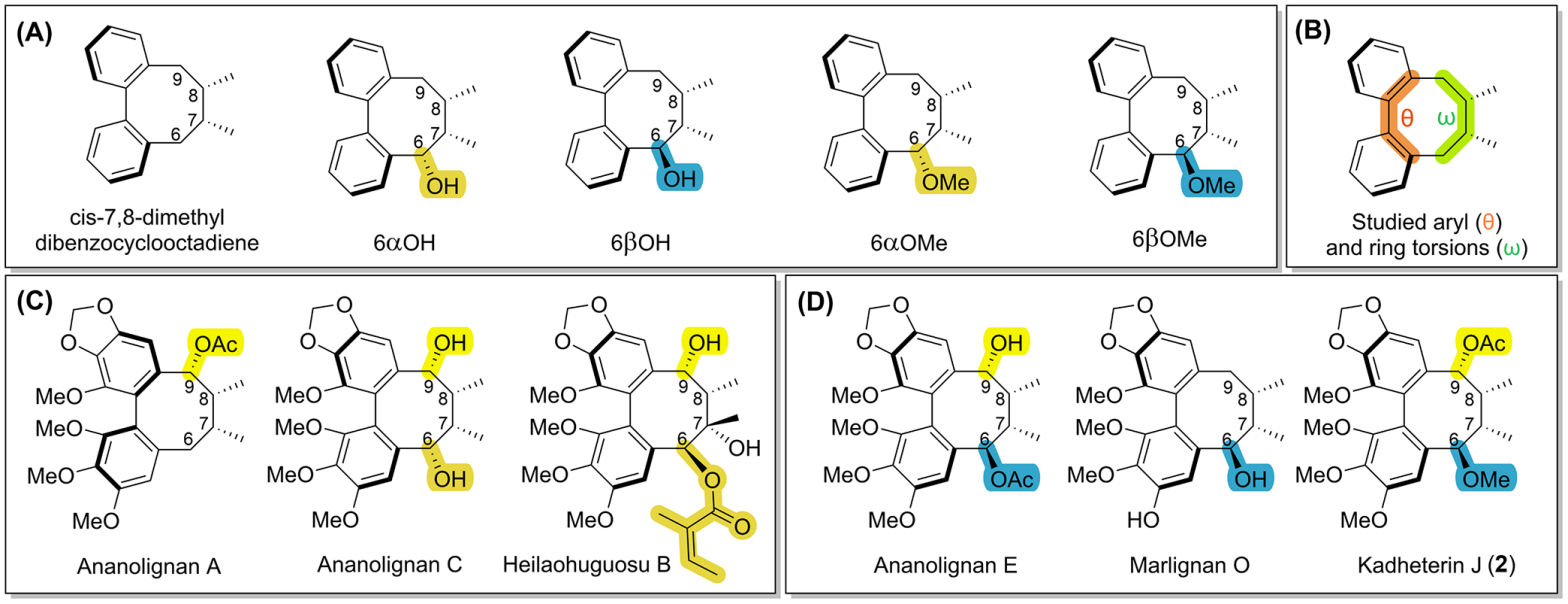
Structures used for computational investigation. (A) Model compounds. (B) Key aryl and ring torsions used for torsional energy scans and metadynamics-based conformer sampling. (C) Selected natural products with sharp ^13^C resonances at C-7/C-8/C-9/C-17/C-18. (D) Selected natural products with broadened ^13^C resonances at C-7/C-8/C-9/C-17/C-18.

The computational analyses on the model compounds in Figure 6A were based on DFT geometry optimizations run at B3LYP-D3BJ/6–31+G­(d,p) level, which were used to obtain the local minima with TBC and TB ring conformations. Torsional energy scans at 10° steps along the C-6/C-7/C-8/C-9 dihedral (ω, Figure 6B) were then performed at the same level, using the procedure described in the Computational Section. Our analysis started with the achiral parent compound *cis*-7,8-dimethyldibenzocyclooctadiene, which exists as two enantiomeric pairs of TBC and TB conformers (Figure 7A), the latter being less stable by Δ*H* ^0^ = 2.09 kcal/mol (2.8% population at 300 K). Analysis of noncovalent interactions (NCI)^21^ highlights the key role played by repulsive (steric) interactions between 6αH and 8βH with the phenyl rings in the TB conformer (orange areas indicated by red arrows in Figure 7C). Introduction of a 6αOH or 6αOMe group further stabilizes the TBC conformation, resulting from the additional gauche interaction between the new substituent and α-oriented 17-Me (S58 and S59). The introduction of a 6βOH group has two consequences: the first is the existence of different rotamers of the C–OH bond (Figure 7D), some of which benefit from OH-π interactions. The second is the relative stabilization of TB conformer (reaching 11.5 and 30.3% population according to Δ*H* ^0^ and Δ*G* ^0^, respectively) due to steric clashes involving the 6βOH substituent in the TBC conformers (Figure 7D). In the case of the 6βOMe group, a sizable population of TB conformer was also observed (12% from Δ*H* ^0^, S59). Summarizing the results on the models depicted in Figure 6A: (a) the parent system has an intrinsic tendency toward the TBC conformation; (b) 6αOH/6αOMe substituents make the TB conformation totally nonpopulated; (c) 6βOH/6βOMe substituents make the TB conformation sizably populated.

**Figure 7:**
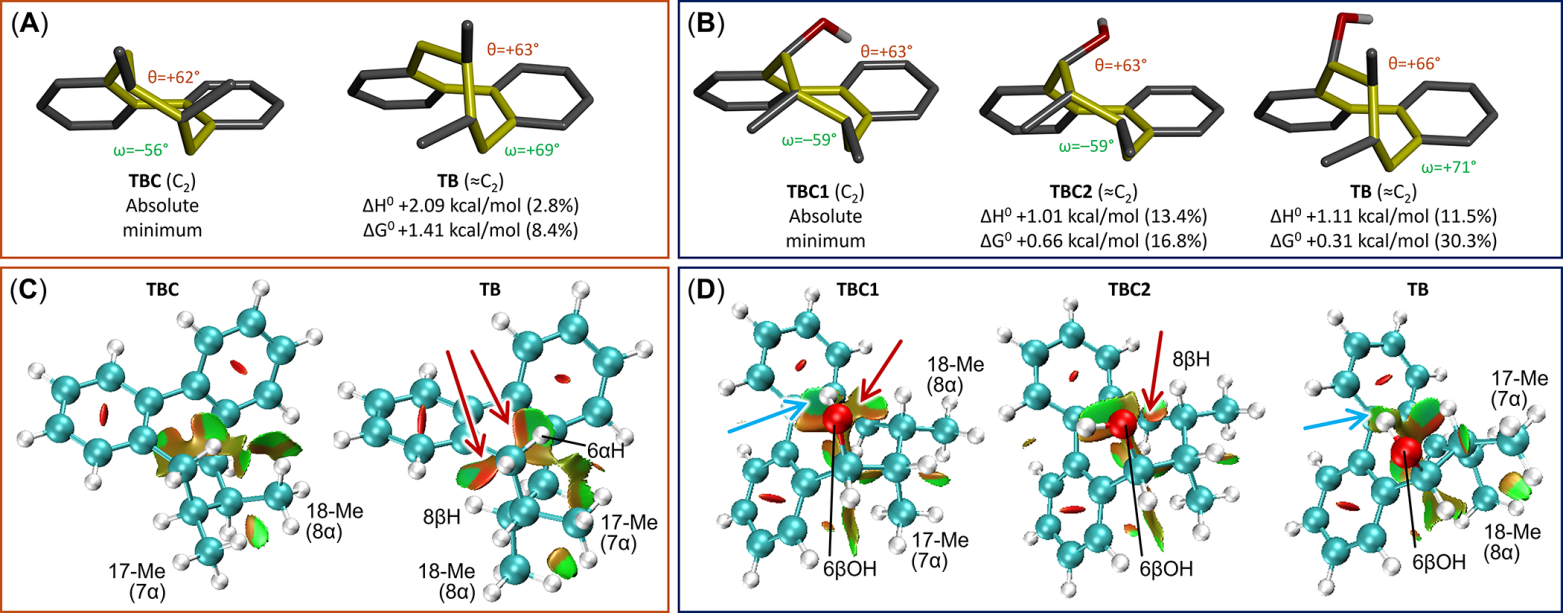
(A) DFT-optimized low-energy structures of *cis*-7,8-dimethyldibenzocyclooctadiene with relative enthalpies, free energies and populations at 300 K. The symmetry of the cyclooctadiene rings is indicated. (B) Same as (A), for (6*R*,7*S*,8*S*)-6-hydroxy-7,8-dimethyldibenzocyclooctadiene (6βOH). (C, D) Noncovalent interactions (NCI) analysis showing repulsive (steric) interactions as orange areas indicated by red arrows, van der Waals interactions as green areas, and attractive OH-π interactions as cyan areas indicated by cyan arrows.

Because of the many possible rotamers associated with e.g., methoxyl substituents, in the case of the natural products (Figure 6C,D), a conformational search was first run using the conformer–rotamer sampling tool (CREST)^22^ combined with the semiempirical tight-binding GFN2-xTB method.^23^ Looking at ω and θ dihedrals (Figure 6B), metadynamics results revealed a narrow distribution of values around two minima for θ, corresponding to opposite axial chirality, and four minima for ω, corresponding for each diastereomer to TBC and TB ring conformations. Graphical data from the torsional energy scans for ananolignan A and E are shown in Figure 8, while data for the other compounds are available in the SI (S60–S64). After discarding structures with the incorrect axial chirality, all remaining conformers found by CREST were screened by single-point DFT calculations and then optimized at B3LYP-D3BJ/6–31+G­(d,p) level. The two lowest-energy TBC/TB pairs with consistent orientation of the remaining substituents (OMe, OH, OAc and OAng) were then employed for torsional energy scans (B3LYP-D3BJ/6–31+G­(d,p)) with 10° steps along ω. Again, Figure 8 shows the results for ananolignan A and E, while other compounds (ananolignan C, marlignan O, heilaohuguosu B, kadheterin J (**2**), and kadsuphilin D) are summarized in Table 1 and reported in more detail (including CREST dynamics and torsional energy scans) in the SI (S60–S64). From these data, the following conclusions can be drawn:

(1) For ananolignan A and other analogues with well-resolved ^13^C NMR spectra, metadynamics display a clear preference for the TBC conformation, which is confirmed by DFT geometry optimizations and energy scans. TB minima had much higher energies than the corresponding TBC conformers (Δ*H* ^0^ = +4.9, +6.0, and +10.9 kcal/mol).
(2) For ananolignan E and other analogues with poorly resolved ^13^C NMR spectra, metadynamics display both populated TBC and TB families. DFT confirms that the relative energies of the TB minima were closer to the corresponding TBC conformer (Δ*H* ^0^ = +0.02, +0.7, and +2.2 kcal/mol).
(3) The associated barriers are between ∼11 and 12 kcal/mol in each case, corresponding to a rate of interconversion of 6 × 10^4^ to ∼ 10^3^ s^–1^ (0.02 to 1 ms) if Δ*S* ^‡^ ≈ 0 is assumed.

**Figure 8:**
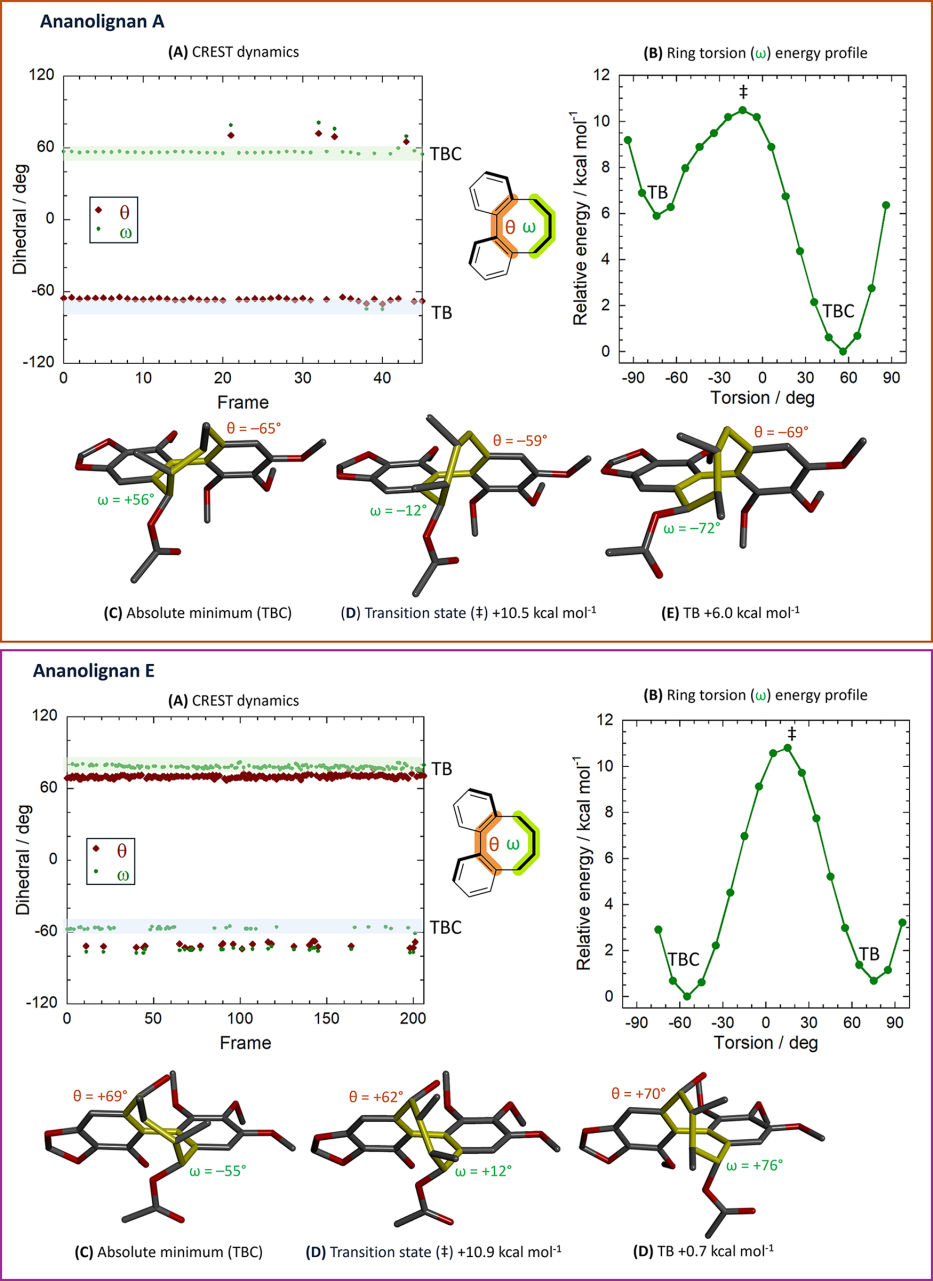
(A) Distribution of angles θ and ω along the CREST dynamics of ananolignan A (top) and E (bottom). The colored stripes highlight the two ranges of values assumed by angle ω for the correct sign of θ (negative for ananolignan A, positive for ananolignan E). (B) Ring torsion energy profiles at 10° steps along C-6/C-7/C-8/C-9 (relaxed scans at the B3LYP-D3BJ/6–31+G­(d,p) level). Labelled minima and maxima correspond to structures below. (C–E) DFT-optimized structures (B3LYP-D3BJ/6–31+G­(d,p)) for the lowest energy conformers and the transition state, with relative internal energies.

**Table 1:** Data (Δ*H* ^0^) from Ring Torsion Energy Scans Starting from the Lowest Energy Conformers (all TBC) for the Six Model Natural Products

compound	transition state energy vs absolute minimum (kcal/mol)	TB energy vs absolute minimum (kcal/mol)
ananolignan A	+10.5	+6.0
ananolignan C	+11.8	+4.9
heilaohuguosu B	+14.1	+10.9
ananolignan E	+10.9	+0.7
marlignan O	+11.0	+0.02
kadheterin J (**2**)	+12.1	+2.2
kadsuphilin D	+13.3	+4.1

Non-covalent interaction (NCI) analyses were run on TBC and TB conformers of ananolignan A and E and they are reported in the SI (Figure S65). These data consolidate the observations made for model compounds (Figure 7). In ananolignan A, TB conformer, the core of the eight membered ring is quite crowded, also with a contribution from the α-oriented 18-Me group. In ananolignan E, both TBC and TB conformers display a crowded core region, with contributions from the 18-Me group in the TBC conformer and the 17-Me group in the TB conformer; an attractive OH-π interaction is possible in both conformers.

After collating our findings, we noticed that the subset of studied dibenzocyclooctadienes (Figure 5) lacked examples of compounds bearing both (a) a β-oriented group at C-6 and (b) an additional substituent at C-8. To assess whether these substituents had the same TBC-stabilizing effect as those at C-7 (e.g., as in heilaohuguosu B), we searched for published examples of such a case. A substructure search using Dictionary of Natural Products revealed these to be rare, however a set of compounds from 2007 ^24^ and 2008 ^25^ with two examples fulfilling these criteria was found (kadsuphilins D and F, Figure 9). Unfortunately, no supporting ^13^C NMR spectra were available for these. Nonetheless, our structural analysis on kadsuphilin D showed a ring torsion energy profile and metadynamics results similar to ananolignans A, C, and heilaohuguosu B, showing that C-8 substituents have a TBC-stabilizing effect (Figure S64 and Table 1). In the TB form, C-17 adopts gauche relationships with both C-18 and the C-8 hydroxyl, resulting in significant steric strain. In contrast, the TBC form places C-17 gauche to C-18 but near-anti to the C-8 hydroxyl, relieving steric congestion.

**Figure 9:**
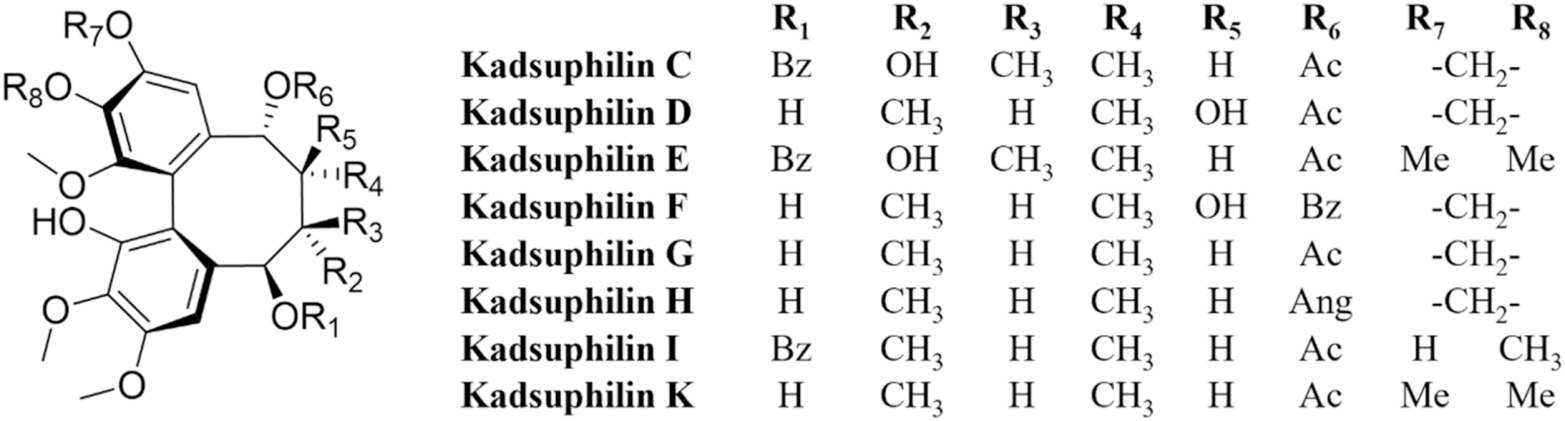
Structures of the kadsuphilins.

To evaluate the activation barrier to the interconversion process and thus experimentally validate the calculations in Table 1, we performed VT-NMR experiments for **1**-**3**, recording ^1^H NMR spectra at five-degree intervals from 241 to 301 K (Figures S51–S56). Due to signal overlap and spectrometer temperature limitations, we were unable to confidently determine the activation barrier for **1**. However, the activation barriers for **2** and **3** were estimated to be ∼13.1 and 13.2 kcal/mol, respectively, calculated using the Sandström version of the modified Eyring equation,^26,27^ in good agreement with DFT-calculated barriers (Table 1). The coalescence temperature for the broadened signals was observed at c.a. 276 K, consistent with the expected NMR time scale for conformational exchange.

Summarizing our investigation (points 1–4) and collating our findings with those of Gottlieb et al. (points 4–5),^11^ the five general rules for conformational dynamics, defined using (a*S*)-dibenzocyclooctadienes as a reference point, are

(1) β-Oriented (*endo*-) substituents at C-6 destabilize the TBC form through steric clashes with the proximal aryl ring, promoting TB/TBC exchange and causing ^13^C NMR signal broadening for C-7/C-8/C-9/C-17/C-18 (e.g., as ananolignan E, Figure 6)
(2) α-Oriented (*exo*-) substituents at C-6 stabilize the TBC form (e.g., as ananolignan C, Figure 6)
(3) Substituents at C-7 and C-8 stabilize the TBC form, even in the case of (1) (e.g., as heilaohuguosu B, Figure 5 and kadsuphilin D, Figure 9).
(4) α-Oriented substituents at C-9, as present in 86 of 88 reported compounds (Figures 2, 5, and 9), appear to have limited effect on ring conformation, since they are *endo-*oriented in both TBC and TB forms and thus little steric relief is furnished upon conformational conversion.^11^
(5) Benzylic carbonyls (ring ketones) stabilize the TB form if conjugation with the adjacent aryl ring is geometrically permitted. Evidence of this conjugation is clear from NMR data.^11^

In additional to the new compounds **1**-**3**, the known dibenzocyclooctadienes ananolignan D (**4**), ananolignan L (**5**),^17^ kadsurin (**6**), 9-hydroxykadsurin (**7**)^28^ were also isolated from *K. heteroclita*. Compound **7** has been reported twice synthetically, but is reported herein for the first time as a natural product, possibly as an artifact from hydrolysis of **6**.^29^ Using this as an opportunity to investigate the implications of C-6 substituents on bioactivity—and indeed to achieve the initial purpose of the chemical investigation—the anti-inflammatory activities of **1**-**7** were tested against our custom panel of cell reporter assays, each reflecting an inflammatory pathway relevant to rheumatoid arthritis (Table 2).^15^ Those without C-6 substituents (**6** and **7**) showed stronger inhibition of the pro-inflammatory NFAT signaling pathway (4 and 18 μM, respectively) than those with C-6 substituents (**1**-**5**, all 29 μM), highlighting the potential functional importance of this position.

**Table 2:** Anti-Inflammatory Activities of Dibenzocyclooctadienes (**1**-**7**) from *K. heteroclita* ^a^

	IC_50_ (μM)				
	SW982-NF-κB	Jurkat-NF-κB	Jurkat-NFAT	HEK293-STAT3	U937-STAT5
**1**		25–50^2^	29^3^		n.e.^1^
**2**		n.e.^1^	29^3^		n.e.^1^
**3**		n.e.^1^	n.e.^1^		n.e.^1^
**4**	n.e.^1^	n.e.^1^	n.e.^3^	n.e.^1^	n.e.^1^
**5**		50^2^	≥50^3^		n.e.^1^
**6** ^a^	n.e.^1^	43^4^	4^4^	75–100^2^	48^4^
**7**	n.e.^1^	n.e.^1^	18^4^	n.e.^1^	n.e.^1^

a Numbers in superscript denote number of replicates. n.e. = no effect at 50 μM. ^a^Tested up to 100 μM. Information on positive controls and stimuli are provided in the SI (Table S3).

## Conclusions

This study reveals a previously overlooked yet widespread feature in the ^13^C NMR spectra of dibenzocyclooctadienes: signal broadening at specific cyclooctadiene carbon resonances. Our investigation of this phenomenon through literature studies, DFT calculations, and VT-NMR experiments, has demonstrated that this is caused by key *endo-*oriented benzylic substituents that destabilize the TBC conformation through steric clashes with proximal aryl rings. We have distilled our findings into five simple rules that link substituent orientation to conformational behavior. These rules are applicable to the most common dibenzocyclooctadiene scaffold and substituents. Our findings resolve a common misunderstanding in structural assignments of these compounds, and we have found that many dibenzocyclooctadienes previously described as adopting discrete TBC or TB conformations are actually interconverting mixtures. These findings thus have implications on structural interpretations of hundreds of published compounds. Given how elusive and subtle conformational isomerism can be, we encourage chemists to explore the three-dimensional behavior of their compounds through conformational searching, which can be performed using easily accessible tools, including both freely available (e.g., CREST)^22^ and commercial software.

## Experimental Section

### General Experimental Procedures

Optical rotation data were acquired using a PerkinElmer 241 polarimeter (PerkinElmer, Waltham, MA) and [α]_D_ values are given in 10^–1^ deg cm^2^ g^–1^. UV data were obtained using a NanoDrop One spectrophotometer (Thermo Fisher Scientific, Wilmington, DE). ECD data were recorded with a J-810 CD spectrometer (Jasco, Tokyo, Japan). NMR spectra were acquired using a Bruker Avance Neo 600 MHz (TCI CRPHe TR-1H and 19*F*/13*C*/15N 5 mm-EZ CryoProbe) and a Bruker Avance Neo 500 MHz spectrometer (TXO CRPHe TR-^1^H/^13^C/^15^N 5 mm-Z) spectrometer (Bruker, Billerica, MA). Chemical shifts were referenced to the solvent peak for CDCl_3_ at δ_H_ 7.26/δ_C_ 77.16 and (CD_3_)_2_SO at δ_H_ 2.50/δ_C_ 39.52. High-resolution ESI-MS data were collected on a Waters Xevo G2-XS quadrupole time-of-flight mass spectrometer (Waters Corp. Milford, MA). MPLC was performed with a Varian Pro Star pump (Varian, Crawley, UK), preparative HPLC with a Shimadzu LC-10, and semipreparative HPLC with a Shimadzu LC-20 (Shimadzu, Kyoto, Japan). Acetonitrile (99.9%), dichloromethane (99.9%), methanol (99.9%), chloroform-*d* (99.9%), and DMSO-*d*_6_ (99.8%) were from VWR (VWR International, Radnor, PA). Water was Millipore Milli-Q PF filtered and TFA was acquired from Iris Biotech GmBH (Marktredwitz, Germany). HPLC columns and C_18_ silica gel used to adsorb extracts before HPLC (Sepra C18-E, 50 μm, 65 Å) were from Phenomenex (Torrance, CA). Diol-bonded silica MPLC columns were from Interchim (Montluçon, France) and silica gel used to adsorb samples prior to MPLC was from Merck (Rahway, NJ).

### Plant Material

*Kadsura heteroclita* material was provided by the Guangdong Provincial Hospital of Chinese Medicine, China and produced by Kangmei Pharmaceutical Co. Ltd., Guangdong, in December 2022 (batch number 221200221). Plant material stored at room temperature prior and ground was ground into a powder using a knife mill before extraction.

### Extraction and Isolation

*K. heteroclita* stem bark (500 g) was extracted in CH_2_Cl_2_:MeOH (1:1) (5 L) overnight on an orbital shaker and the extract filtered and evaporated to dryness to yield 22 g of crude extract. This extract was partitioned between CH_2_Cl_2_ (1200 mL) and H_2_O (800 mL). The organic phase (20 g) was purified four times (4 × 5g) using a diol-bonded silica column (Interchim PF-DIOL, 30 μm, 40 g) into seven fractions, each eluted with 160 mL of 100% hexane, hexane-EtOAc (5:1), hexane-EtOAc (1:1), hexane-EtOAc (3:7), 100% EtOAc, EtOAc-MeOH (7:3) and 100% MeOH. The hexane-EtOAc (1:1) fraction (10 g) was purified using repeated preparative HPLC (10 × 1 g) (Kinetex XB-C_18_, 5 μm, 100 Å, 150 × 21.2 mm) using a linear gradient from 60% CH_3_CN (0.1% TFA) to 100% CH_3_CN (0.1% TFA) over 35 min at a flow rate of 9 mL/min. The fractions collected between 14 and 17 min (370 mg) were recombined and repurified using semipreparative HPLC (Kinetex XB-C_18_, 5 μm, 100 Å, 250 × 10.0 mm) using a gradient from 70% CH_3_CN (0.1% FA) to 75% CH_3_CN (0.1% FA) over 30 min, then to 100% CH_3_CN (0.1% FA) over mins 30–35, and held at 100% CH_3_CN (0.1% FA) from mins 35–40 at a flow rate of 4 mL/min. Fractions eluting between 3 and 5 min (12 mg) and 6–7 min (8 mg) were separately recombined, and the fraction eluting at 9 min contained **1** (4 mg). The fractions eluting between 3 and 5 min (12 mg) were repurified using semipreparative HPLC (Kinetex XB-C_18_, 5 μm, 100 Å, 250 × 10.0 mm) using isocratic 35% CH_3_CN (0.1% FA) over 25 min at a flow rate of 4 mL/min. Compound **3** (4 mg) eluted between mins 21–25. The fractions eluting between 6 and 7 min (8 mg, as above) were purified using HPLC (Kinetex Biphenyl, 5 μm, 100 Å, 250 × 10.0 mm) using isocratic 48% CH_3_CN (0.1% FA) over 30 min, and the fractions eluting between 23 and 24 min contained **2** (4 mg).

#### Kadheterin I (**1**)

yellow, amorphous solid; [α]_D_^25^ + 74 (*c* 0.28, MeOH); UV (MeOH) λ_max_ (log ε) 285 (3.37), 254 (3.86), 230 (4.36) nm; ECD (*c* 0.034 mM), λ_max_ MeOH (Δ*ε*) 284 (−2.1), 252 (−9.8), 223 (+15.8) nm; ^1^H and ^13^C NMR data, Table S1; (+)-HRESIMS *m*/*z* 581.2363 [M + Na]^+^ (calcd for C_30_H_38_NaO_10_^+^, 581.2363)

#### Kadheterin J (**2**)

yellow, amorphous solid; [α]_D_^25^ + 79 (*c* 0.29, MeOH); UV (MeOH) λ_max_ (log ε) 285 (3.39), 254 (4.00), 230 (4.56) nm; ECD (*c* 0.019 mM), λ_max_ MeOH (Δ*ε*) 284 (−2.0), 252 (−9.9), 223 (+13.1) nm; ^1^H and ^13^C NMR data, Table S1; (+)-HRESIMS *m*/*z* 511.1946 [M + Na]^+^ (calcd for C_26_H_32_NaO_9_^+^, 511.1944)

#### Kadheterin K (**3**)

yellow, amorphous solid; [α]_D_^25^ + 60 (*c* 0.24, MeOH); UV (MeOH) λ_max_ (log ε) 285 (3.11), 254 (3.72), 230 (4.20) nm; ECD (*c* 0.038 mM), λ_max_ MeOH (Δ*ε*) 285 (−1.3), 255 (−5.9), 227 (+11.8), 206 (+14.6) nm; ^1^H and ^13^C NMR data, Table S1; (+)-HRESIMS *m*/*z* 538.2052 [M + Na]^+^ (calcd for C_27_H_33_NNaO_9_^+^, 538.2053)

### Computational Details

Conformational analyses were run by CREST (v. 3.0.2) using the GFN2-xTB semiempirical method and the GBSA solvation model for CHCl_3_. Default parameters were employed for CREST calculations, including ″normal″ accuracy for the final geometry optimization, automatically set simulation length, SHAKE mode for all bonds, and 5 fs time step. The structures with the correct axial chirality were retained. Then, the resulting conformational ensembles were screened using single-point energy calculations at the B3LYP-D3/6–31G­(d) level and those within an energy window of 5 kcal/mol kept. The selected ensembles were then subjected to an initial geometry optimization at the B3LYP-D3BJ/6–31G­(d) level. Final geometry optimizations, vibrational frequency calculations, and torsional energy scans were performed at the B3LYP-D3BJ/6–31+G­(d,p) level. Torsional energy scans were run in forward and backward directions starting from TBC and TB minima. The energy maxima were then optimized as transition points (one imaginary frequency). All DFT calculations were run using Gaussian16 software.^30^ NCI plots were generated using MultiWfn 3.8.^31,32^

### Generation and Stimulation of Cell Reporter Cell Lines

Cell lines were prepared as previously described.^15^ SW982-NF-κB and HEK293-STAT3 cells were seeded the day before the experiment in flat bottom 96-well plates to allow the cells to attach. Jur-NF-κB, Jurkat-NFAT and U937-STAT5 cells were seeded the day of the experiment in round-bottom 96-well plates. Cell lines were treated with the compounds in a concentration range up to 50 μM and stimulated to specifically activate the pathway that was analyzed. A pathway specific inhibitor was included as a control for the assay. All cells were incubated for 24 h at 0.5% DMSO, with the exception of Jurkat-NFAT cells, which were incubated for 16 h at 1% DMSO.

### Flow Cytometry

After incubation, all cell lines were centrifuged and resuspended in MACS Buffer (autoMACS Running Buffer, Miltenyi Biotec) with the fluorescent viability dye 7-Amino-Actinomycin D (7-AAD, BD Biosciences, 1:100 dilution) and run on a flow cytometer (FACSVerse, BD Biosciences and CytoFlex, Beckman Coulter). Cells were analyzed for viability (7-AAD negative) and pathway activity (GFP positive). GFP positivity was determined with the help of the unstimulated cells. FACS data was first analyzed with FlowJo to adjust the gates. Afterward, cell viability and GFP positivity were normalized to the average of the stimulated cells with the same DMSO concentration as the sample wells. If less than 1000 viable cells were recorded, the GFP signal was considered not reliable. Normalized GFP data was imported into GraphPad Prism and the absolute IC_50_ was calculated. Samples with a viable cell count <1000 were excluded from the calculations. Information on positive controls used is available in the SI (Table S3).

## Supplementary Material



## Notes

Detailed structural characterization, ECD, and NMR spectral data of 1–3, additional computational results, and anti-inflammatory assay control data (PDF)
