# Supplementary material for: Rules for Dibenzocyclooctadiene Conformational Dynamics
Source: J Nat Prod. 2026 Jan 31;89(2):528–38. doi: 10.1021/acs.jnatprod.5c01348 (PMC12954843; doi:10.1021/acs.jnatprod.5c01348)
Supplement: Supplementary file 1 [file np5c01348_si_001.pdf]

Supporting Information

Rules for Dibenzocyclooctadiene Conformational Dynamics

Luke P. Robertson,[1,*] Wen Xu,[2] Louisa Brieskorn,[3] Iro Chaitoglou,[3] Jing Guo,[1] Runyue Huang,[4] Per-Johan Jakobsson,[3] Gennaro Pescitelli,[5] Ulf Göransson[1,*]

[1]Pharmacognosy, Department of Pharmaceutical Biosciences, Uppsala University, Uppsala, Sweden;

[2]State Key Laboratory of Traditional Chinese Medicine Syndrome, The Second Clinical College, Guangzhou University of Chinese Medicine, Guangzhou, China;

[3]Division of Rheumatology, Department of Medicine, Solna, Karolinska Institutet, and Karolinska University Hospital, Solna, and Center for Molecular Medicine, Karolinska University Hospital, Stockholm, Sweden;

[4]Section of Rheumatology and Immunology Research, The Second Affiliated Hospital of Guangzhou University of Chinese Medicine (Guangdong Provincial Hospital of Chinese Medicine), Guangzhou, China

[5]Dipartimento di Chimica e Chimica Industriale, Università di Pisa, Via Giuseppe Moruzzi, 13, Pisa, 56124, Italy.

*Luke P. Robertson: luke.robertson@uu.se

*Ulf Göransson: ulf.goransson@uu.se

Table of Contents

P4-P63. Section 1. Structural characterisation of **1-3**

P4. Figure S1. Key HMBC (arrows) and COSY (bold bonds) correlations in **1**.
P6. Table S1. NMR Data for Kadheterin I (**1**), Kadheterin J (**2**) and Kadheterin K (**3**) at 323 K
P7. Table S2. NMR Data for Kadheterin K (**3**) in DMSO-d_6 and $CDCl_3$
P8. Figure S2. ^{1}H NMR spectrum of **1** in chloroform-*d* at 323K (500 MHz)
P9. Figure S3. Zoomed ^{1}H NMR spectrum of **1** in chloroform-*d* at 323 K (500 MHz)
P10. Figure S4. ^{13}C NMR spectrum of **1** in chloroform-*d* at 323 K (125 MHz)
P11. Figure S5. Zoomed ^{13}C NMR spectrum of **1** in chloroform-*d* at 323 K (125 MHz)
P12. Figure S6. ^{13}C NMR spectra of **1** in chloroform-*d* at 323 K and 298 K (125 MHz)
P13. Figure S7. COSY NMR spectrum of **1** in chloroform-*d* at 323 K (500 MHz)
P14. Figure S8. Zoomed COSY NMR spectrum of **1** in chloroform-*d* at 323 K (500 MHz)
P15. Figure S9. HSQC NMR spectrum of **1** in chloroform-*d* at 323 K (500 MHz)
P16. Figure S10. Zoomed HSQC NMR spectrum of **1** in chloroform-*d* at 323 K (500 MHz)
P17. Figure S11. HMBC NMR spectrum of **1** in chloroform-*d* at 323 K (500 MHz)
P18. Figure S12. Zoomed HMBC NMR spectrum of **1** in chloroform-*d* at 323 K (500 MHz)
P19. Figure S13. NOESY NMR spectrum of **1** in chloroform-*d* at 323 K (500 MHz)
P20. Figure S14. Zoomed NOESY NMR spectrum of **1** in chloroform-*d* at 323 K (500 MHz)
P21. Figure S15. ^{1}H NMR spectrum of **2** in chloroform-*d* at 323 K (500 MHz).
P22. Figure S16. Zoomed ^{1}H NMR spectrum of **2** in chloroform-*d* at 323 K (500 MHz).
P23. Figure S17. ^{13}C NMR spectrum of **2** in chloroform-*d* at 323 K (500 MHz).
P24. Figure S18. Zoomed ^{13}C NMR spectrum of **2** in chloroform-*d* at 323 K (500 MHz).
P25. Figure S19. COSY NMR spectrum of **2** in chloroform-*d* at 323 K (500 MHz)
P26. Figure S20. Zoomed COSY NMR spectrum of **2** in chloroform-*d* at 323 K (500 MHz)
P27. Figure S21. HSQC NMR spectrum of **2** in chloroform-*d* at 323 K (500 MHz)
P28. Figure S22. Zoomed HSQC NMR spectrum of **2** in chloroform-*d* at 323 K (500 MHz)
P29. Figure S23. HMBC NMR spectrum of **2** in chloroform-*d* at 323 K (500 MHz)
P30. Figure S24. Zoomed HMBC NMR spectrum of **2** in chloroform-*d* at 323 K (500 MHz)
P31. Figure S25. NOESY NMR spectrum of **2** in chloroform-*d* at 323 K (500 MHz)
P32. Figure S26. Zoomed NOESY NMR spectrum of **2** in chloroform-*d* at 323 K (500 MHz)
P33. Figure S27. ^{1}H NMR spectrum of **3** in DMSO-d_6 at 323 K (500 MHz)
P34. Figure S28. Zoomed ^{1}H NMR spectrum of **3** in DMSO-d_6 at 323 K (500 MHz)
P35. Figure S29. ^{13}C NMR spectrum of **3** in DMSO-d_6 at 323 K (125 MHz)
P36. Figure S30. Zoomed ^{13}C NMR spectrum of **3** in DMSO-d_6 at 323 K (125 MHz)
P37. Figure S31. COSY NMR spectrum of **3** in DMSO-d_6 at 323 K (500 MHz)
P38. Figure S32. Zoomed COSY NMR spectrum of **3** in DMSO-d_6 at 323 K (500 MHz)
P39. Figure S33. HSQC NMR spectrum of **3** in DMSO-d_6 at 323 K (500 MHz)
P40. Figure S34. Zoomed HSQC NMR spectrum of **3** in DMSO-d_6 at 323 K (500 MHz)
P41. Figure S35. HMBC NMR spectrum of **3** in DMSO-d_6 at 323 K (500 MHz)
P42. Figure S36. Zoomed HMBC NMR spectrum of **3** in DMSO-d_6 at 323 K (500 MHz)
P43. Figure S37. NOESY NMR spectrum of **3** in DMSO-d_6 at 323 K (500 MHz)
P44. Figure S38. Zoomed NOESY NMR spectrum of **3** in DMSO-d_6 at 323 K (500 MHz)
P45. Figure S39. ^{1}H NMR spectrum of **3** in $CDCl_3$ at 298 K (600 MHz)
P46. Figure S40. Zoomed ^{1}H NMR spectrum of **3** in $CDCl_3$ at 298 K (600 MHz)
P47. Figure S41. ^{1}H NMR spectrum of **3** in $CDCl_3$ at 323 K (500 MHz)
P48. Figure S42. Zoomed ^{1}H NMR spectrum of **3** in $CDCl_3$ at 323 K (500 MHz)
P49. Figure S43. ^{13}C NMR spectrum of **3** in $CDCl_3$ at 323 K (125 MHz)
P50. Figure S44. Zoomed ^{13}C NMR spectrum of **3** in $CDCl_3$ at 323 K (125 MHz)
P51. Figure S45. HSQC NMR spectrum of **3** in $CDCl_3$ at 323 K (500 MHz)

P52. Figure S46. Zoomed HSQC NMR spectrum of **3** in $CDCl_3$ at 323 K (500 MHz)
P53. Figure S47. HMBC NMR spectrum of **3** in $CDCl_3$ at 323 K (500 MHz)
P54. Figure S48. Zoomed HMBC NMR spectrum of **3** in $CDCl_3$ at 323 K (500 MHz)
P55. Figure S49. NOESY NMR spectrum of **3** in $CDCl_3$ at 323 K (500 MHz)
P56. Figure S50. Zoomed NOESY NMR spectrum of **3** in $CDCl_3$ at 323 K (500 MHz)
P57. Figure S51. ^{1}H VT-NMR data for **2** in $CDCl_3$ (241–301 K) (500 MHz)
P58. Figure S52. Upfield zoomed ^{1}H VT-NMR data for **2** in $CDCl_3$ (241–301 K) (500 MHz)
P59. Figure S53. Downfield zoomed ^{1}H VT-NMR data for **2** in $CDCl_3$ (241–301 K) (500 MHz)
P60. Figure S54. ^{1}H VT-NMR data for **3** in $CDCl_3$ (241–301 K) (500 MHz)
P61. Figure S55. Upfield zoomed ^{1}H VT-NMR data for **3** in $CDCl_3$ (241–301 K) (500 MHz)
P62. Figure S56. Downfield zoomed ^{1}H VT-NMR data for **3** in $CDCl_3$ (241–301 K) (500 MHz)
P63. Figure S57. ECD spectra of **1–3**

P64. Section 2a: Computational chemistry, calculation results for model compounds

P64. Figure S58. DFT results for (6*S*,7*S*,8*S*)-6-hydroxy-7,8-dimethyldibenzo cyclooctadiene
P64. Figure S59. DFT results for (6*S*,7*S*,8*S*)-6-methoxy-7,8-dimethyldibenzo cyclooctadiene

P65-70. Section 2b: Computational chemistry, calculation results for natural products

P65. Figure S60. CREST dynamics and ring torsion energy scan results for ananolignan C
P66. Figure S61. CREST dynamics and ring torsion energy scan results for marlignan O
P67. Figure S62. CREST dynamics and ring torsion energy scan results for heilaohuguosu B
P68. Figure S63. CREST dynamics and ring torsion energy scan results for kadheterin J
P69. Figure S64. CREST dynamics and ring torsion energy scan results for kadsuphilin D
P70. Figure S65. NCI analysis for ananolignan A and E.

P71. Section 3: Anti-inflammatory activity testing

P71. Figure S66. General gating strategy for cell reporter assays.
P71. Table S3. Information for cell reporter assays.

P72. Section 4: References

Section 1. Structural characterisation of **1-3**.

Kadheterin I (**1**) (Figure S1) was isolated as a yellow amorphous solid. HRESIMS data in positive mode showed a molecular ion peak at *m*/*z* 581.2363 $[M + Na]^+$, from which a formula of $C_{30}H_{38}O_{10}$ was calculated. Analysis of its ^{1}H NMR data (Table S1) revealed signals characteristic of a dibenzocyclooctadiene lignan, including two aromatic singlets (δ_H 6.66, 6.44, 2H), four aromatic methoxyls (δ_H 3.88-3.58, 12H) and a methylenedioxyphenyl group (δ_H 5.98, 5.97, 2H). The ECD spectrum of **1** showed a positive cotton effect at λ_{max} 223 nm and a negative effect at λ_{max} 252 nm, indicating an *S*-biphenyl configuration.[1] The presence of two deshielded methines (δ_H 5.72/δ_C 80.4 and δ_H 5.78/δ_C 80.9) in the HSQC spectrum of **1** indicated C-6 and C-9 to be oxygenated, and this was supported by $^3J_{CH}$ HMBC correlations from H-6/H-9 to ester carbon resonances at δ_C 175.8/170.1, respectively. Shared $^3J_{CH}$ HMBC correlations between H-11 (δ_H 6.44) and the methylenedioxyphenyl group were used to assign these to the same ring, and thereafter $^3J_{CH}$ HMBC correlations between H-11/C-9 and H-4/C-6 used to assign the relative positions of the benzylic methines. An acetoxyl group (δ_H 1.58/δ_C 20.8) was determined to occur at C-9 through a $^2J_{CH}$ HMBC correlation from it to the carbonyl resonance at δ_C 170.1. Finally, unassigned resonances associated with a methyl doublet (δ_H 0.92, J = 7.0 Hz), a methyl triplet (δ_H 0.77, J = 7.0 Hz), a methylene (δ_H 1.24/1.44, m) and a methine (δ_H 2.01, m) indicated a 2-methylbutyryl group to be substituted on C-6. The position of this was confirmed by shared $^3J_{CH}$ HMBC correlations between H-6/C-1′ and from H-2′/H-3′/H-5′ to C-1′. With the planar structure of **1** established, the configurations of the stereogenic centres at C-6/C-7/C-8/C-9 in **1** were determined through analysis of NOESY data. NOE correlations between H-8/H-11 and H-9/H-11 indicated H-8/H-9 to be β-oriented, while correlations between H-4/H-6 and H-4/H-17 supported both H-6 and CH_3-17 to be α-oriented. Due to free rotation of the 2-methylbutyryl group, the configuration of C-2′ was not assigned. Thus, the absolute configuration was determined to be (*aS*,6R,7S,8R,9R)-**1**.

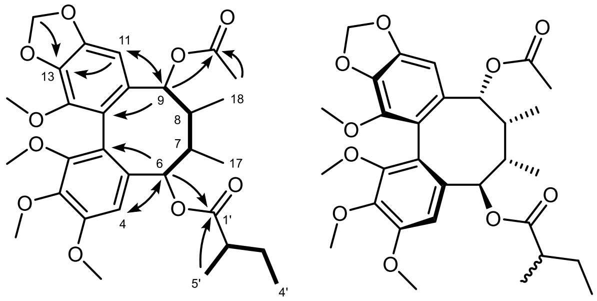

Figure S1. Key HMBC (arrows) and COSY (bold bonds) correlations in **1** (left), along with its absolute configuration (right).

Kadheterin J (**2**) was isolated as a yellow amorphous solid. A molecular ion peak in HRESIMS in positive mode at *m/z* 511.1946 $[M + Na]^+$ was used to assign a molecular formula of $C_{26}H_{32}O_9$ to **2**. The NMR data of **2** was similar to **1**, although the resonances from the 2-methylbutyryl group in **1** were absent and an additional methoxyl resonance was present (δ_H 3.04/δ_C 56.4). Shared $^3J_{CH}$ HMBC correlations between both this methoxyl resonance and H-4 (δ_H 6.50) to a methine carbon resonance (δ_C 90.2) were used to assign the methoxyl to C-6. A positive cotton effect at λ_{max} 223 nm and a negative effect at λ_{max} 252 nm suggests **2** to have an *S*-biphenyl configuration, and its optical rotation ($[\alpha]_D^{25}$ +79) confirms its absolute configuration to be as **1**.

Kadheterin K (**3**) was isolated as a yellow amorphous solid. The presence of an even molecular ion peak in HRESIMS in positive mode at *m/z* 538.2052 $[M + Na]^+$ suggested it to contain nitrogen and a molecular formula of $C_{27}H_{33}NO_9$ was calculated. Compound **3** showed very similar ^{1}H NMR data to **1**/**2**, however the methine group at C-6 (δ_H 5.72/δ_C 80.4 in **1**) was shifted upfield to at δ_H 4.67/δ_C 59.9. H-6 also appears as a doublet of doublets (J = 9.0, 9.0 Hz) in **3** and shows HMBC correlations to a carbonyl group at δ_C 167.6. Correlations from a methyl singlet (δ_H 1.63, δ_C 22.4) to this same carbonyl suggested that an acetamido group occurs at C-6, and thus the planar structure of **3** was established. The NMR data for **3** is reported in DMSO-d_6 (Table 1) as the acetamido carbonyl resonance was not observed in $CDCl_3$, nor could it be detected via $^3J_{CH}$ HMBC correlations from H-6. The data in $CDCl_3$, however, offered the benefit of clearer separation of H_3-17/H_3-18, which occur at δ_H 0.86/0.84 in DMSO-d_6, but δ_H 0.96/1.02 in $CDCl_3$. This facilitated investigation of the ring conformation of **3**, which were complicated by overlapping resonances of H_3-17/H_3-5′ (δ_H 0.91/0.92) in **1** and H_3-17/H_3-18 (δ_H 0.94/0.96) in **2**. The NOESY spectrum of **3** acquired in $CDCl_3$ showed a strong cross-peak between H-4/H_3-17, which would be observable in both conformations (interproton distances of 2.5 Å in TBC vs. 4.3 Å in TB) (Figure 7). However, weak cross-peaks between H-4/H_3-18 (5.5 Å in TBC vs. 2.9 Å in TB), H-6/H_3-18 (5.0 Å in TBC vs. 2.7 Å in TB), and H-7/H-11 (5.0 Å in TBC vs. 3.2 Å in TB) indicate some TB population. The predominance of the TBC form is supported by the intensity of the H-4/H_3-17 NOESY cross-peak, which is almost equal intensity with that of H-6/H_3-17 (2.4 Å in both TBC and TB). The ECD and optical rotatory data of **3** indicate it to have the same absolute configuration as **1**/**2**. Compound **3** is the first nitrogen-containing dibenzocyclooctadiene to be isolated from the Schisandraceae, and as such we suspected it to be an adduct formed during the isolation. The evidence does not suggest that **3** is an acetonitrile adduct from HPLC, however, as it could be detected using LC-MS in a

sample that had only undergone extraction in MeOH/CH_2Cl_2 (1:1) and purified using diol-bonded silica using hexane/EtOAc.

Table S1. NMR Data for Kadheterin I (**1**), Kadheterin J (**2**) and Kadheterin K (**3**) at 323 K

Position	Kadheterin I (**1**)[a]			Kadheterin J (**2**)[a]		Kadheterin K (**3**)[b]	
	δ_C[c], type	δ_H[d], mult (*J* in Hz)	HMBC[e]	δ_C[c], type	δ_H[d], mult (*J* in Hz)	δ_C[c], type	δ_H[d], mult (*J* in Hz)
1	151.8, C	-	-	152.0, C	-	150.6, C	-
2	141.7, C	-	-	141.5, C	-	140.0, C	-
3	152.0, C	-	-	151.7, C	-	151.3, C	-
4	111.0, CH	6.67, s	1, 2, 3, 5, 6, 16	111.3, CH	6.50, s	111.3, CH	6.77, s
5	131.5, C	-	-	132.5, C	-	134.4, C	-
6	80.9, CH	5.78, d (7.8)	4, 5, 7, 8, 16, 1′	90.2, CH	3.99, brm	57.9, CH	4.67, dd (9.0, 9.0)
7	38.4, CH	2.08, m	6, 7, 8, 9	40.1, CH	1.99, m	37.2, CH	1.71, m
8	39.6, CH	2.13, m	6, 7, 8, 9	38.8, CH	2.20, m	39.4, CH	2.14, m
9	80.4, CH	5.74, d (3.9)	7, 8, 10, 11, 15, Me*CO*-9	80.5, CH	5.73, d (4.6)	78.1, CH	5.69, d (6.0)
10	133.2, C	-	-	133.4, C	-	132.6, C	-
11	102.4, CH	6.43, s	9, 12, 13, 14, 15	102.5, CH	6.40, s	102.1, CH	6.60, s
12	148.8, C	-	-	148.6, C	-	148.1, C	-
13	136.3, C	-	-	136.1, C	-	135.5, C	-
14	142.1, C	-	-	141.9, C	-	141.5, C	-
15	121.7, C	-	-	121.4, C	-	120.5, C	-
16	123.6, C	-	-	123.7, C	-	121.9, C	-
17	16.2, CH_3	0.91, d (6.8)	6, 7, 8	16.6,[f] CH_3	0.94, d (6.8)	17.9,[f] CH_3	0.86, d (6.0)
18	n.o., CH_3	0.99, d (6.8)	7, 8, 9	16.6,[f] CH_3	0.96, d (6.8)	17.9,[f] CH_3	0.84, d (6.0)
1′	175.8, C	-	-	-	-	-	-
2′	41.3, CH	2.01, m	1′, 3′, 4′, 5′	-	-	-	-
3′	26.6, CH_2	1.44, m<br>1.24, m	1′, 2′, 4′, 5′<br>1′, 2′, 4′, 5′	-	-	-	-
4′	11.6, CH_3	0.77, t (7.0)	2′, 3′	-	-	-	-
5′	15.9, CH_3	0.92, d (7.0)	1′, 2′, 3′	-	-	-	-
OCH_3-1	60.3, CH_3	3.58, s	1	60.2, CH_3	3.59, s	59.4, CH_3	3.47, s
OCH_3-2	60.7, CH_3	3.88, s	3	60.8, CH_3	3.91, s	59.8, CH_3	3.79, s
OCH_3-3	56.3, CH_3	3.89, s	2	56.4, CH_3	3.90, s	55.8, CH_3	3.75, s
OCH_3-6	-	-	-	56.3, CH_3	3.04, s	-	-
NH-6	-	-	-	-	-	-	n.o.
*Me*CO-6	-	-	-	-	-	22.4, CH_3	1.63, brs
Me*CO*-6	-	-	-	-	-	167.6, C	-
*Me*CO-9	20.8, CH_3	1.58, s	Me*CO*-9	20.8, CH_3	1.58, s	20.0, CH_3	1.48, s
Me*CO*-9	170.1, C	-	-	170.2, C	-	169.0, C	-
OCH_3-14	59.5, CH_3	3.85, s	14	59.5, CH_3	3.87, s	58.7, CH_3	3.80, s
OCH_2O	101.3, CH_2	5.97, m	12, 13<br>12, 13	101.2, CH_2	5.95, m	101.0, CH_2	6.05, d (1.0)<br>6.06, d (1.0)

[a]Data recorded in $CDCl_3$. [b]Data recorded in DMSO-d_6. [c]150 MHz. [d]500 MHz. [e]HMBC correlations are from proton(s) stated to the indicated carbon. [f]Assignment unconfirmed due to overlapping methyl peaks and broad carbon resonance. n.o.: not observed.

Table S2. NMR Data for Kadheterin K (**3**) in DMSO-d_6 and $CDCl_3$

Pos.	Kadheterin K (**3**) (DMSO-d_6)		Kadheterin K (**3**) ($CDCl_3$)		
	323 K		323 K		298 K
	δ_C^a, type	δ_H^b, mult (J in Hz)	δ_C^a, type	δ_H^b, mult (J in Hz)	δ_H^c, mult (J in Hz)
1	150.6, C	-	151.7, C	-	-
2	140.0, C	-	141.1, C	-	-
3	151.3, C	-	152.5, C	-	-
4	111.3, CH	6.77, s	110.9, CH	6.66, s	6.66, s
5	134.4, C	-	133.3, C	-	-
6	57.9, CH	4.67, dd (9.0, 9.0)	59.3, CH	5.06, brs	5.04, brs
7	37.2, CH	1.71, m	40.1, CH	1.88, m	1.86, m
8	39.4, CH	2.14, m	39.7, CH	2.09, m	2.08, m
9	78.1, CH	5.68, d (6.0)	80.2, CH	5.73, brs	5.71, d (3.4)
10	132.6, C	-	134.1, C	-	-
11	102.1, CH	6.60, s	103.0, CH	6.50, s	6.50, s
12	148.1, C	-	149.6, C	-	-
13	135.5, C	-	136.2, C	-	-
14	141.5, C	-	141.8, C	-	-
15	120.5, C	-	120.6, C	-	-
16	121.9, C	-	122.0, C	-	-
17	17.9,[f] CH_3	0.86, d (6.0)	16.9, C	0.96, brs	0.95, d (6.5)
18	17.9,[f] CH_3	0.84, d (6.0)	n.o., CH_3	1.02, brs	1.01, brd (6.5)
OCH_3-1	59.4, CH_3	3.47, s	60.3, CH_3	3.61, s	3.61, s
OCH_3-2	59.8, CH_3	3.79, s	60.8, CH_3	3.88, s	3.86, s
OCH_3-3	55.8, CH_3	3.75, s	56.4, CH_3	3.89, s	3.89, s
NH-6	-	n.o.	-	5.29, brs	5.22, d (9.5)
*Me*CO-6	22.4, CH_3	1.63, brs	23.9, CH_3	1.76, brs	1.73, brs
Me*CO*-6	167.6, C	-	n.o.	-	-
*Me*CO-9	20.0, CH_3	1.48, s	20.7, CH_3	1.60, s	1.60, s
Me*CO*-9	169.0, C	-	170.1, C	-	-
OCH_3-14	58.7, CH_3	3.80, s	59.7, CH_3	3.91, s	3.91, s
OCH_2O	101.0, CH_2	6.05, d (1.0)<br>6.06, d (1.0)	101.6, CH_2	6.01, brs	6.02, d (1.0)<br>6.01, d (1.0)

[a]150 MHz. [b]500 MHz. [c]600 MHz. [d]Assignment unconfirmed due to overlapping methyl peaks and broad carbon resonance. n.o.: not observed.

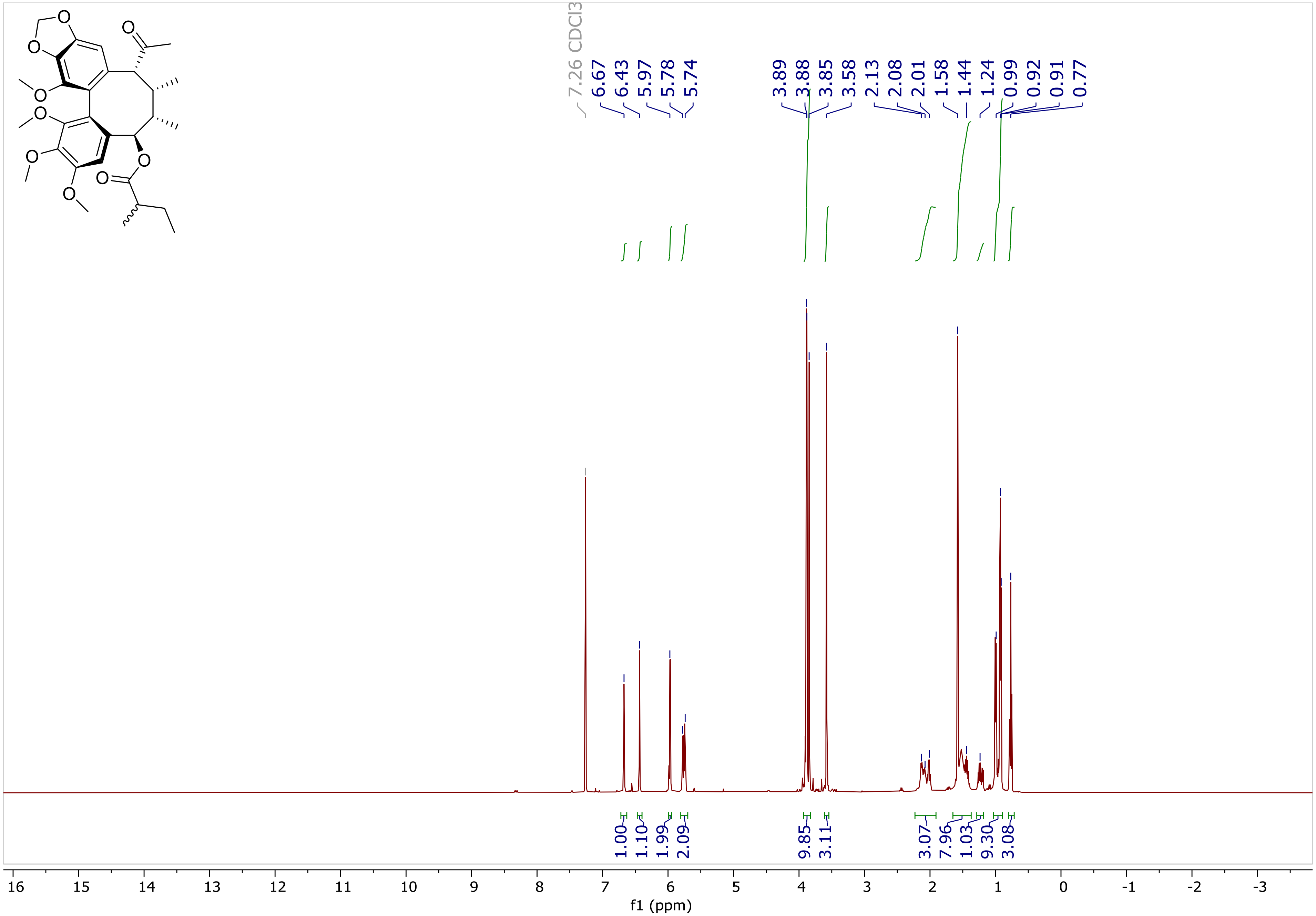

Figure S2. ^{1}H NMR spectrum of **1** in chloroform-*d* at 323K (500 MHz)

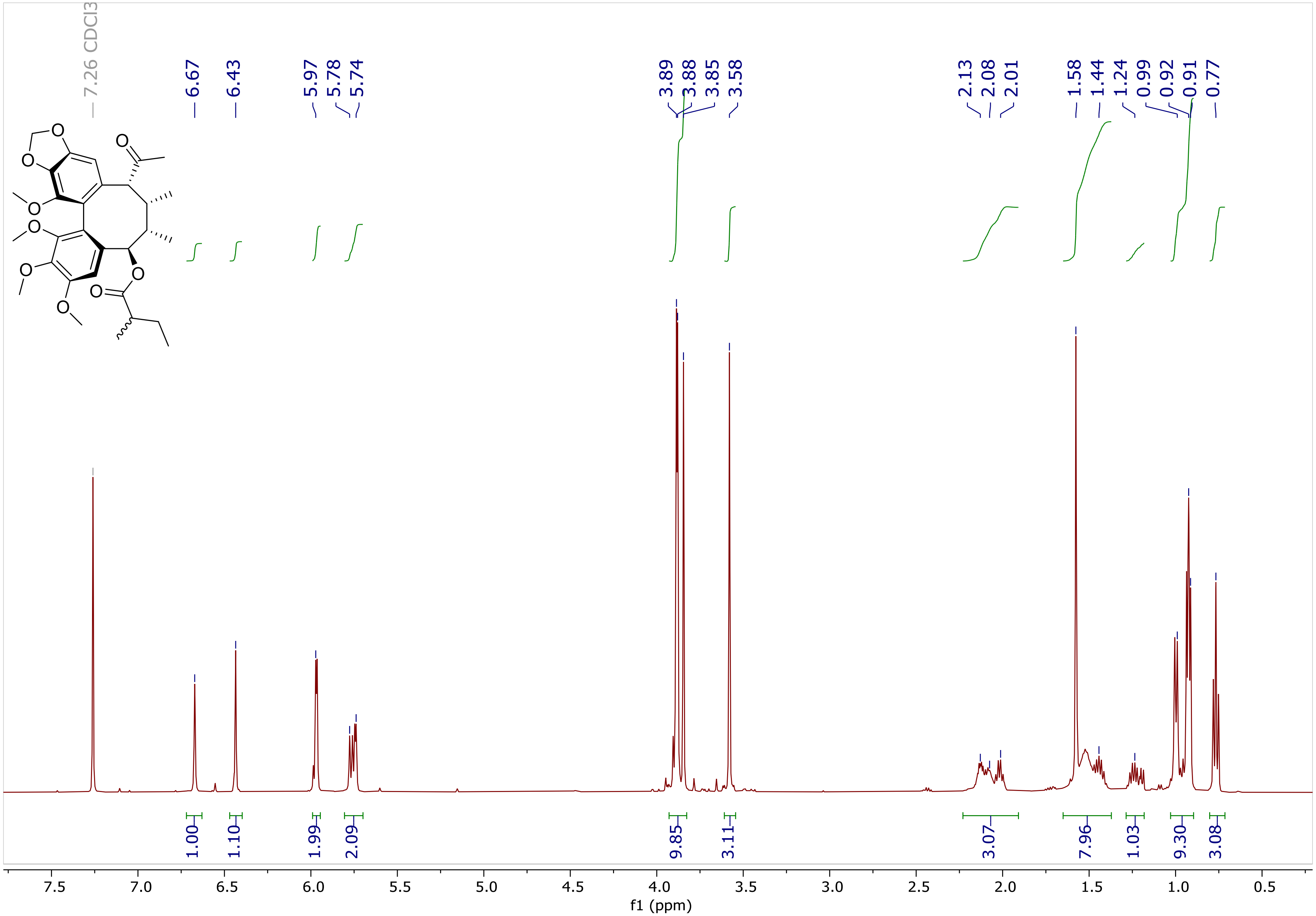

Figure S3. Zoomed ^{1}H NMR spectrum of **1** in chloroform-*d* at 323K (500 MHz)

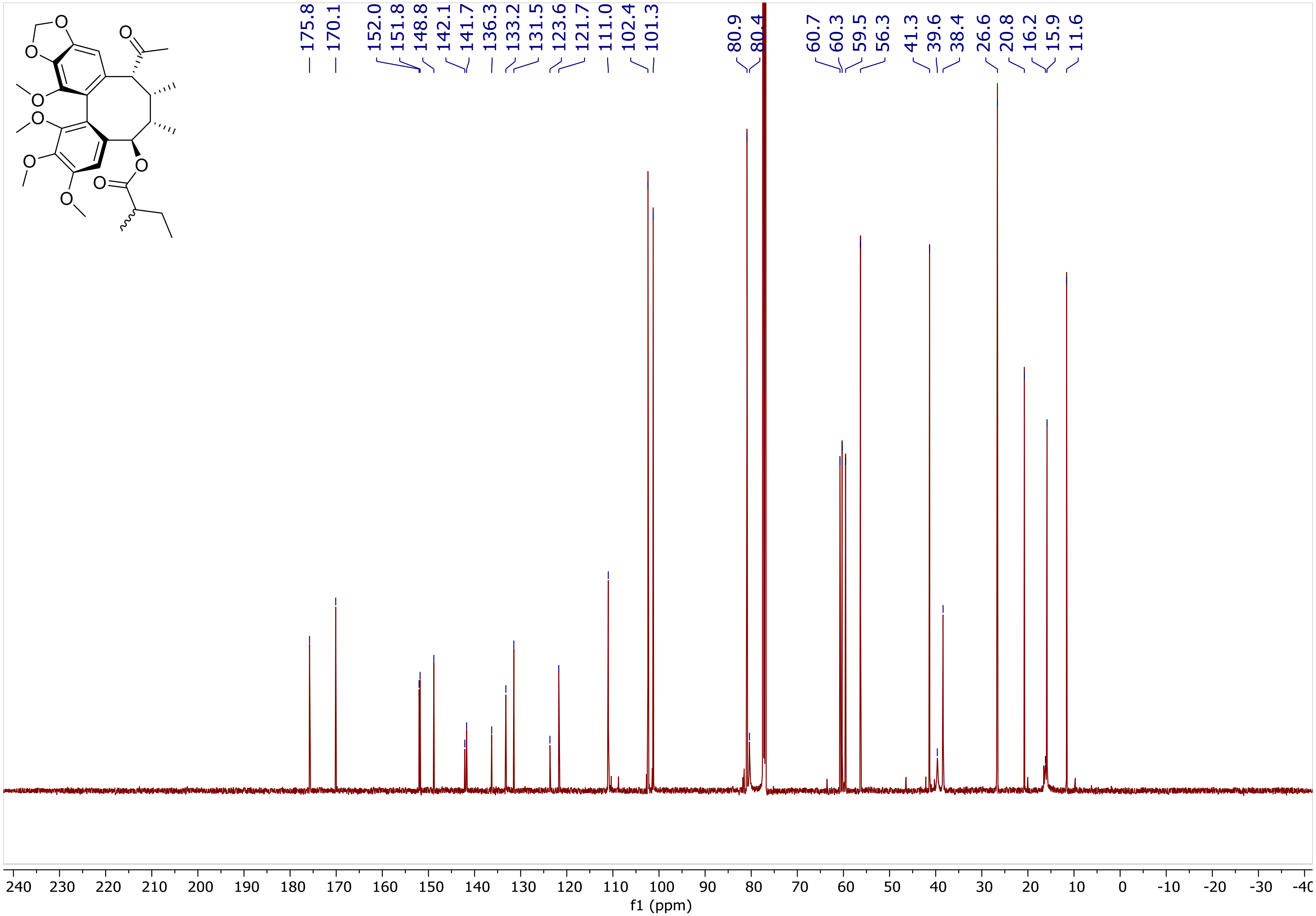

Figure S4. ^{13}C NMR spectrum of **1** in chloroform-*d* at 323K (125 MHz)

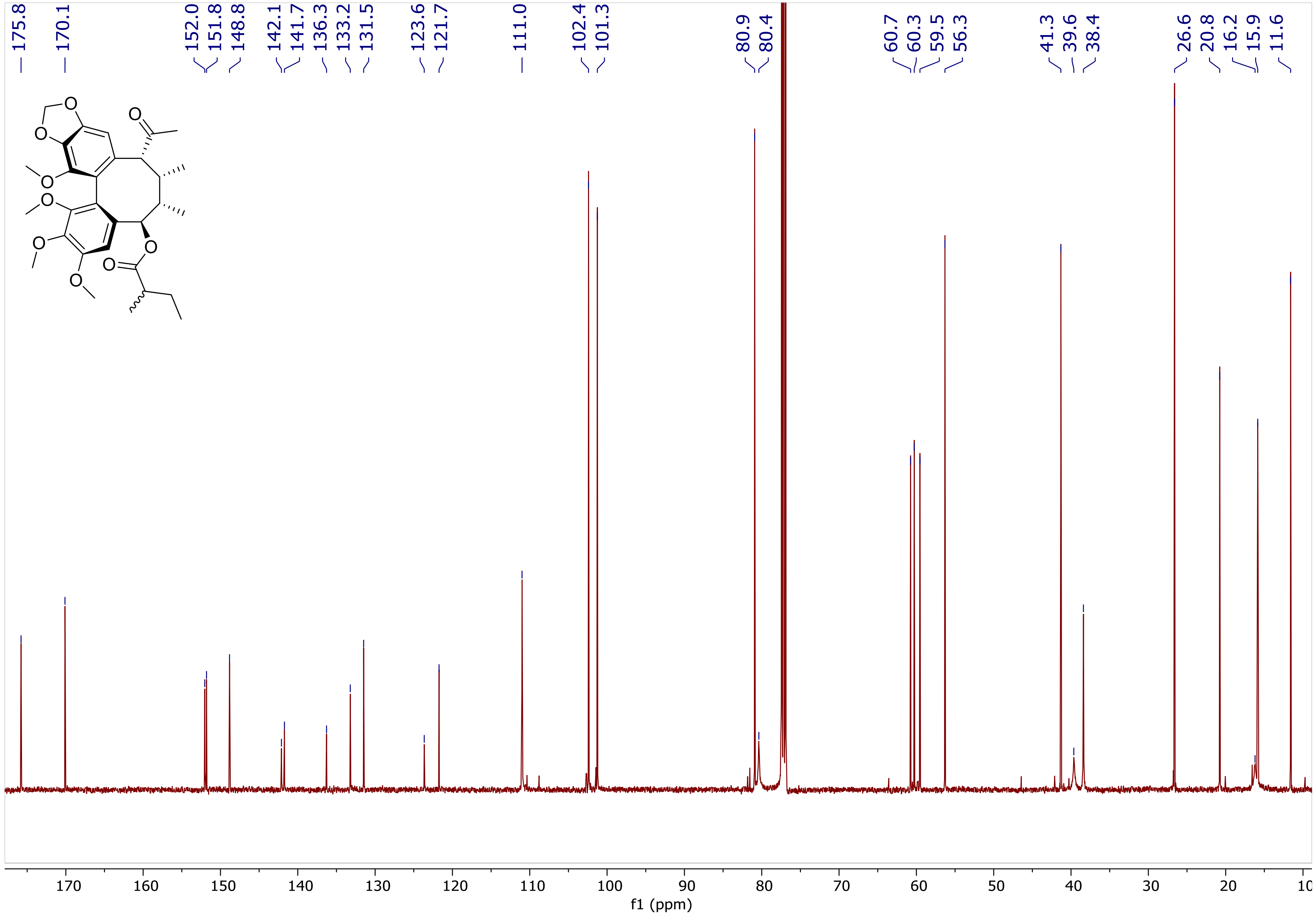

Figure S5. Zoomed ^{13}C NMR spectrum of **1** in chloroform-*d* at 323K (125 MHz)

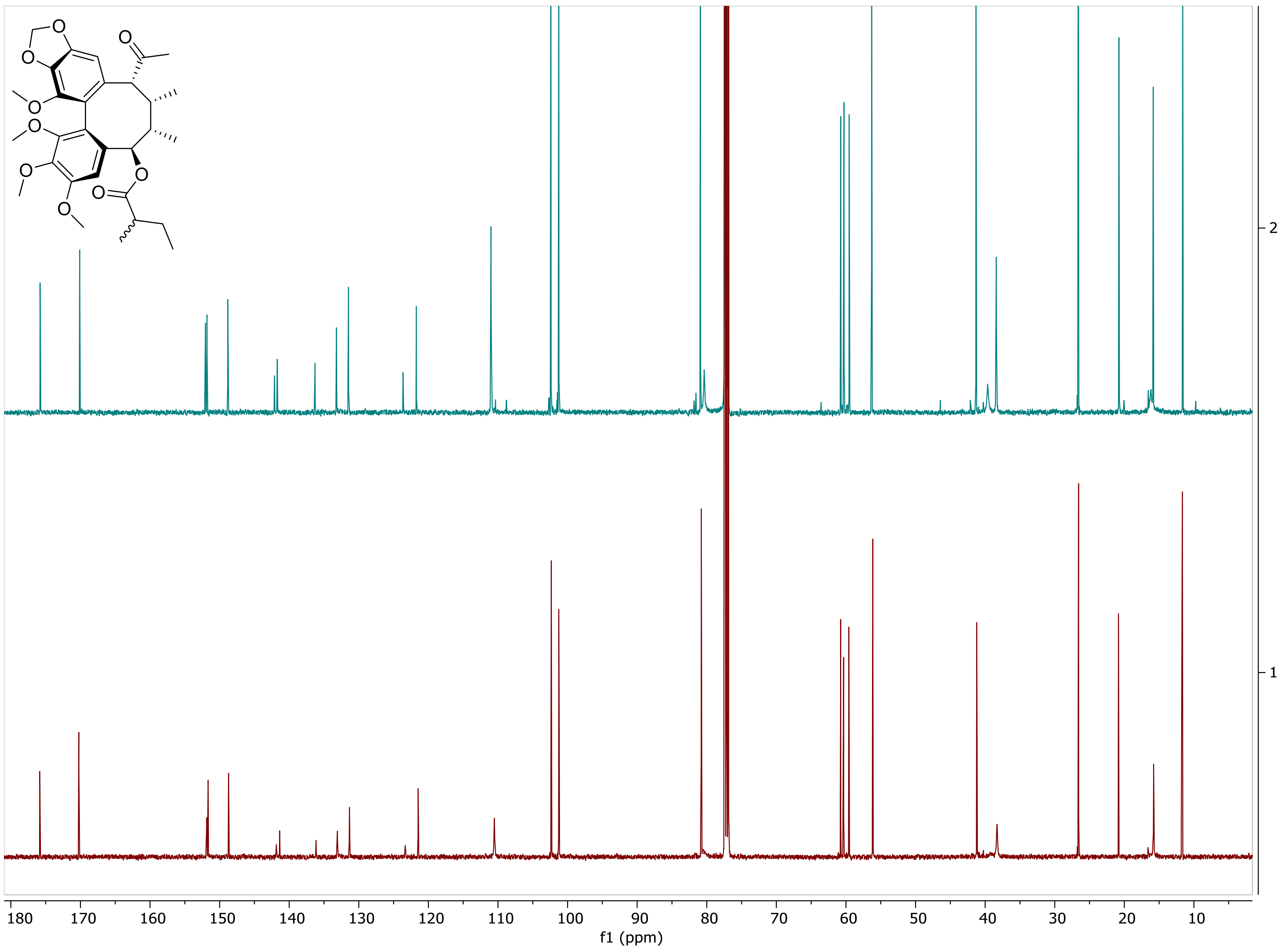

Figure S6. ^{13}C NMR spectra of **1** in chloroform-*d* at 323K (top) and 298K (bottom) (125 MHz)

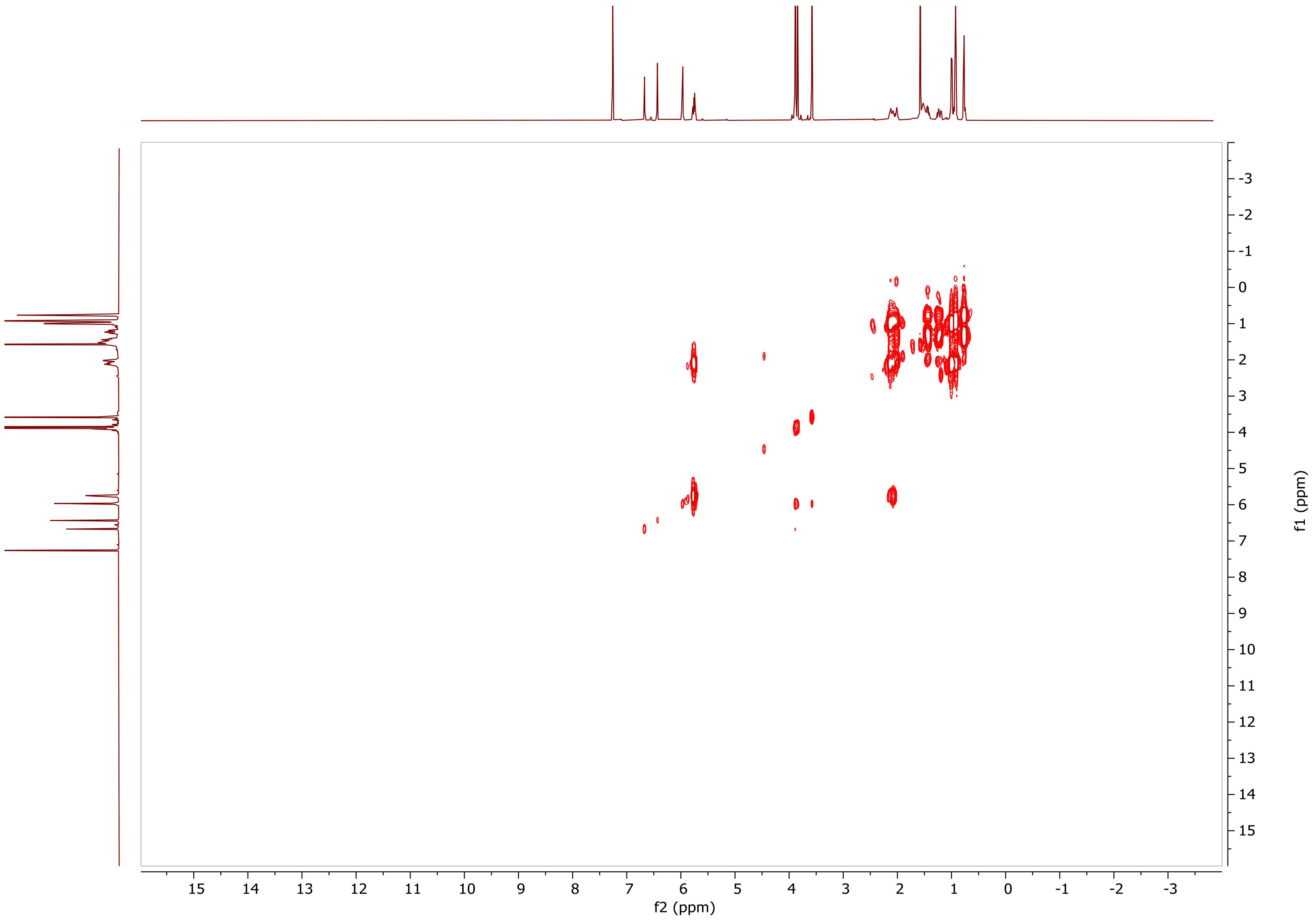

Figure S7. COSY NMR spectrum of **1** in chloroform-*d* at 323K (500 MHz)

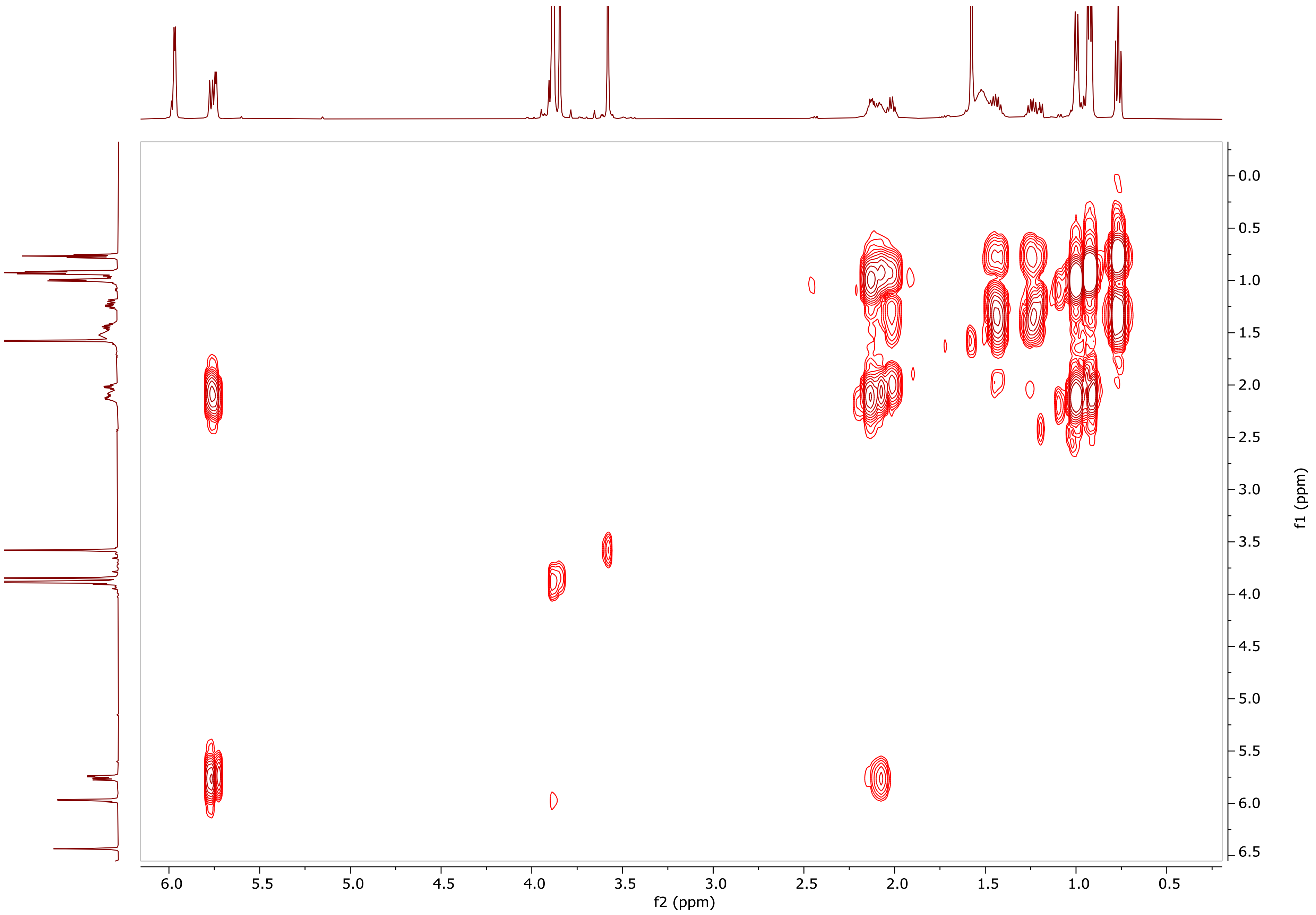

Figure S8. Zoomed COSY NMR spectrum of **1** in chloroform-*d* at 323K (500 MHz)

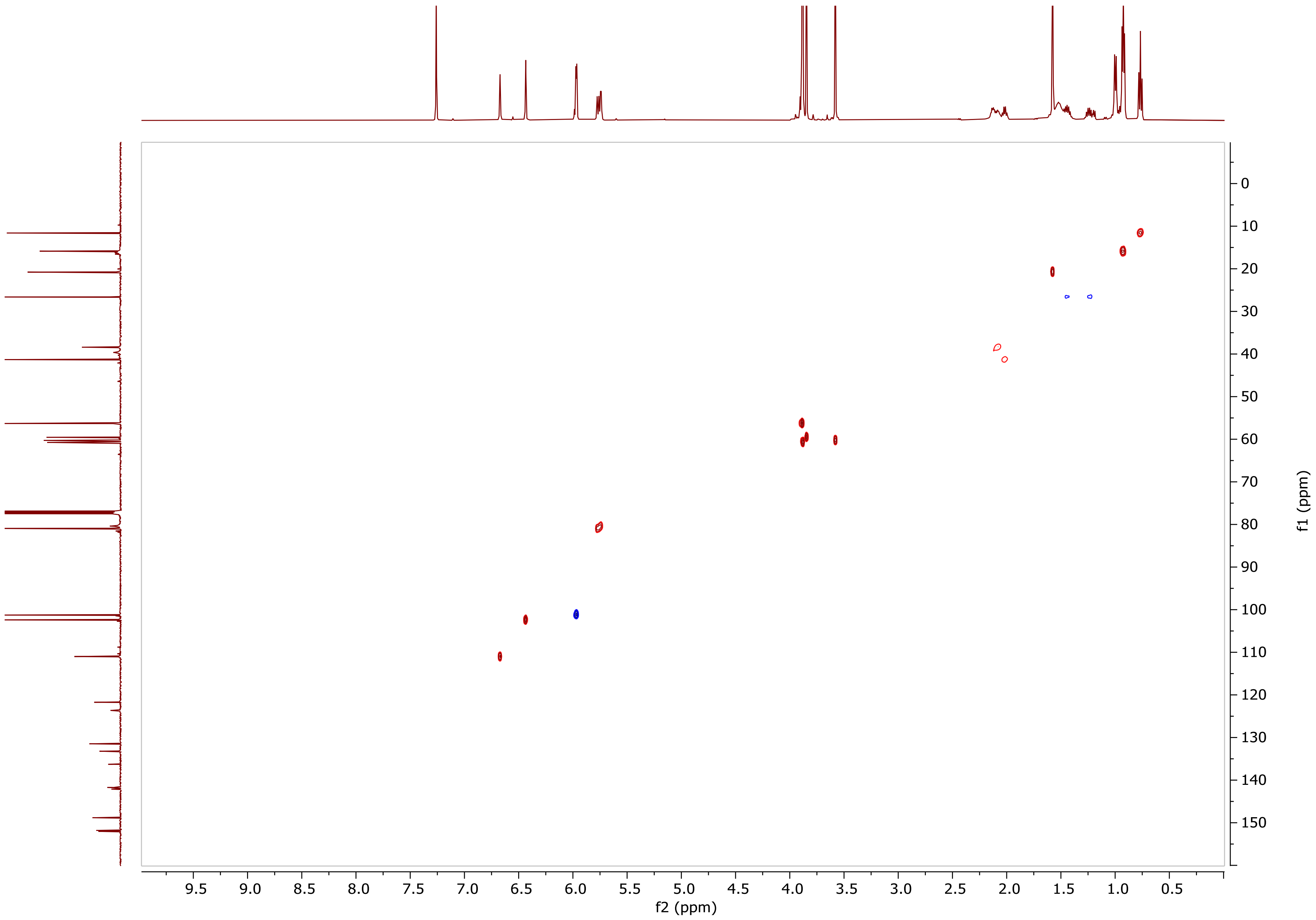

Figure S9. HSQC NMR spectrum of **1** in chloroform-*d* at 323K (500 MHz)

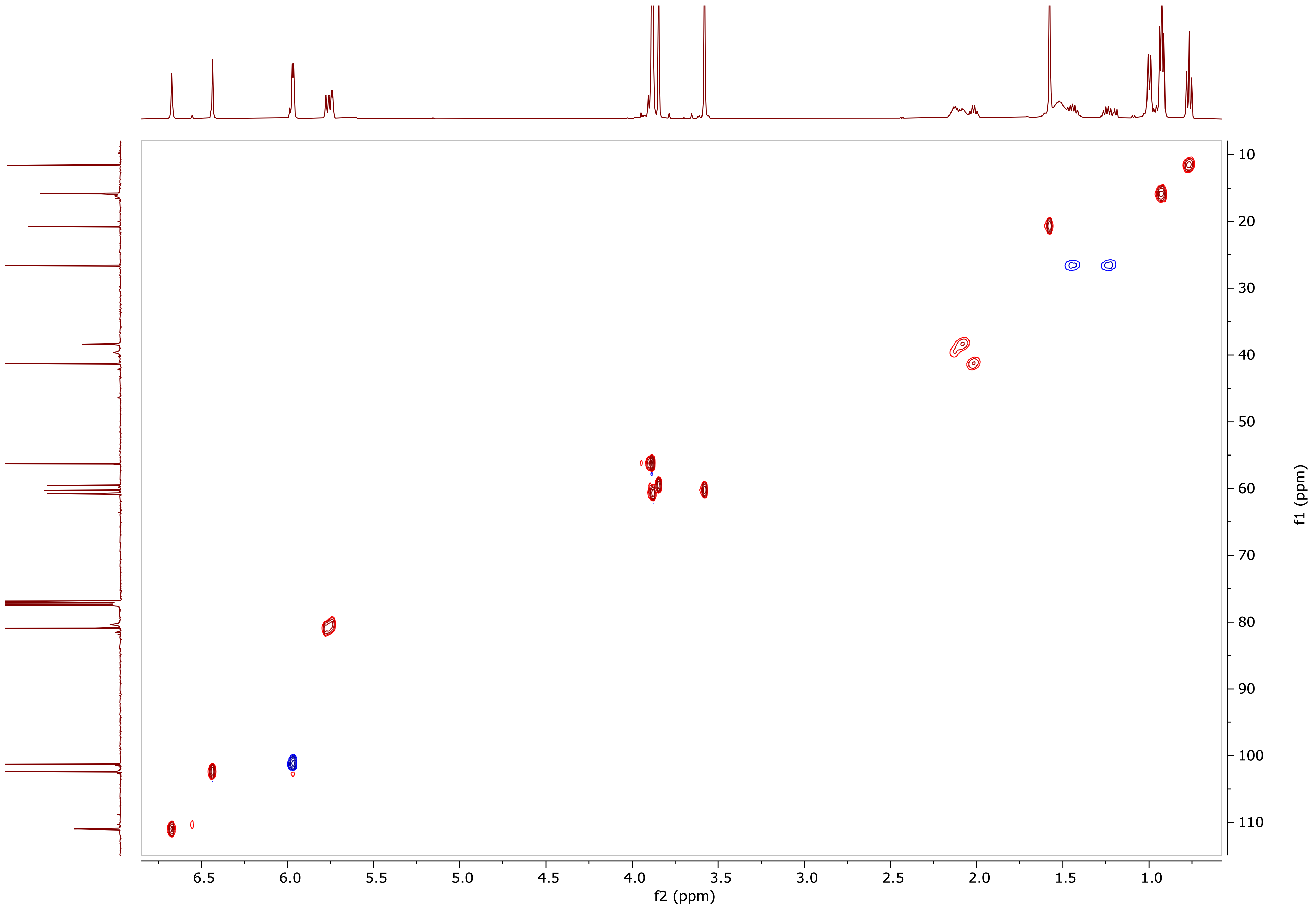

Figure S10. Zoomed HSQC NMR spectrum of **1** in chloroform-*d* at 323K (500 MHz)

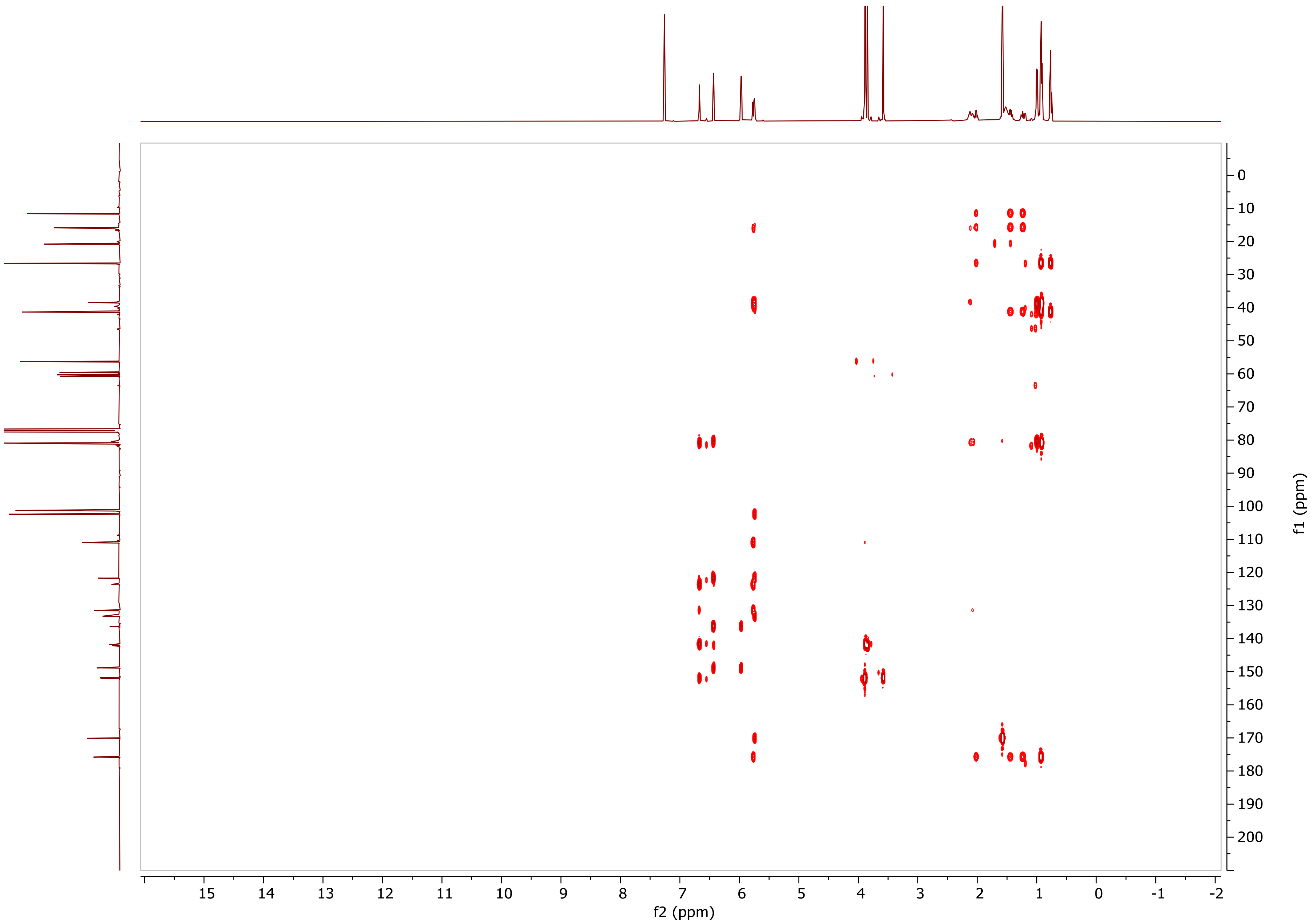

Figure S11. HMBC NMR spectrum of **1** in chloroform-*d* at 323K (500 MHz)

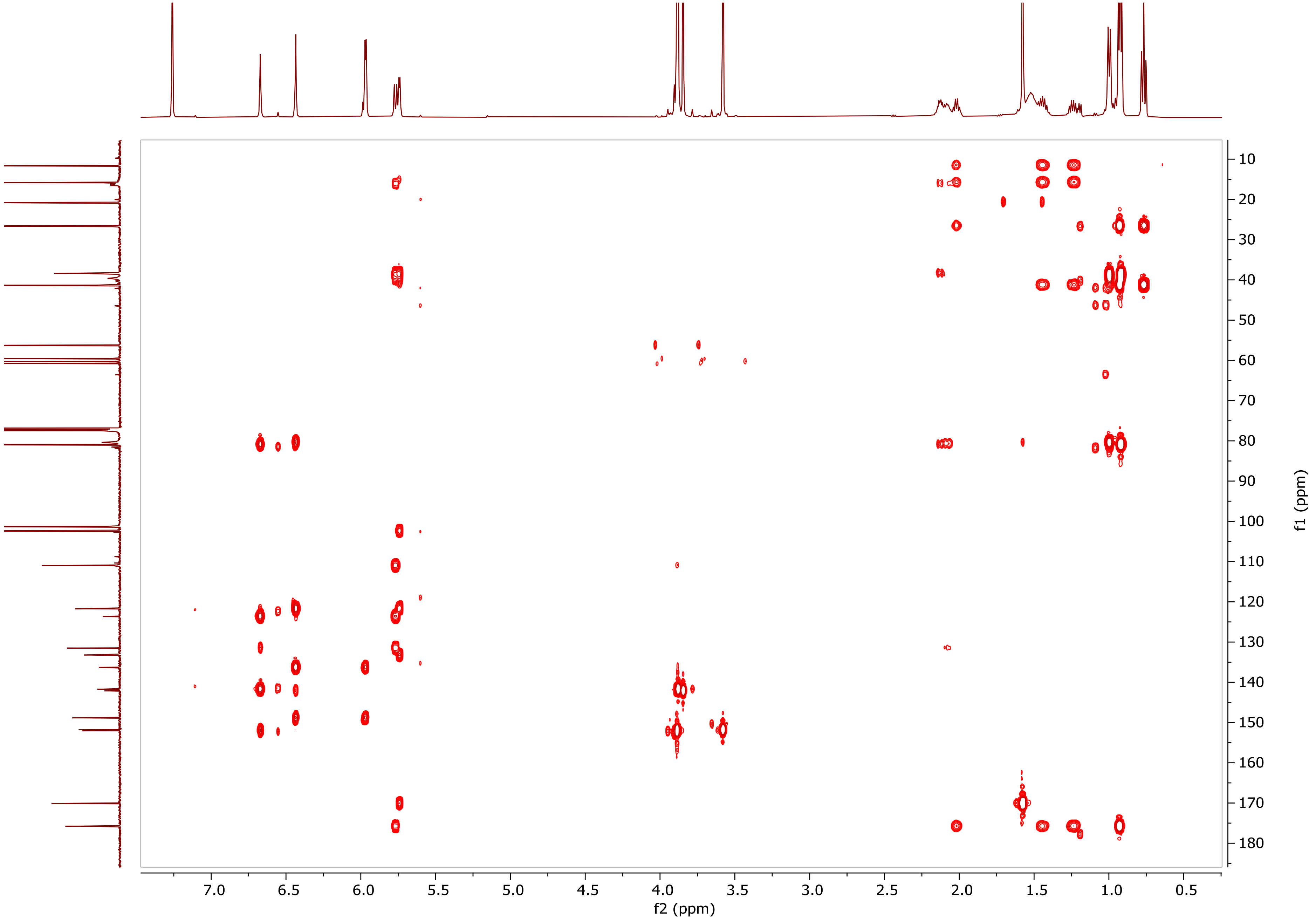

Figure S12. Zoomed HMBC NMR spectrum of **1** in chloroform-*d* at 323K (500 MHz)

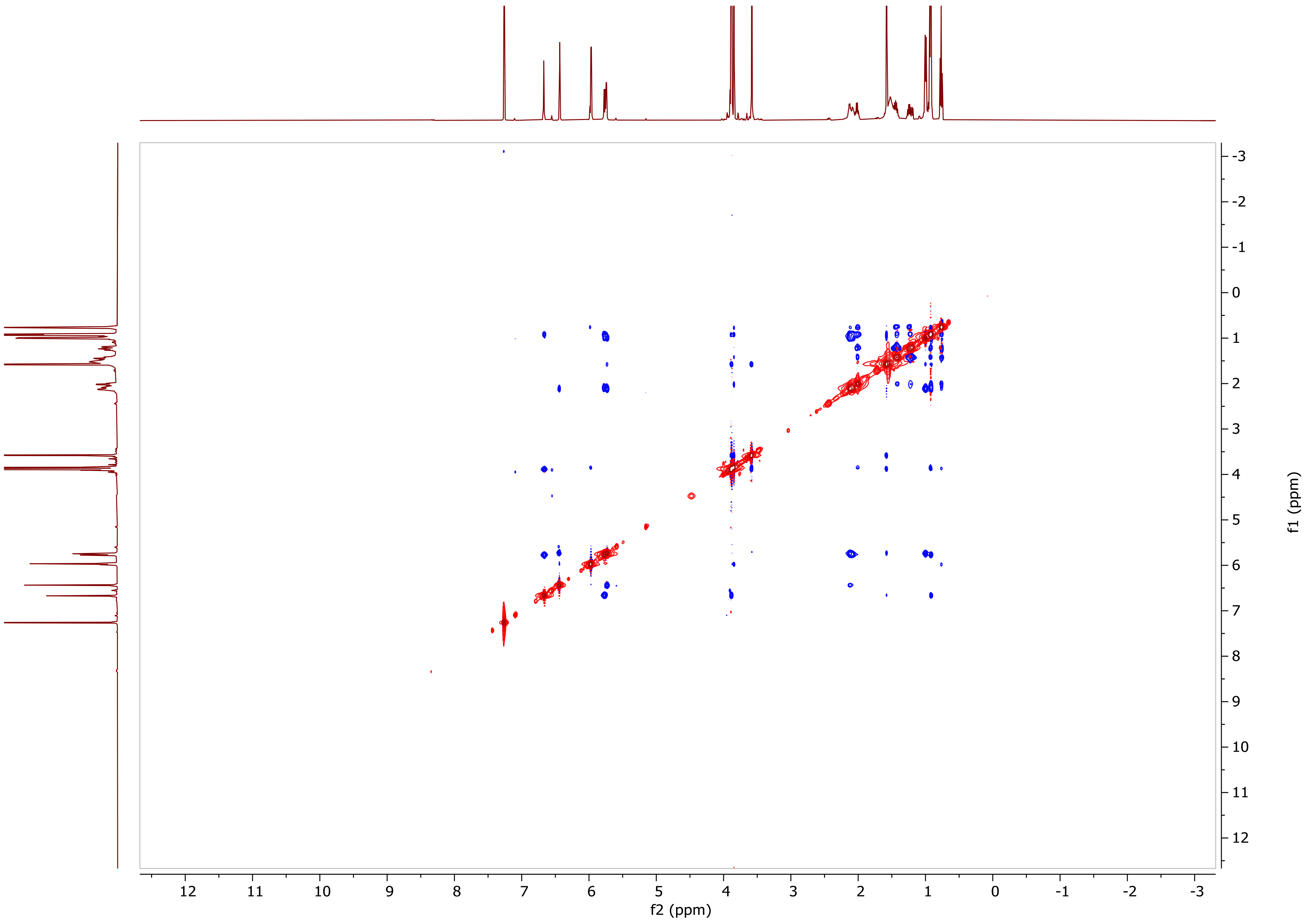

Figure S13. NOESY NMR spectrum of **1** in chloroform-*d* at 323K (500 MHz)

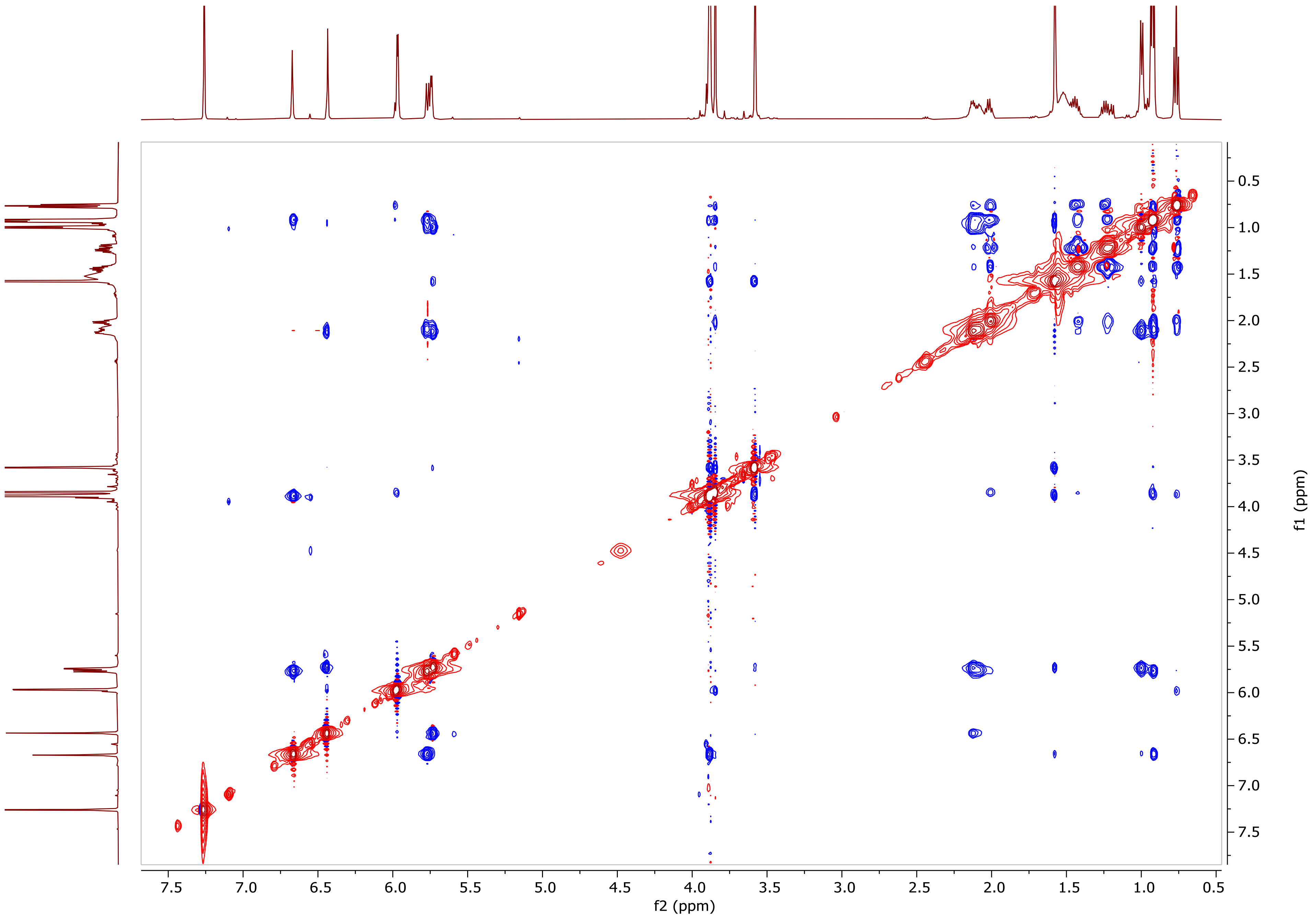

Figure S14. Zoomed NOESY NMR spectrum of **1** in chloroform-*d* at 323K (500 MHz)

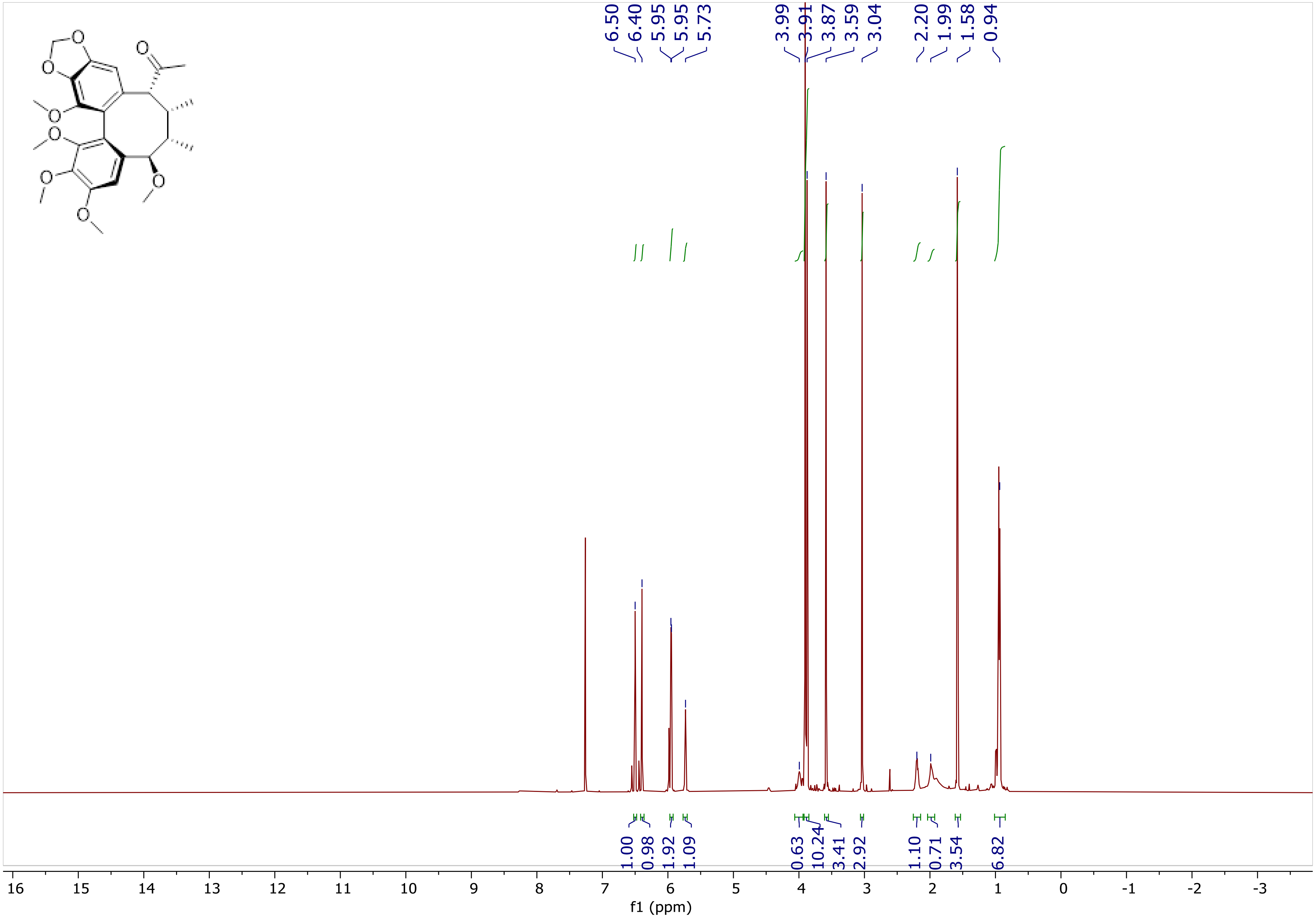

Figure S15. ^{1}H NMR spectrum of **2** in chloroform-*d* at 323K (500 MHz). The c.a. 10% impurity is likely the 6′-OH derivative of **2**.

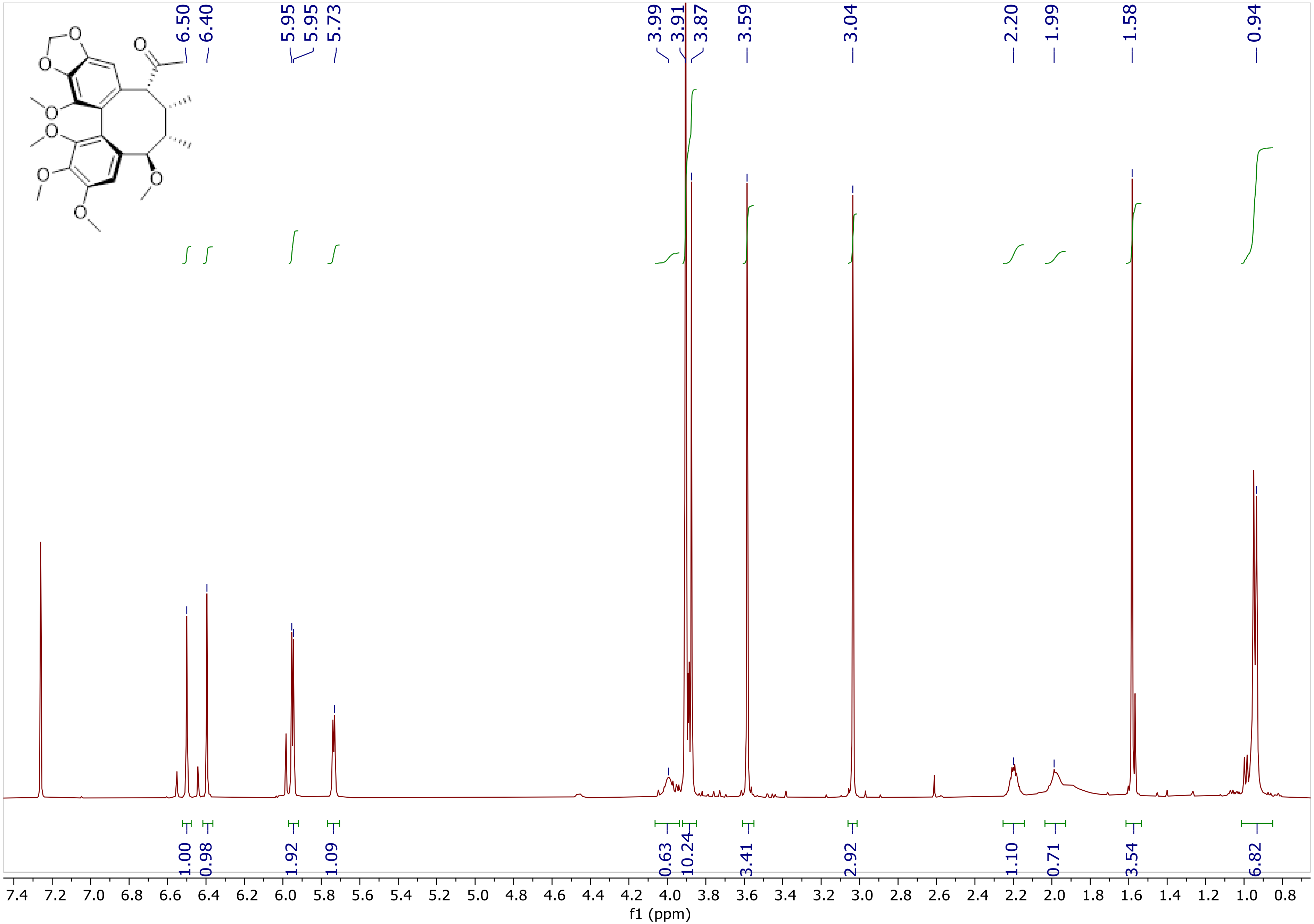

Figure S16. Zoomed ^{1}H NMR spectrum of **2** in chloroform-*d* at 323K (500 MHz). The c.a. 10% impurity is likely the 6′-OH derivative of **2**.

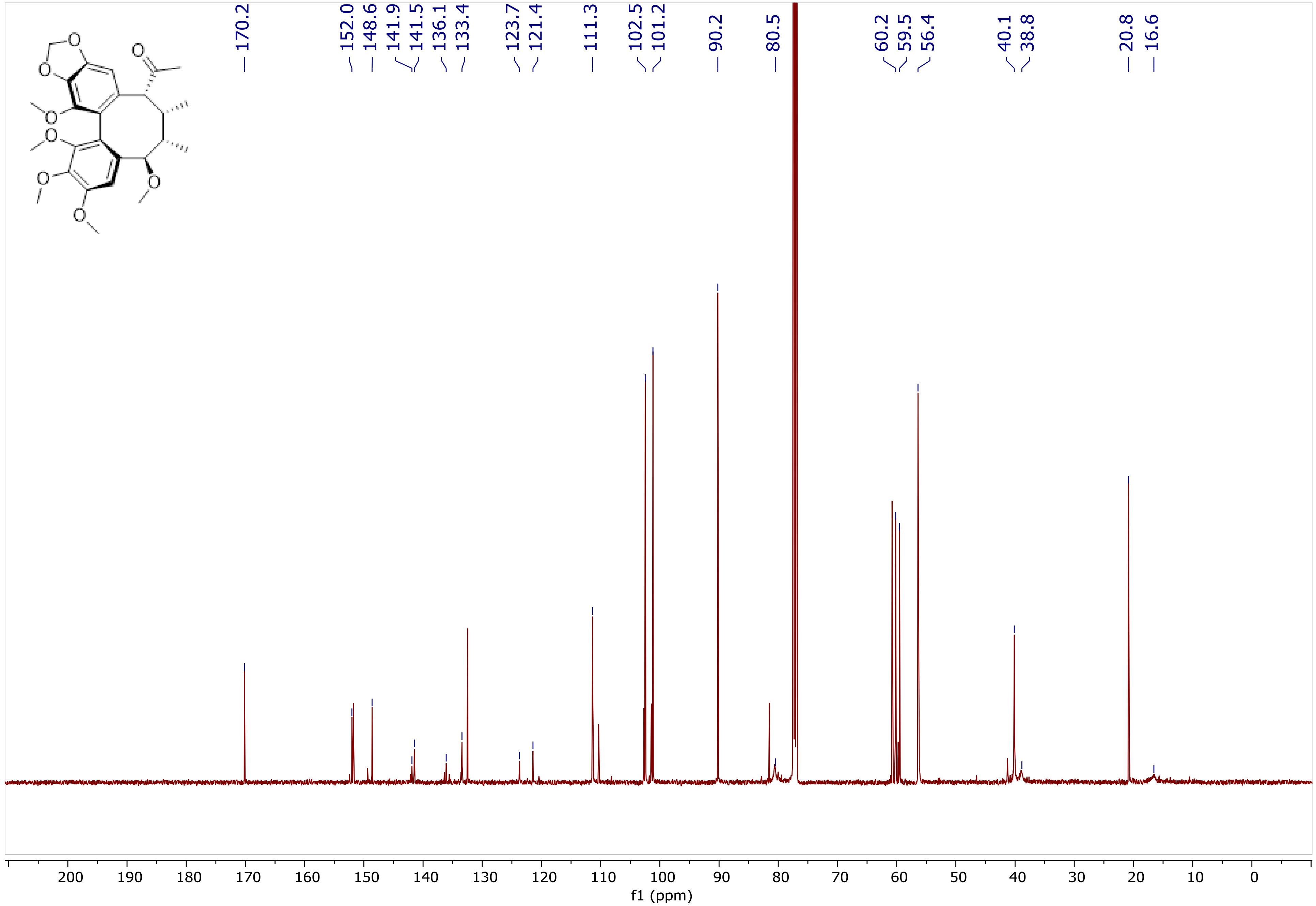

Figure S17. ^{13}C NMR spectrum of **2** in chloroform-*d* at 323K (500 MHz). The c.a. 10% impurity is likely the 6′-OH derivative of **2**.

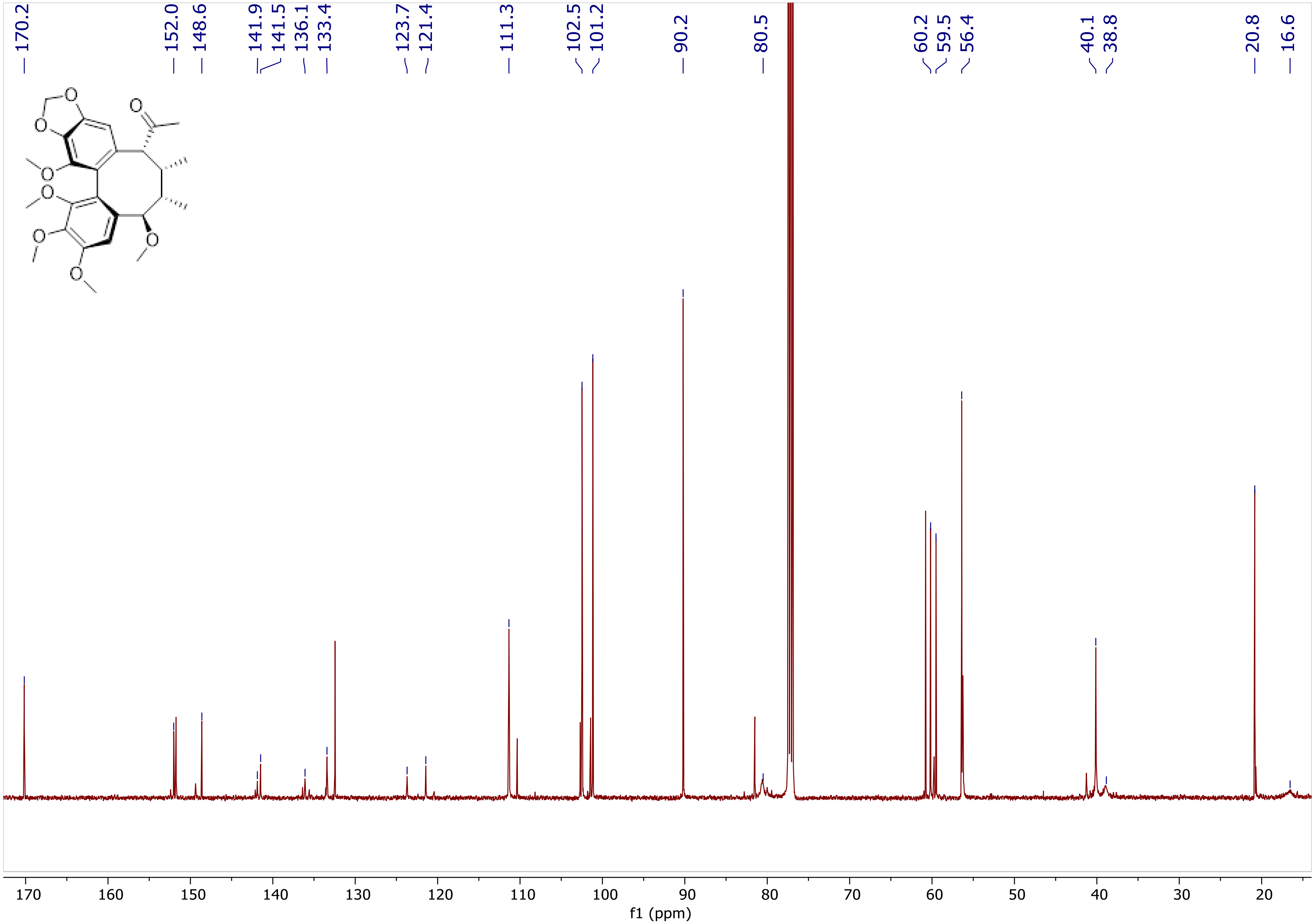

Figure S18. Zoomed ^{13}C NMR spectrum of **2** in chloroform-*d* at 323K (500 MHz). The c.a. 10% impurity is likely the 6′-OH derivative of **2**.

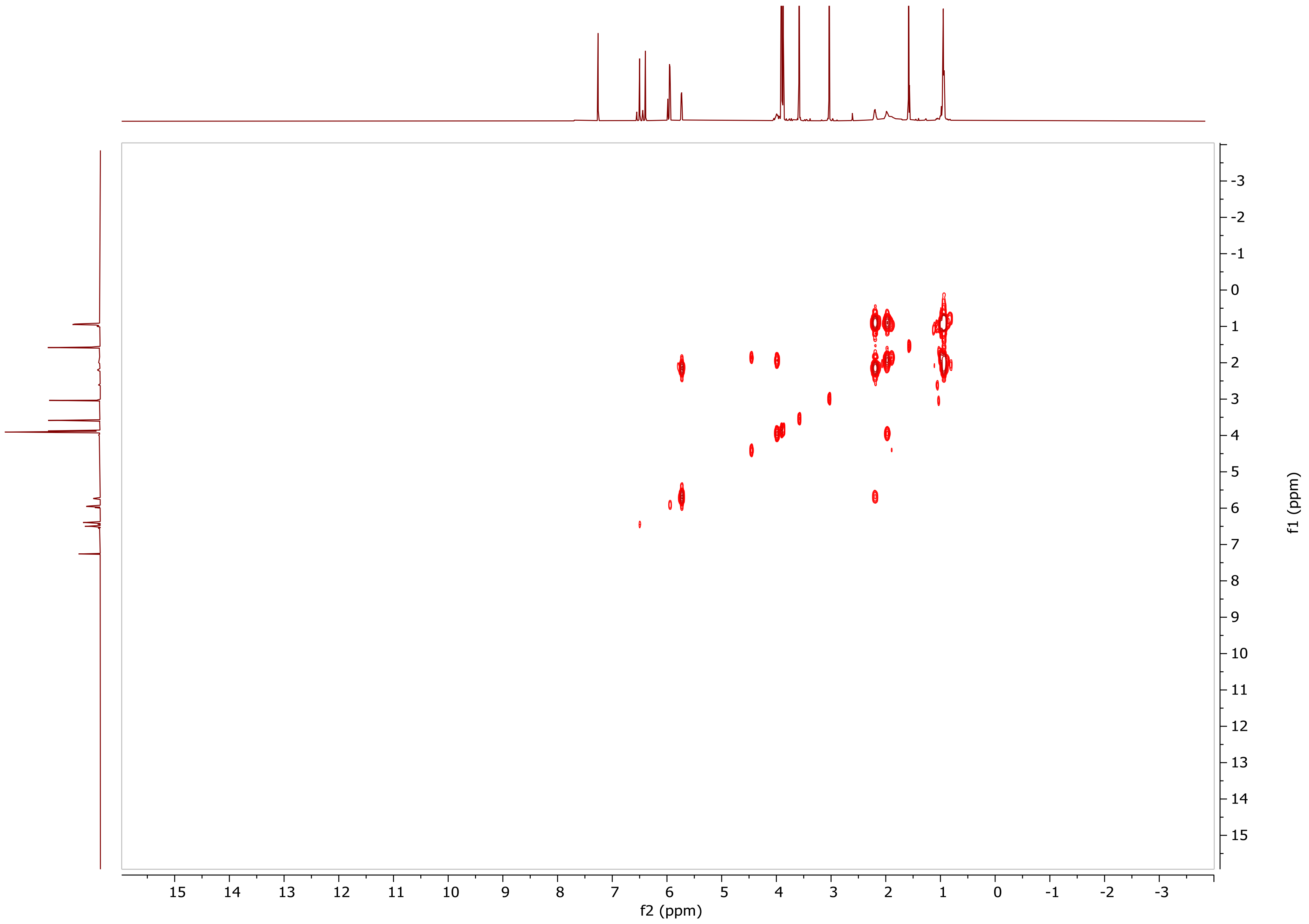

Figure S19. COSY NMR spectrum of **2** in chloroform-*d* at 323K (500 MHz)

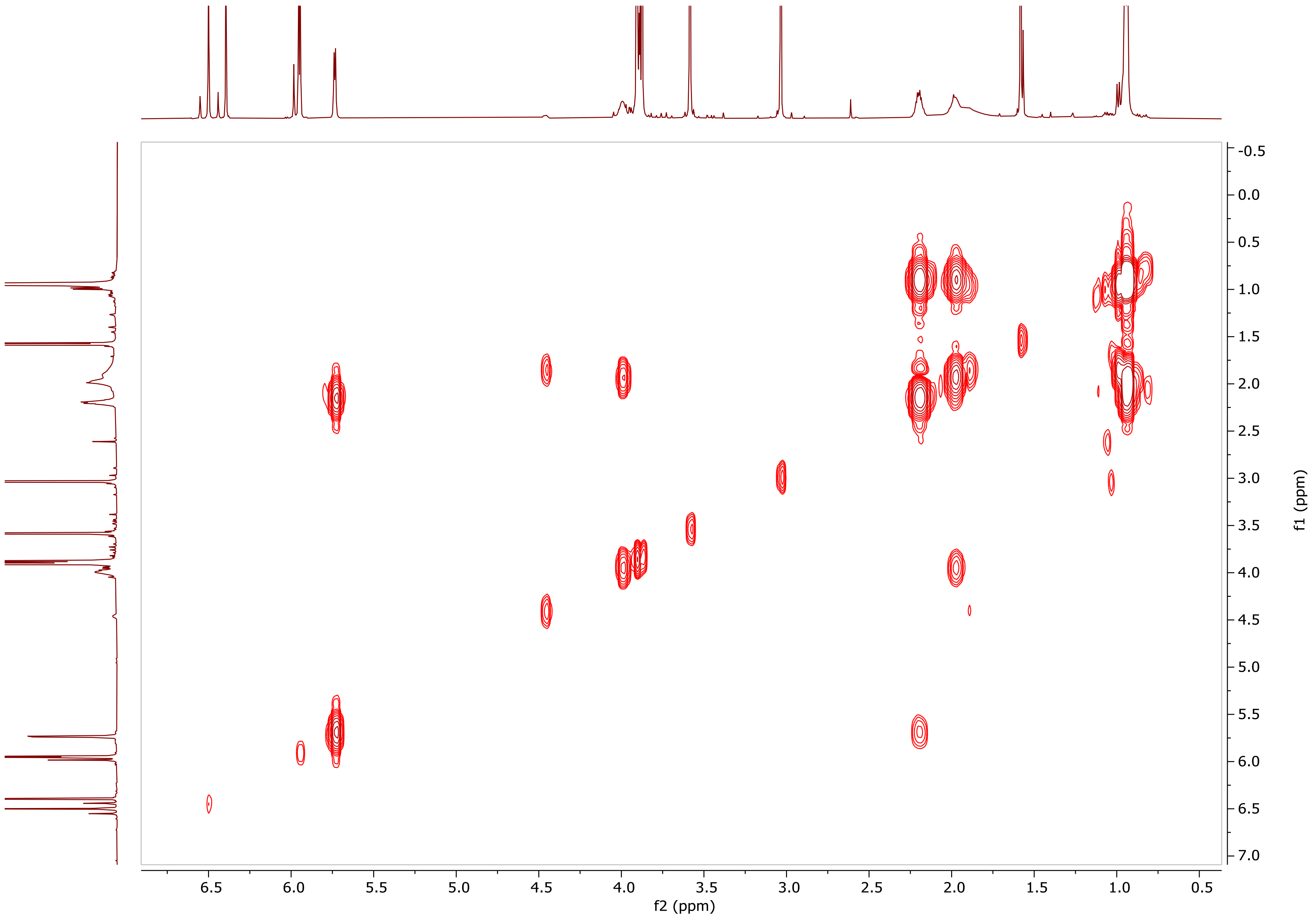

Figure S20. Zoomed COSY NMR spectrum of **2** in chloroform-*d* at 323K (500 MHz)

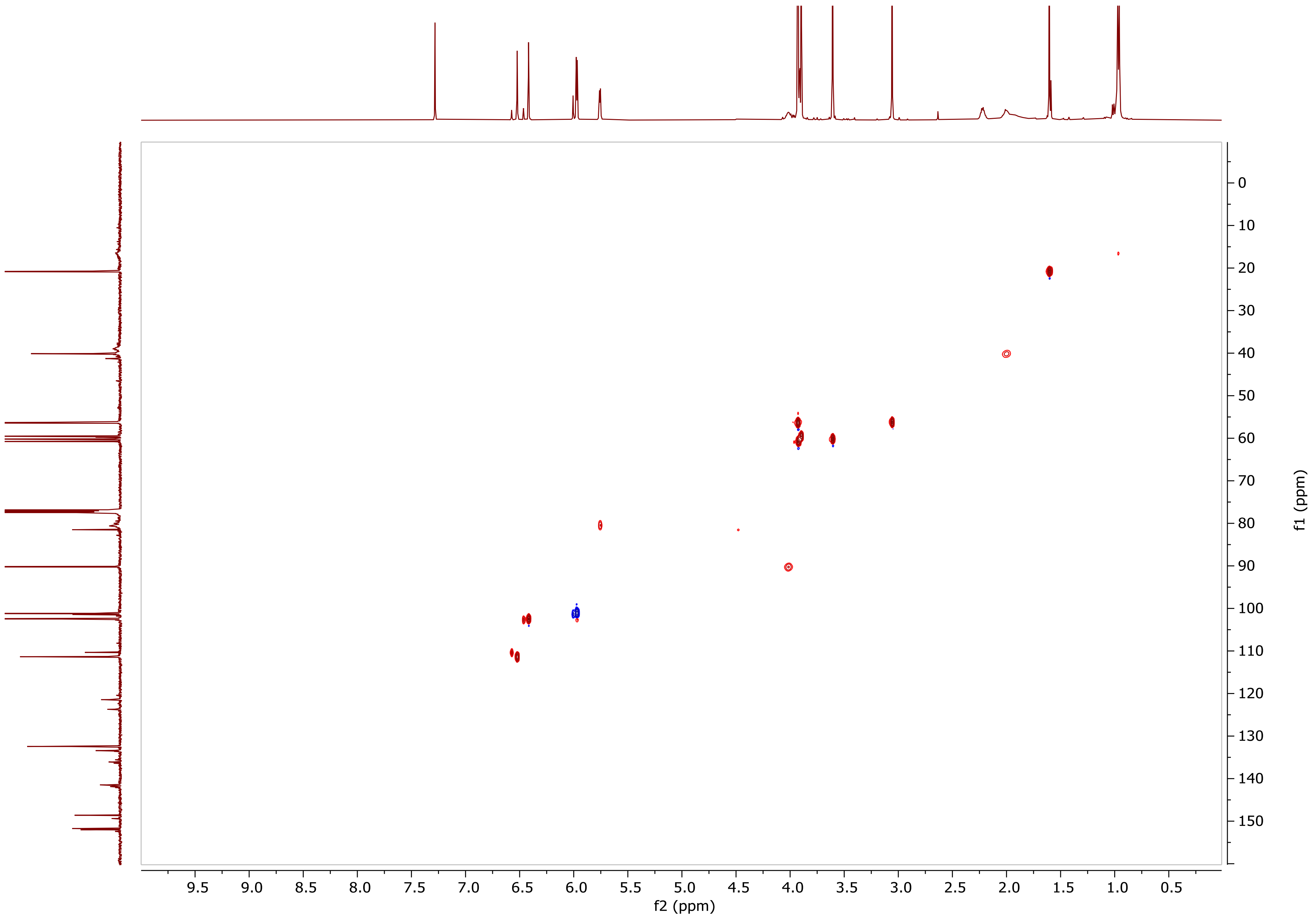

Figure S21. HSQC NMR spectrum of **2** in chloroform-*d* at 323K (500 MHz)

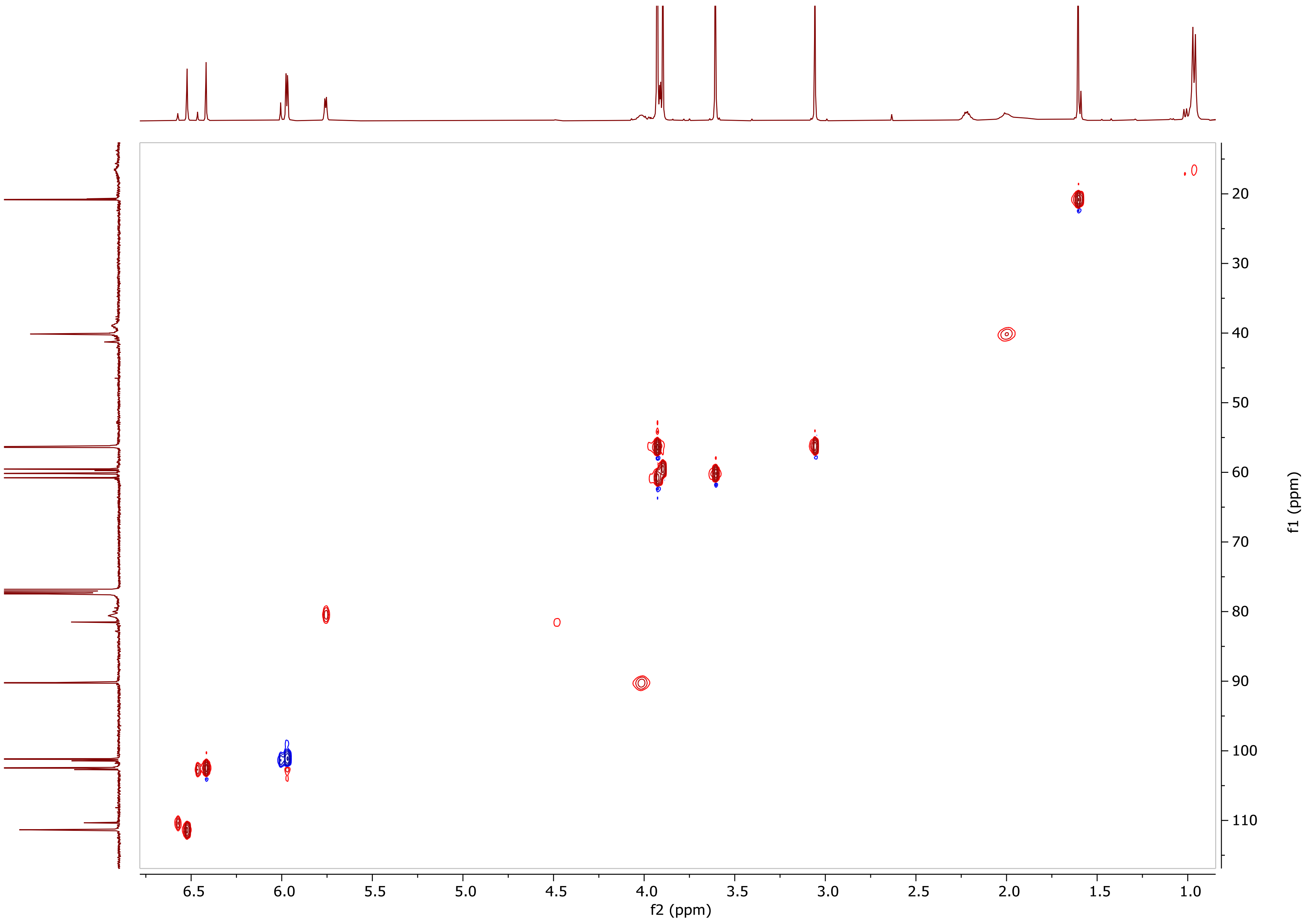

Figure S22. Zoomed HSQC NMR spectrum of **2** in chloroform-*d* at 323K (500 MHz)

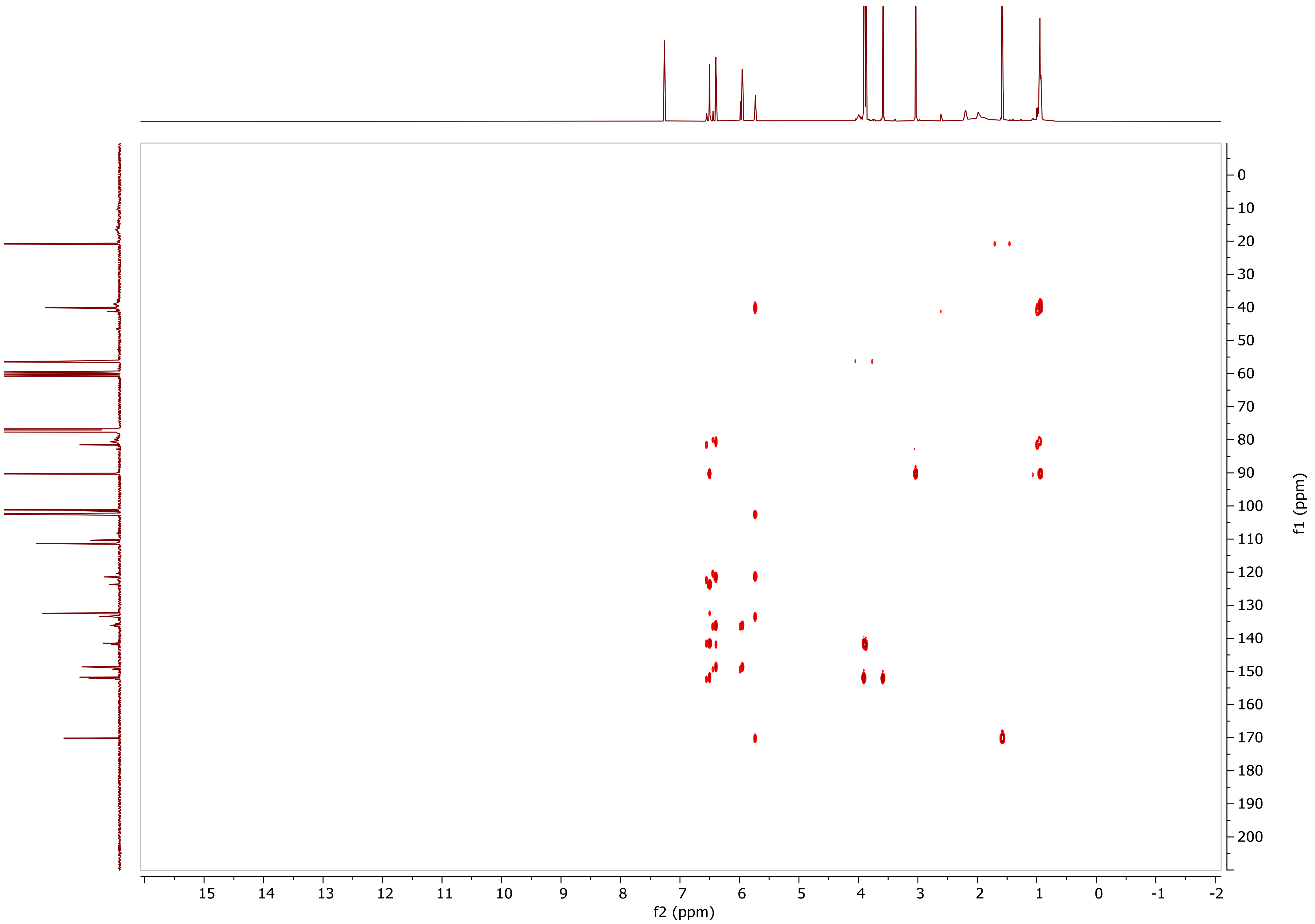

Figure S23. HMBC NMR spectrum of **2** in chloroform-*d* at 323K (500 MHz)

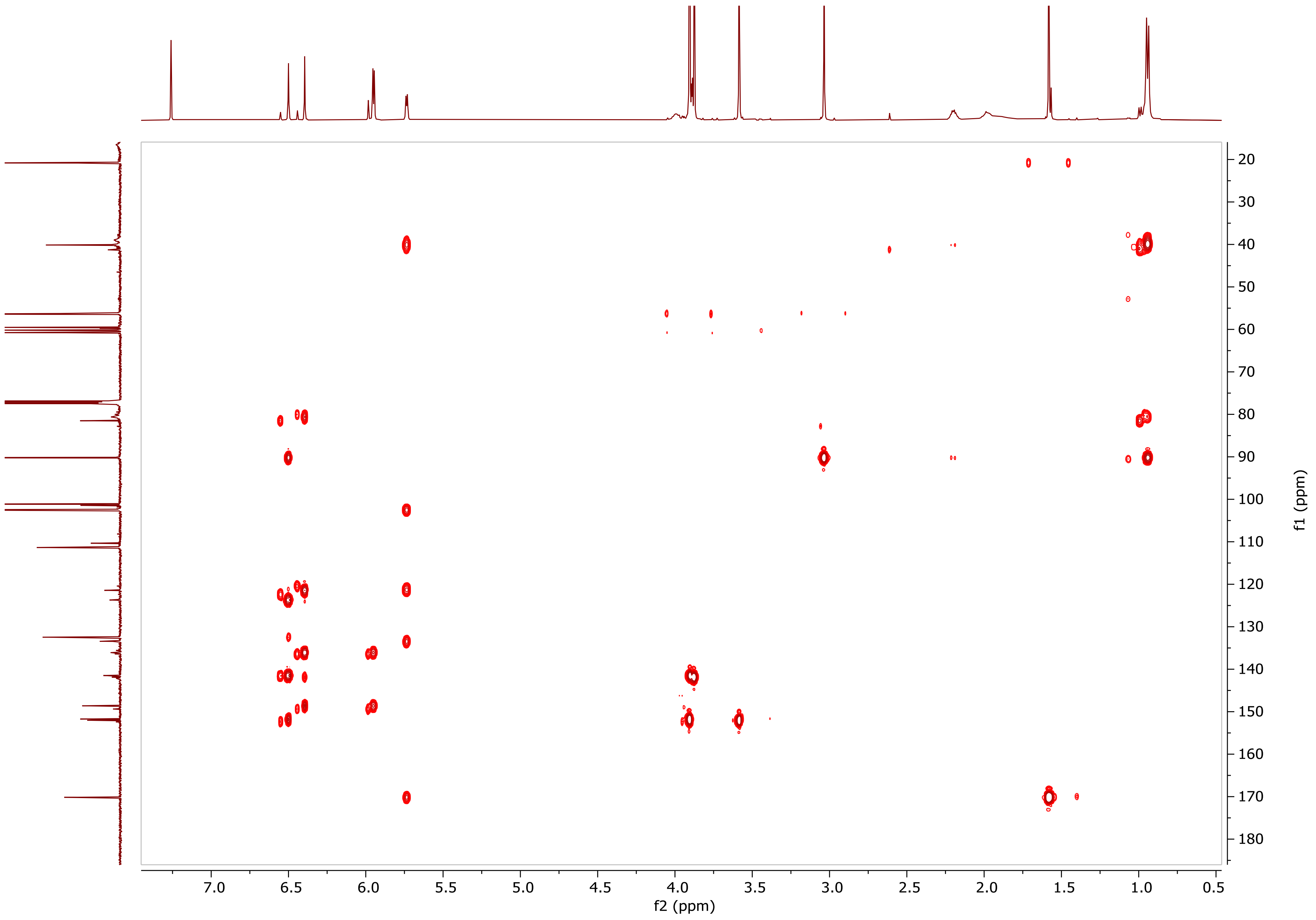

Figure S24. Zoomed HMBC NMR spectrum of **2** in chloroform-*d* at 323K (500 MHz)

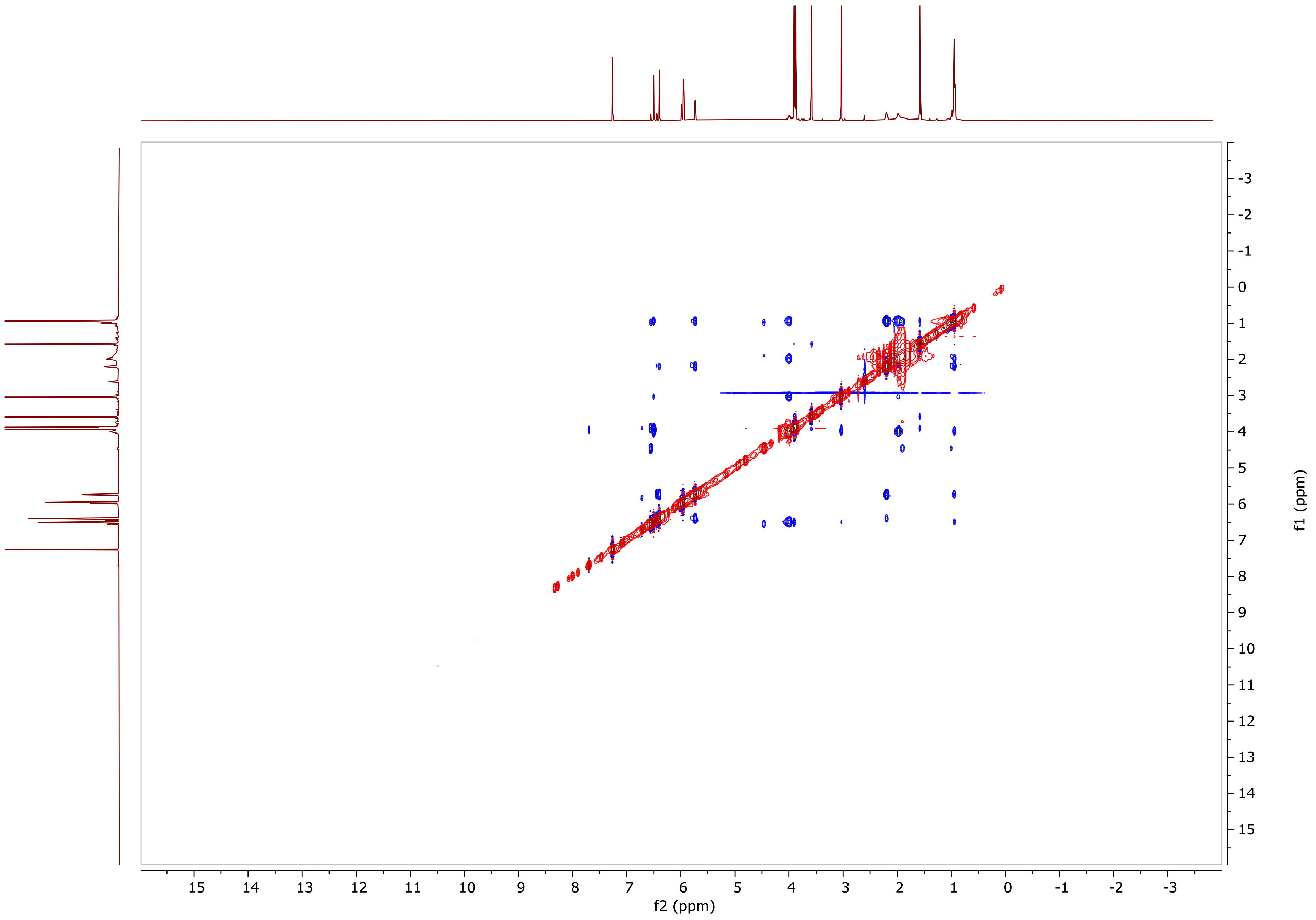

Figure S25. NOESY NMR spectrum of **2** in chloroform-*d* at 323K (500 MHz)

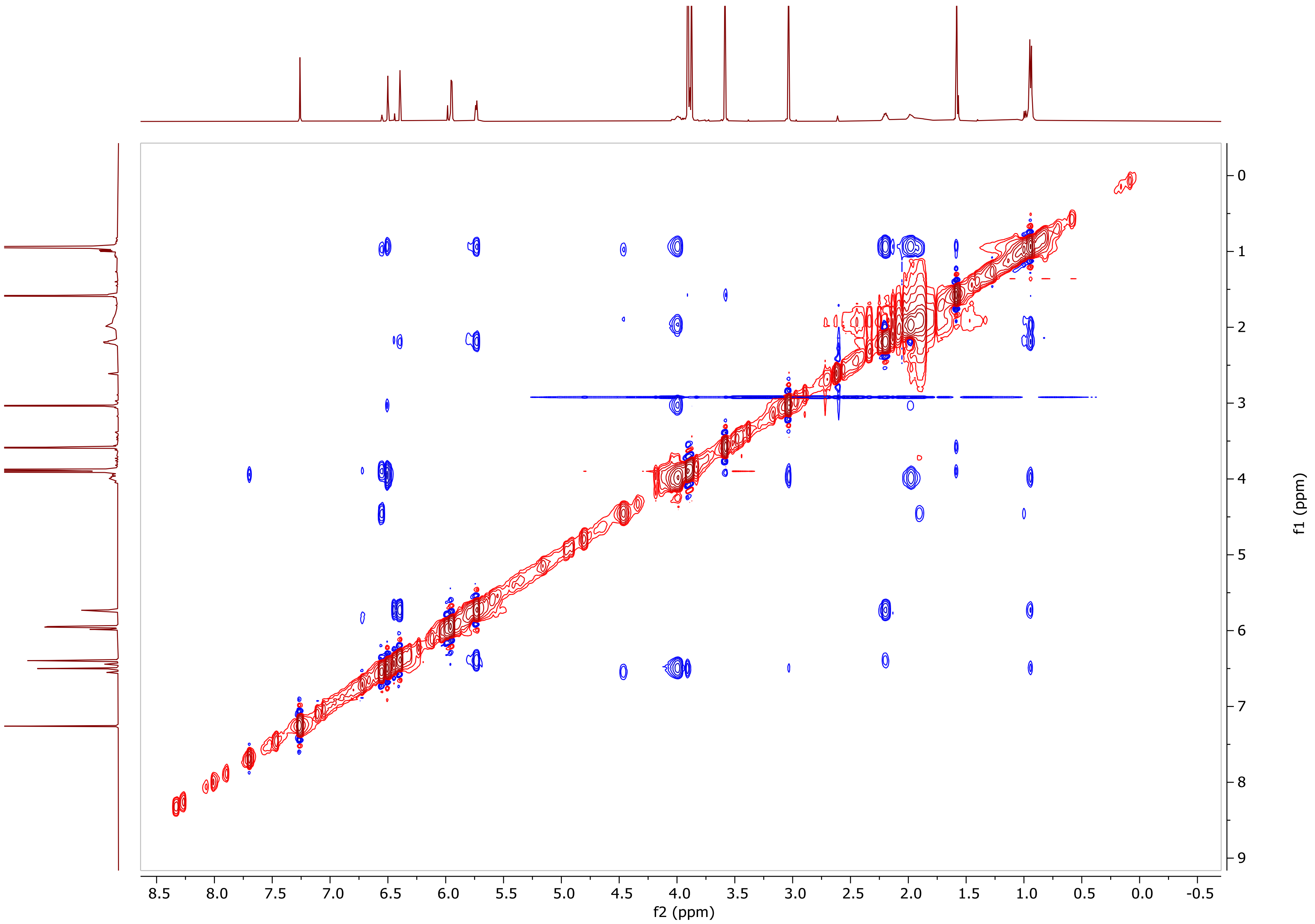

Figure S26. Zoomed NOESY NMR spectrum of **2** in chloroform-*d* at 323K (500 MHz)

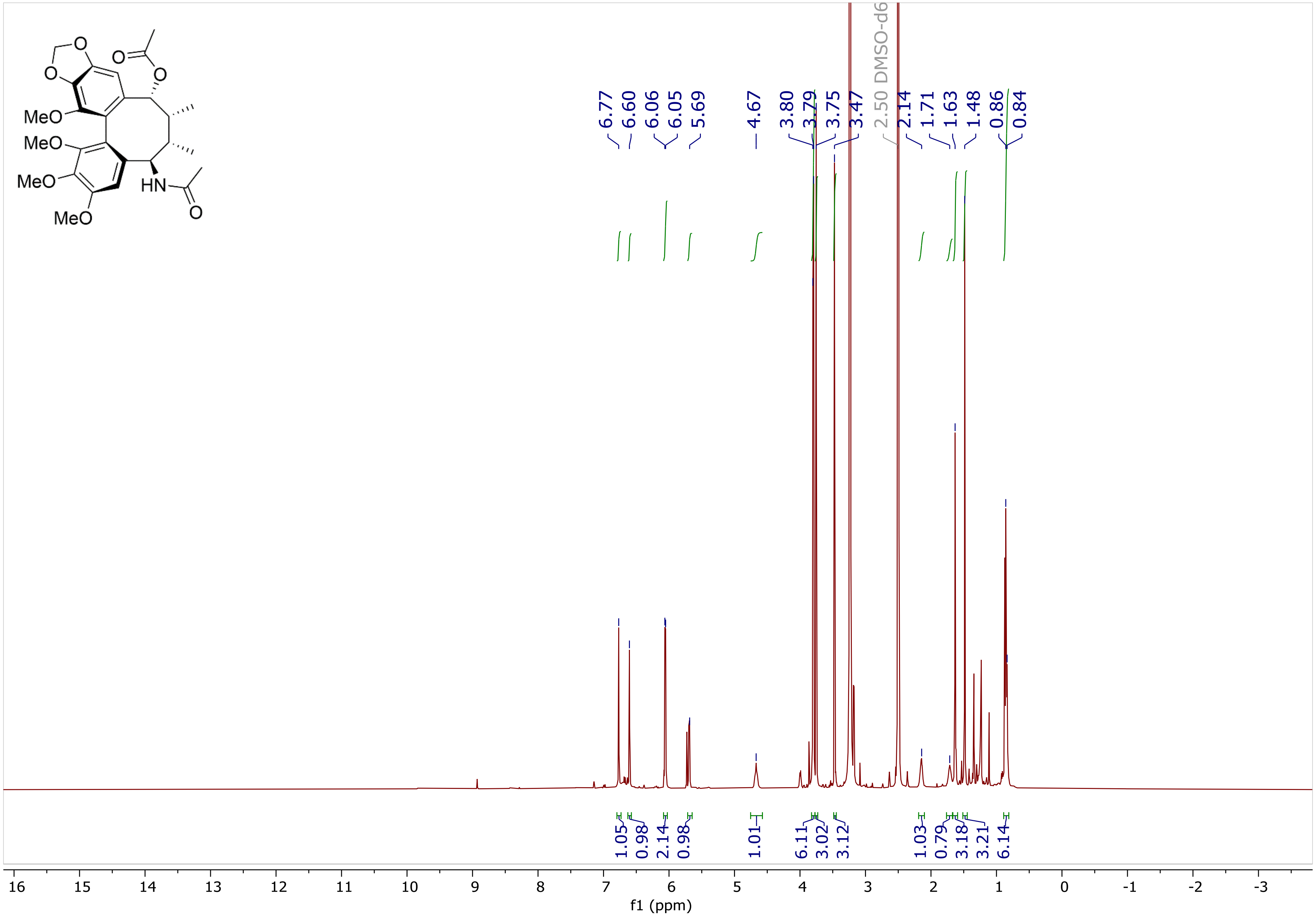

Figure S27. ^{1}H NMR spectrum of **3** in DMSO-d_6 at 323K (500 MHz)

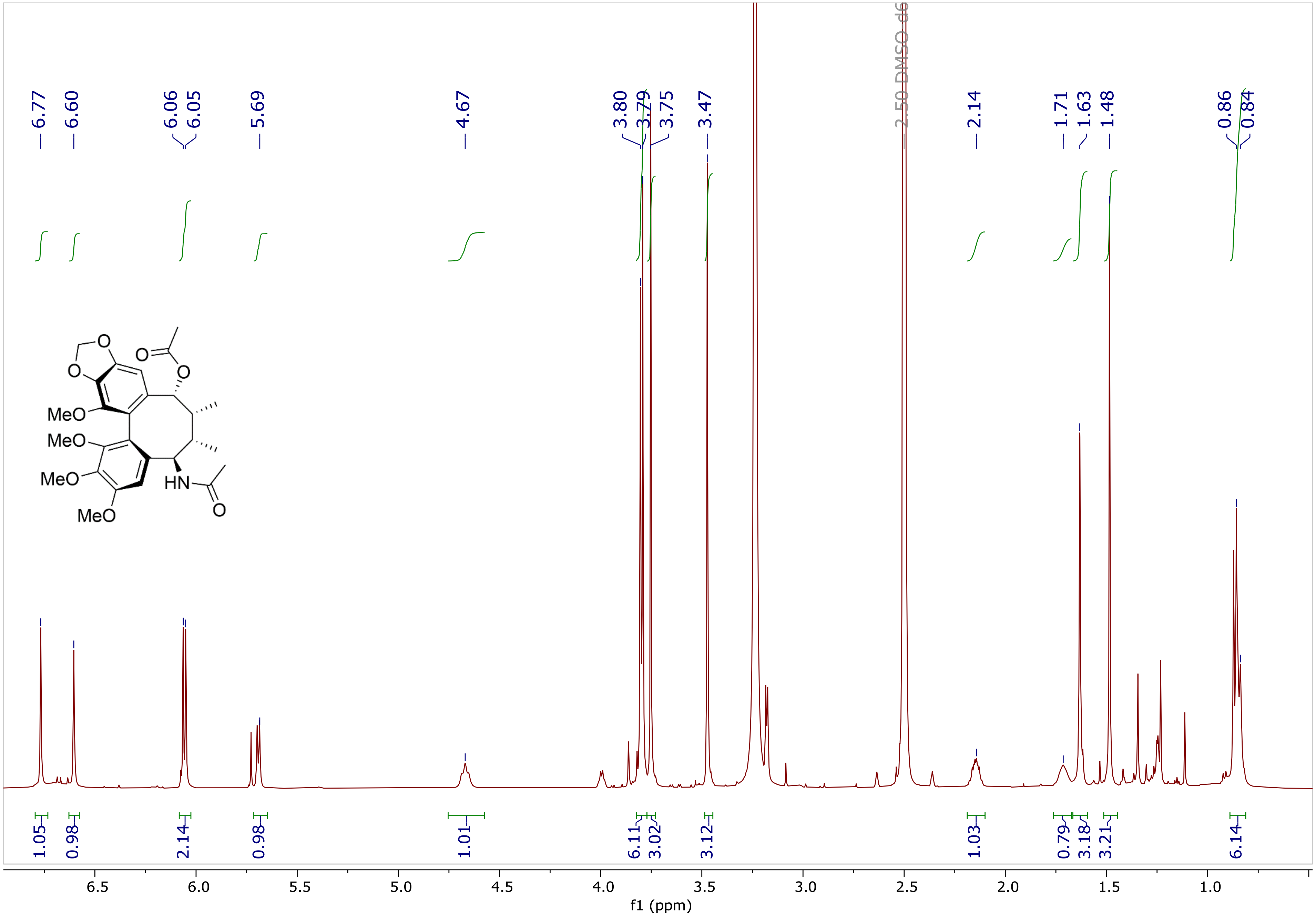

Figure S28. Zoomed ^{1}H NMR spectrum of **3** in DMSO-d_6 at 323K (500 MHz)

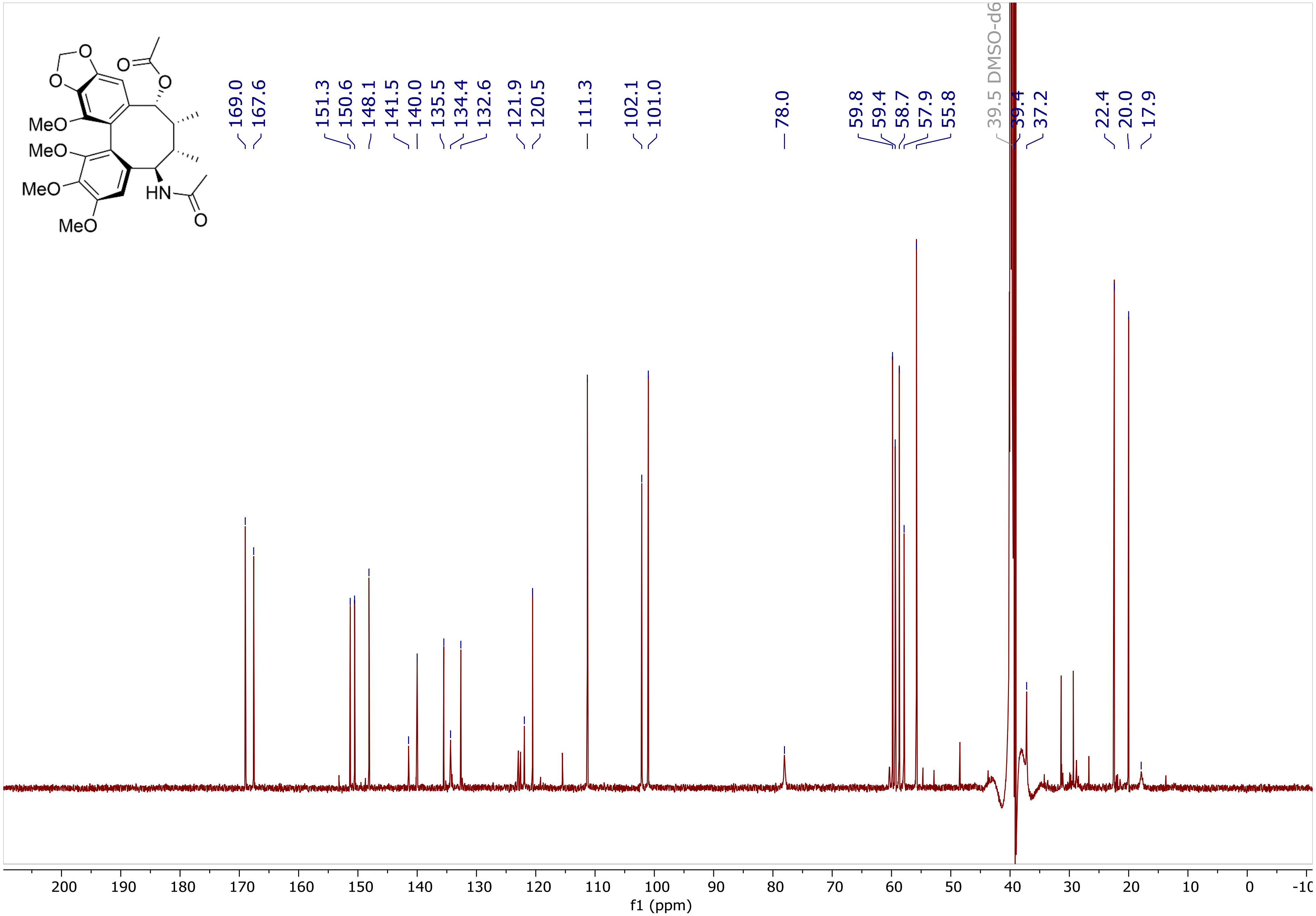

Figure S29. ${}^{13}C$ NMR spectrum of **3** in DMSO-d_6 at 323K (125 MHz)

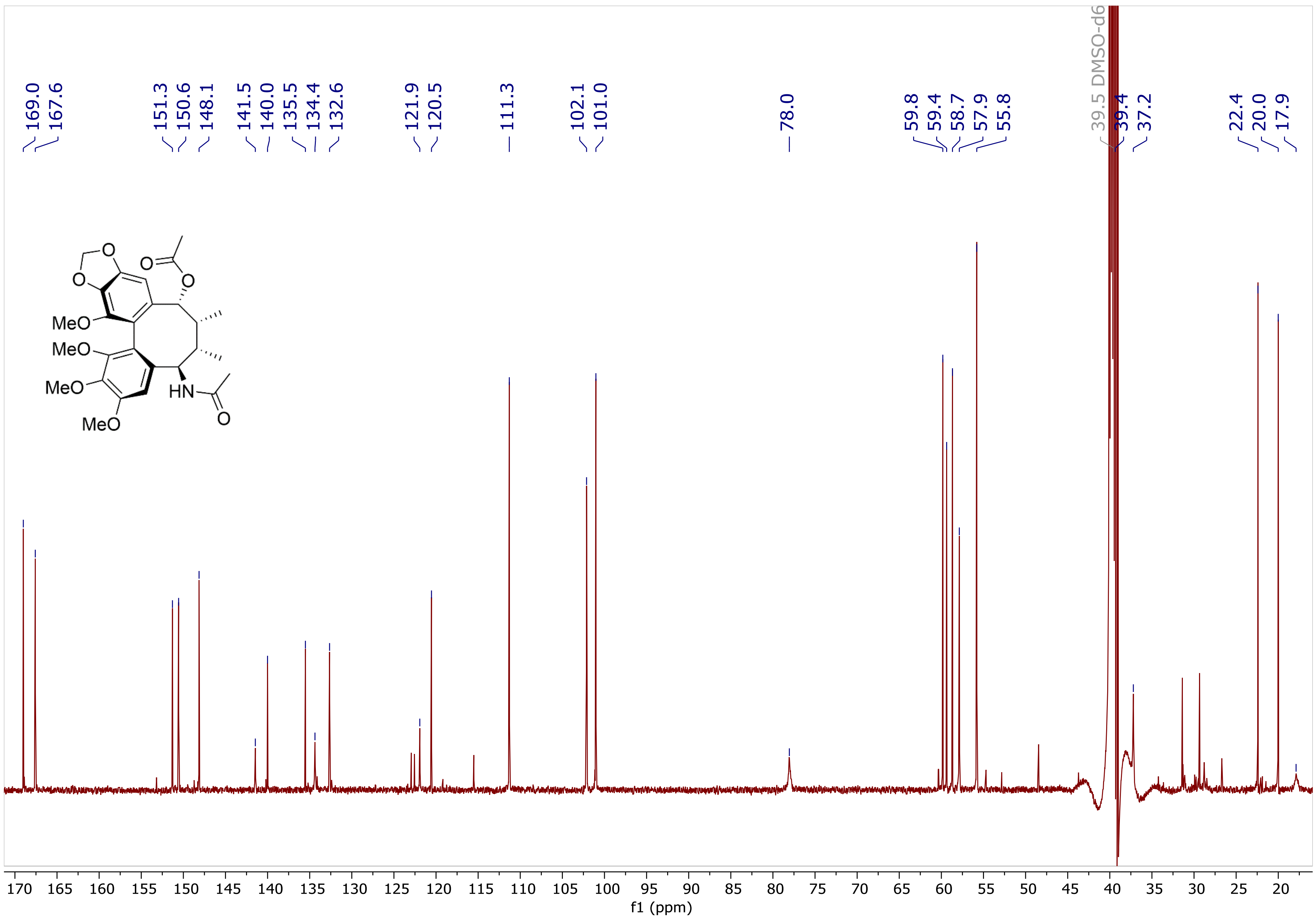

Figure S30. Zoomed ^{13}C NMR spectrum of **3** in DMSO-d_6 at 323K (125 MHz)

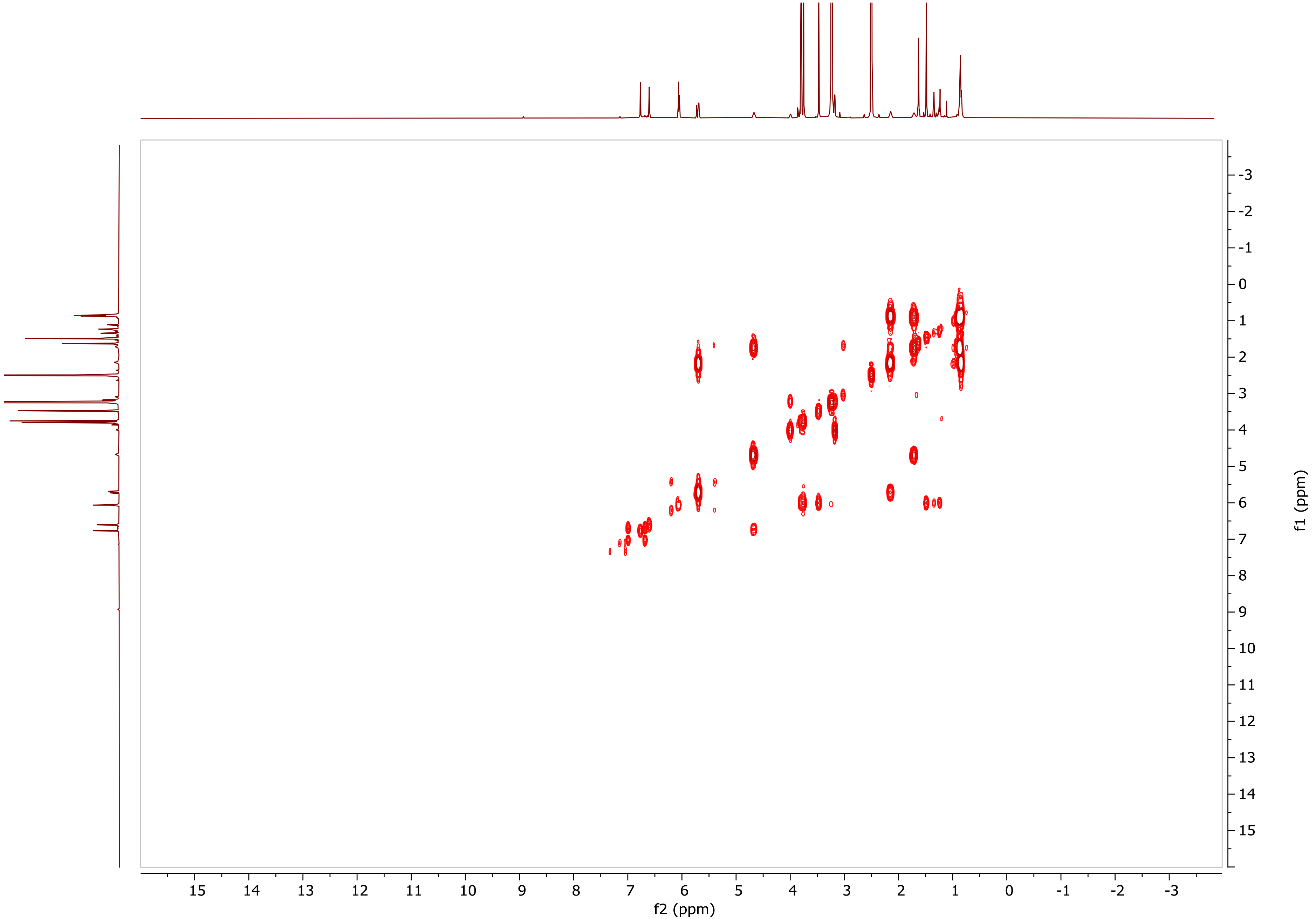

Figure S31. COSY NMR spectrum of **3** in DMSO-d_6 at 323K (500 MHz)

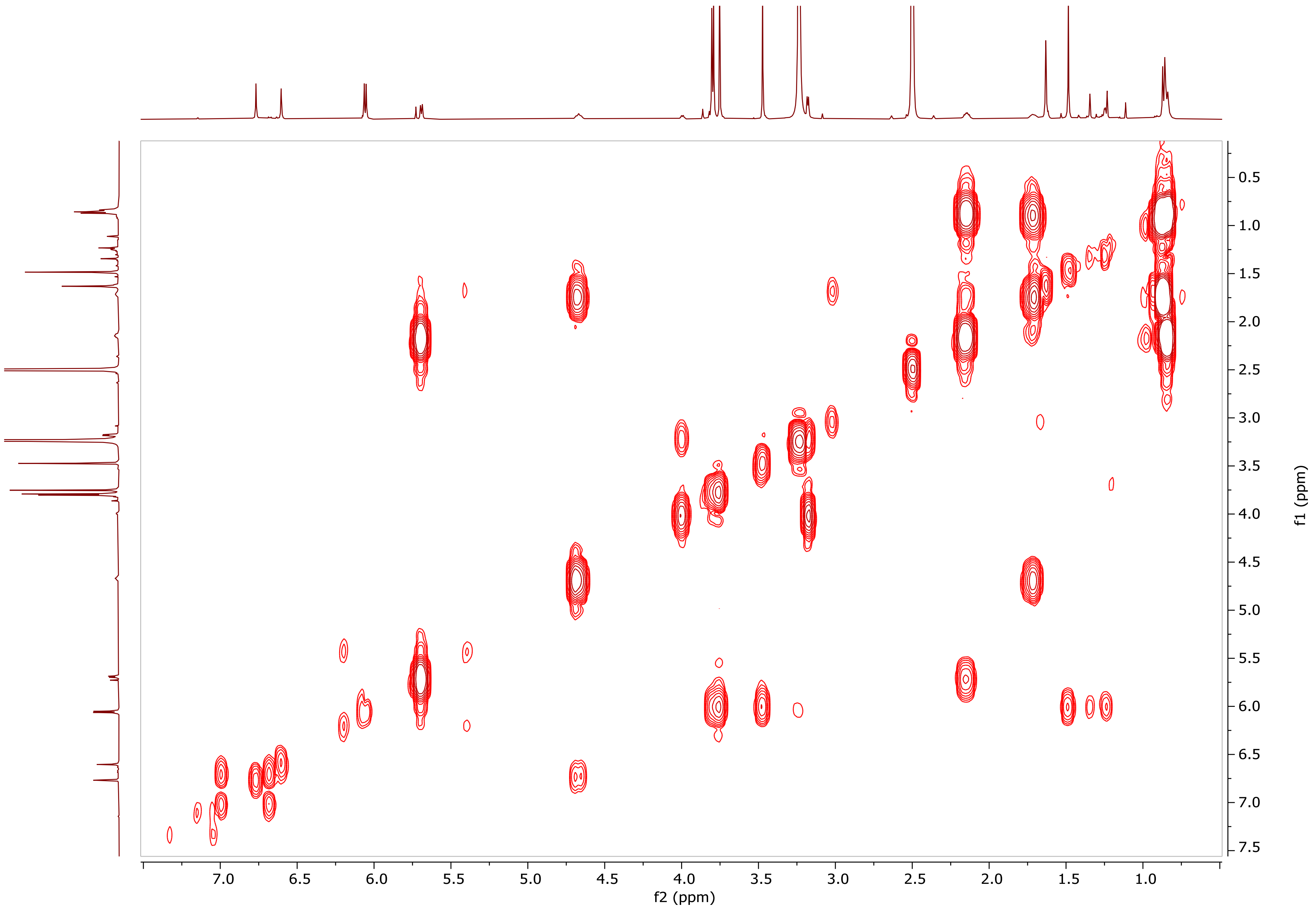

Figure S32. Zoomed COSY NMR spectrum of **3** in DMSO-d_6 at 323K (500 MHz)

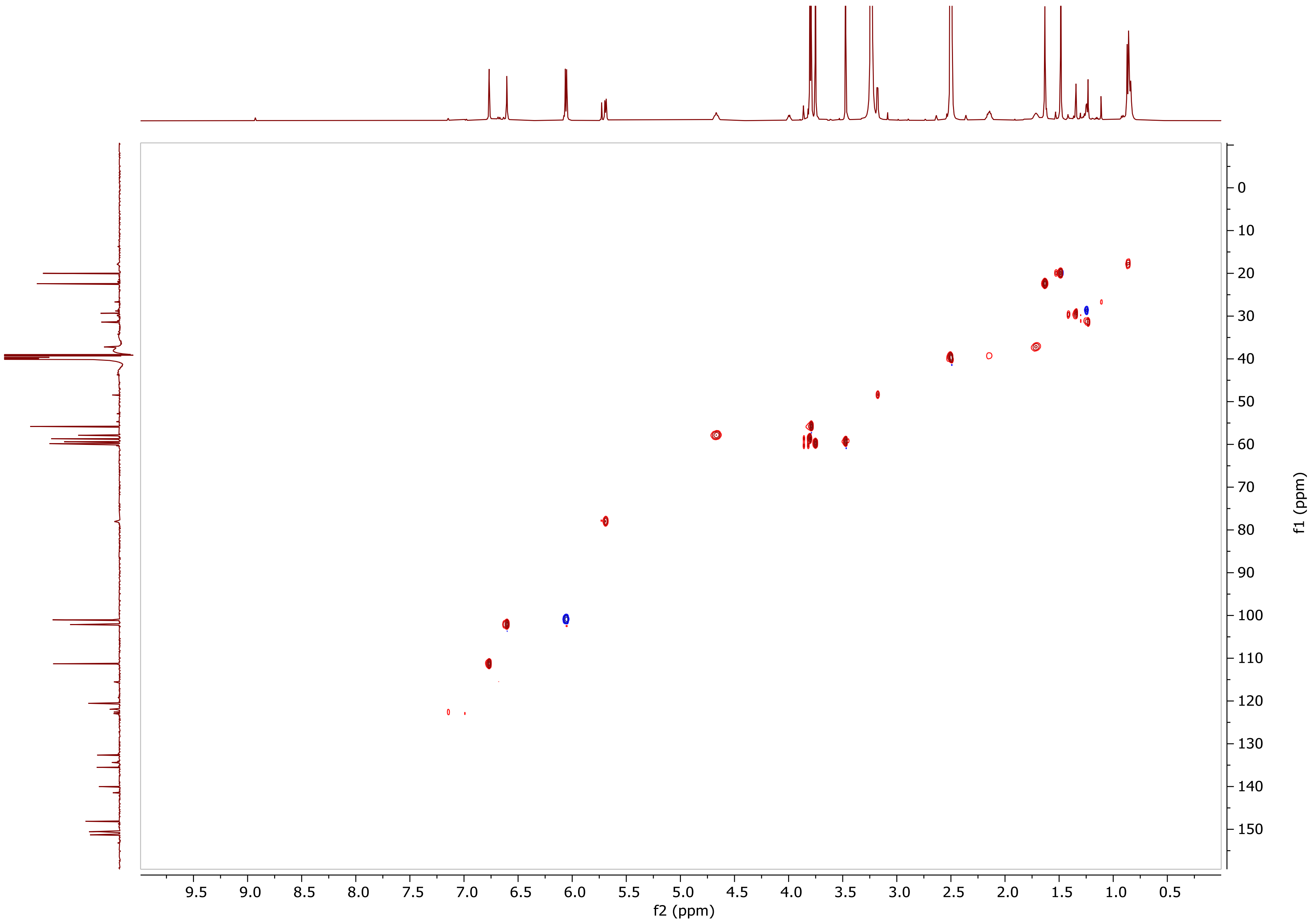

Figure S33. HSQC NMR spectrum of **3** in DMSO-d_6 at 323K (500 MHz)

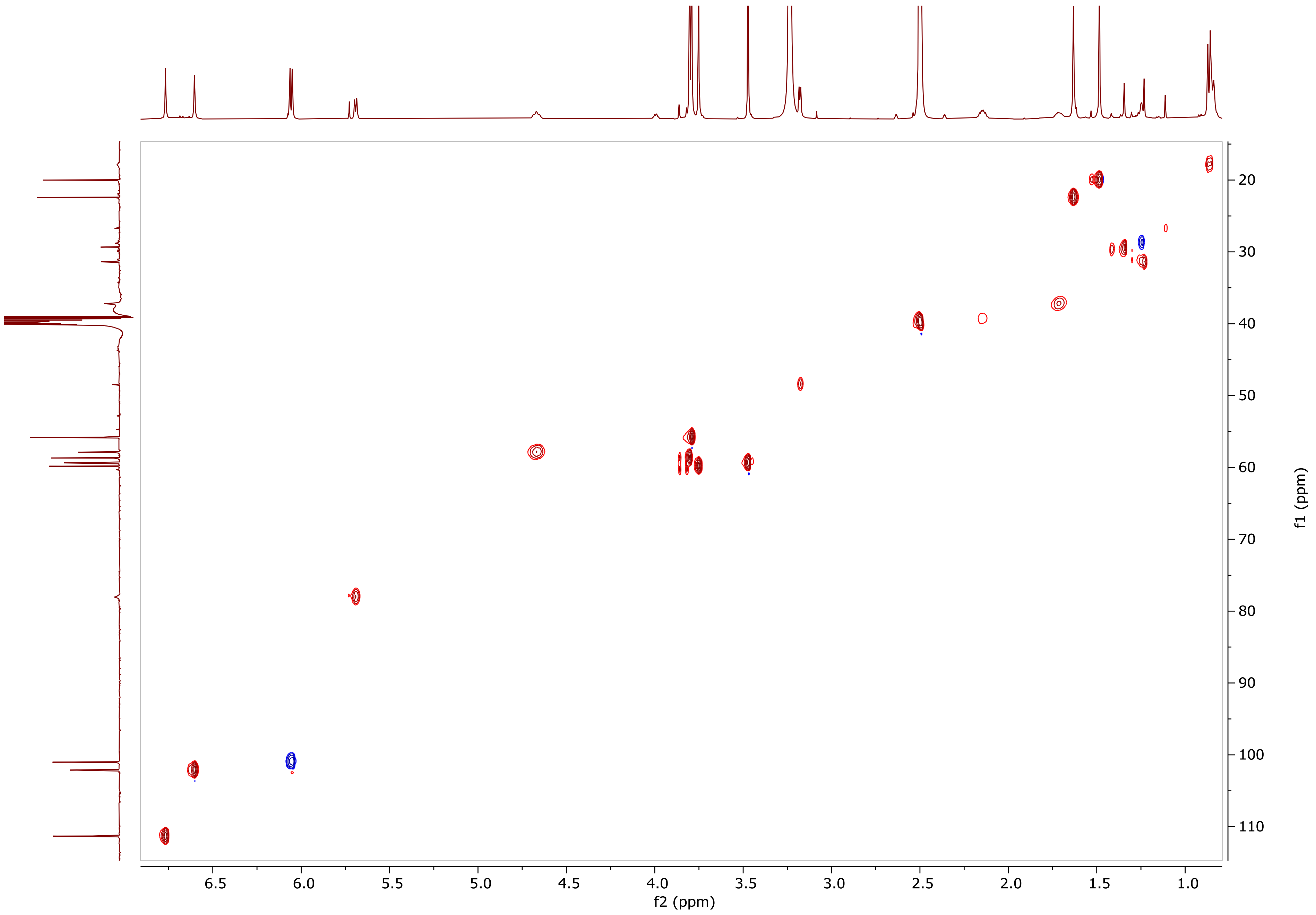

Figure S34. Zoomed HSQC NMR spectrum of **3** in DMSO-d_6 at 323K (500 MHz)

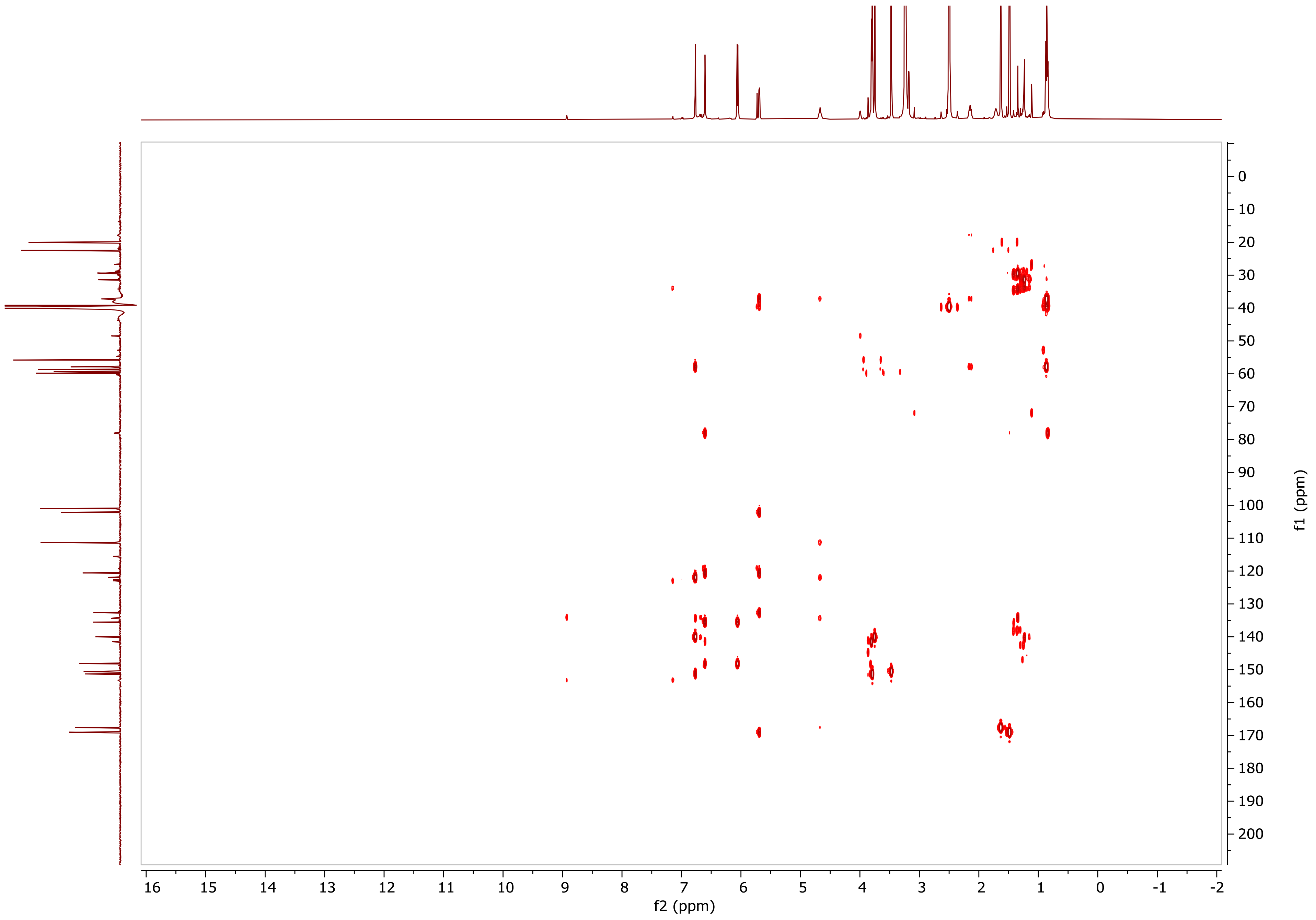

Figure S35. HMBC NMR spectrum of **3** in DMSO-d_6 at 323K (500 MHz)

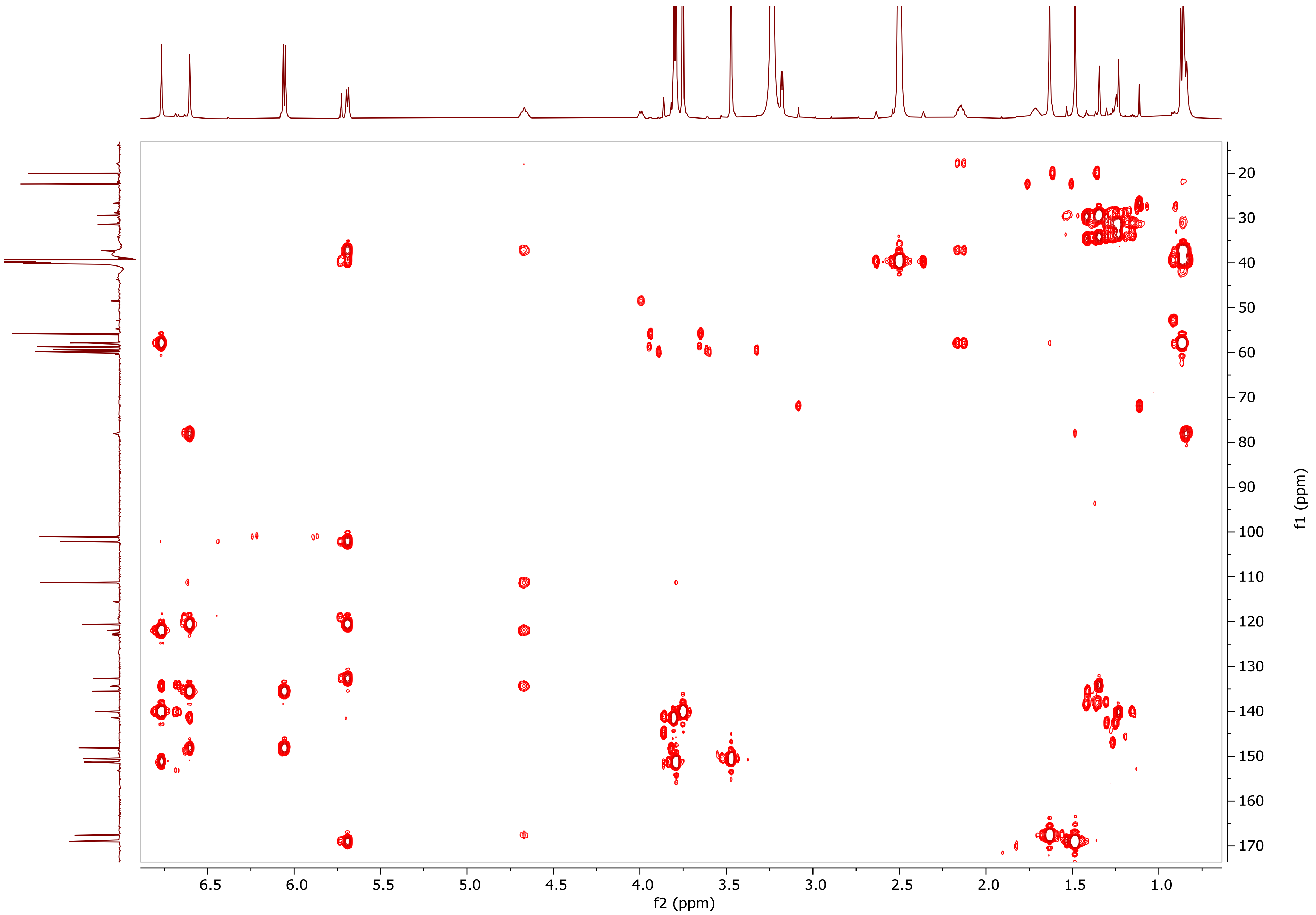

Figure S36. Zoomed HMBC NMR spectrum of **3** in DMSO-d_6 at 323K (500 MHz)

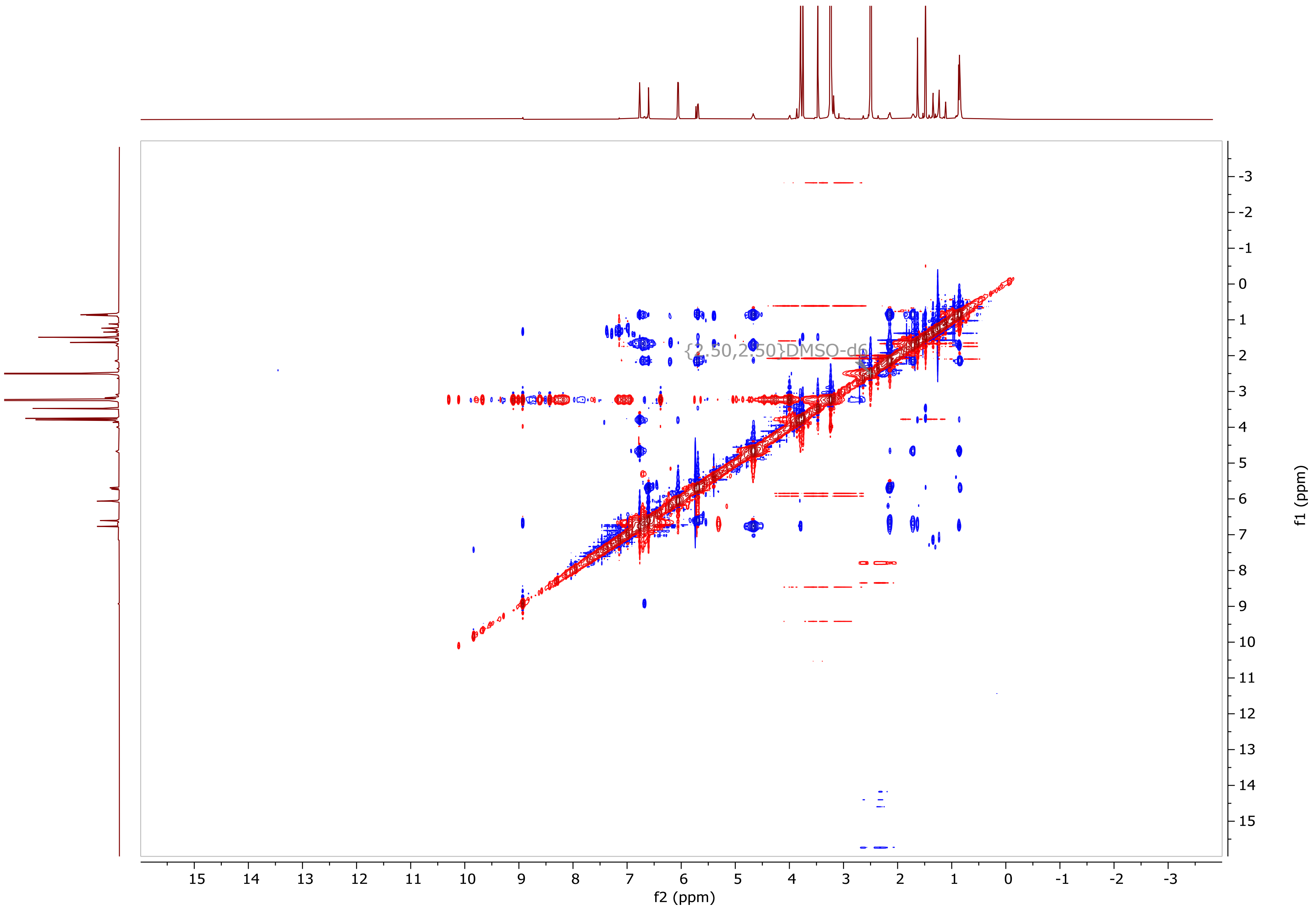

Figure S37. NOESY NMR spectrum of **3** in DMSO-d_6 at 323K (500 MHz)

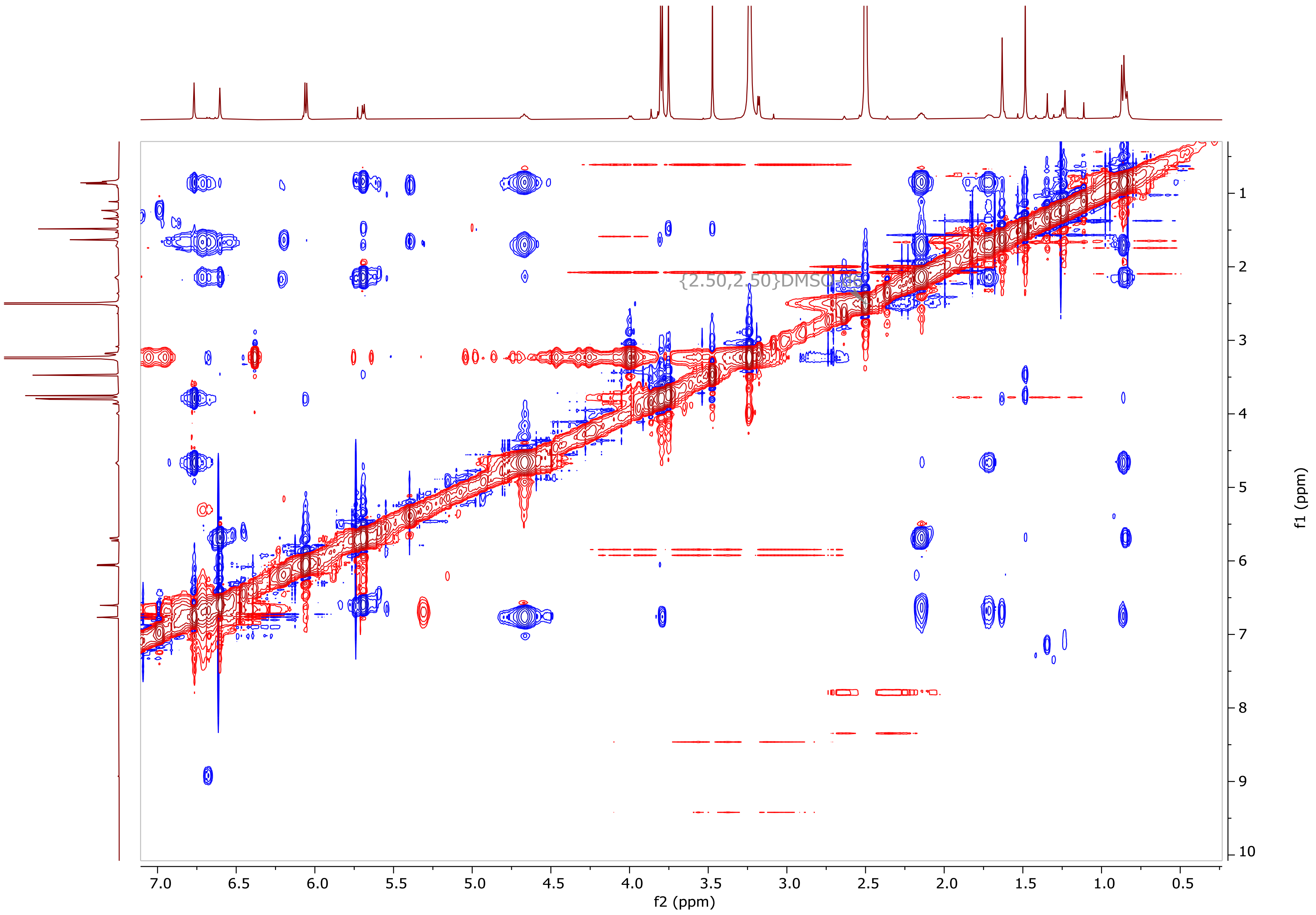

Figure S38. Zoomed NOESY NMR spectrum of **3** in DMSO-d_6 at 323K (500 MHz)

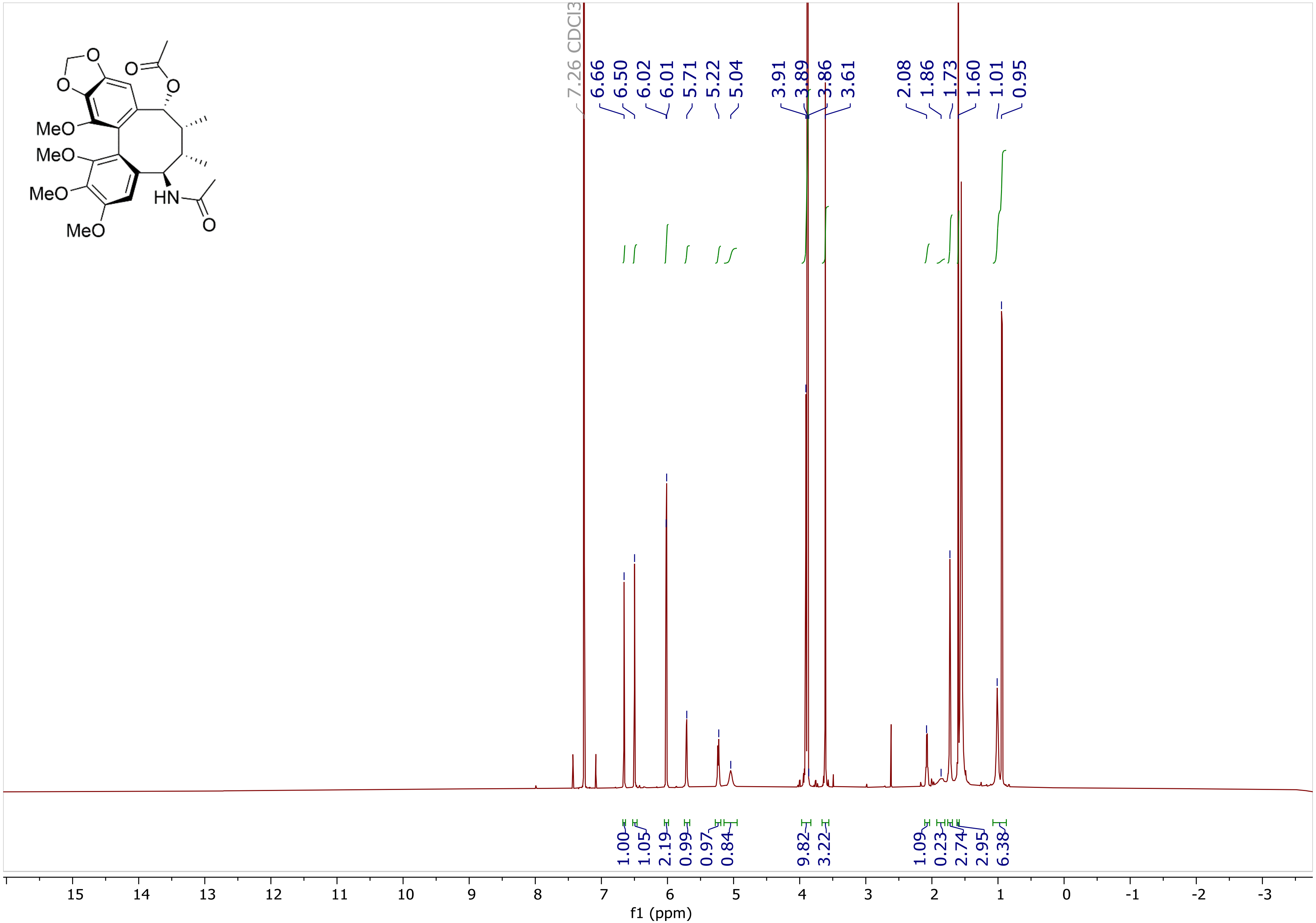

Figure S39. ^{1}H NMR spectrum of **3** in $CDCl_3$ at 298K (600 MHz)

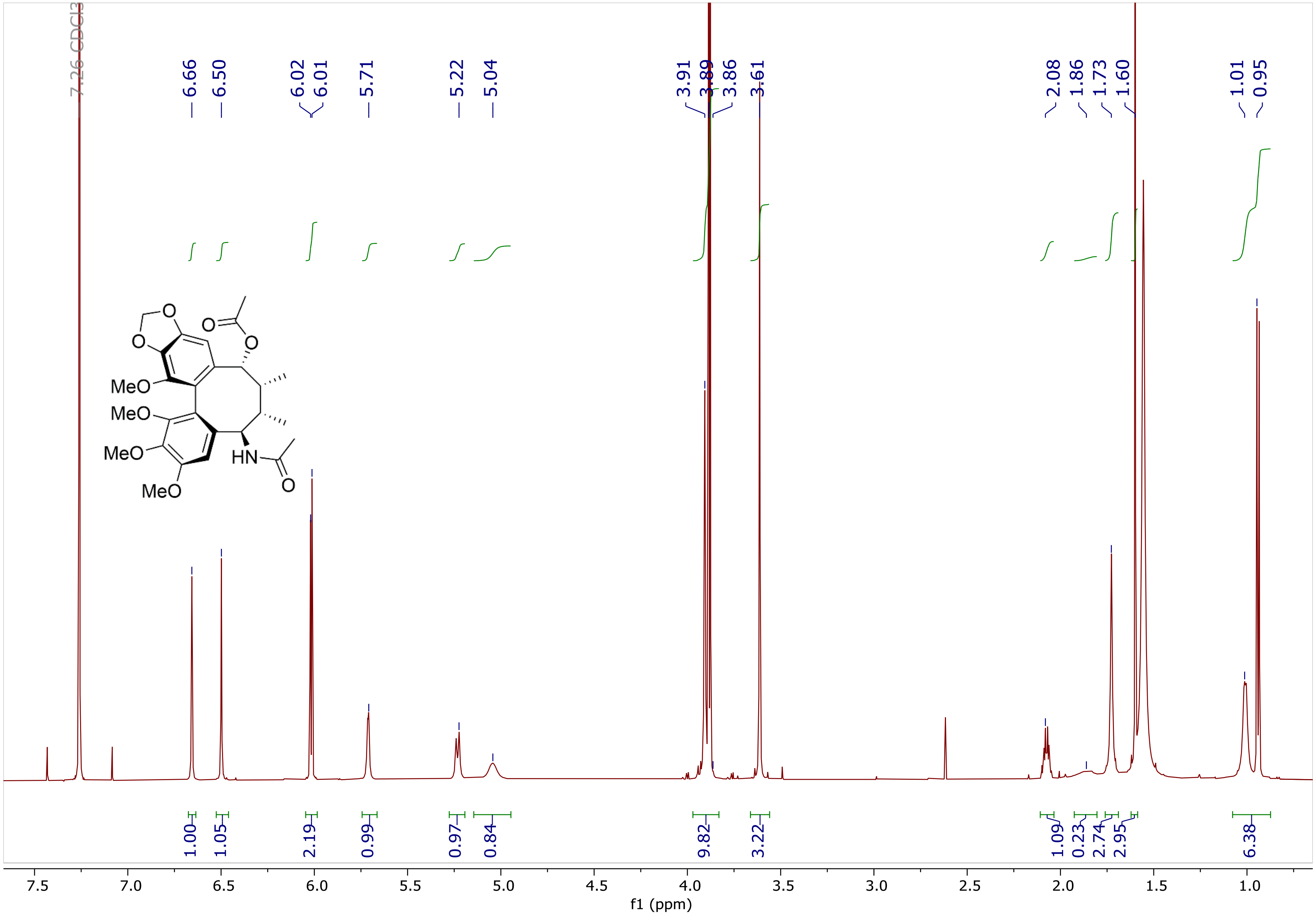

Figure S40. Zoomed ^{1}H NMR spectrum of **3** in $CDCl_3$ at 298K (600 MHz)

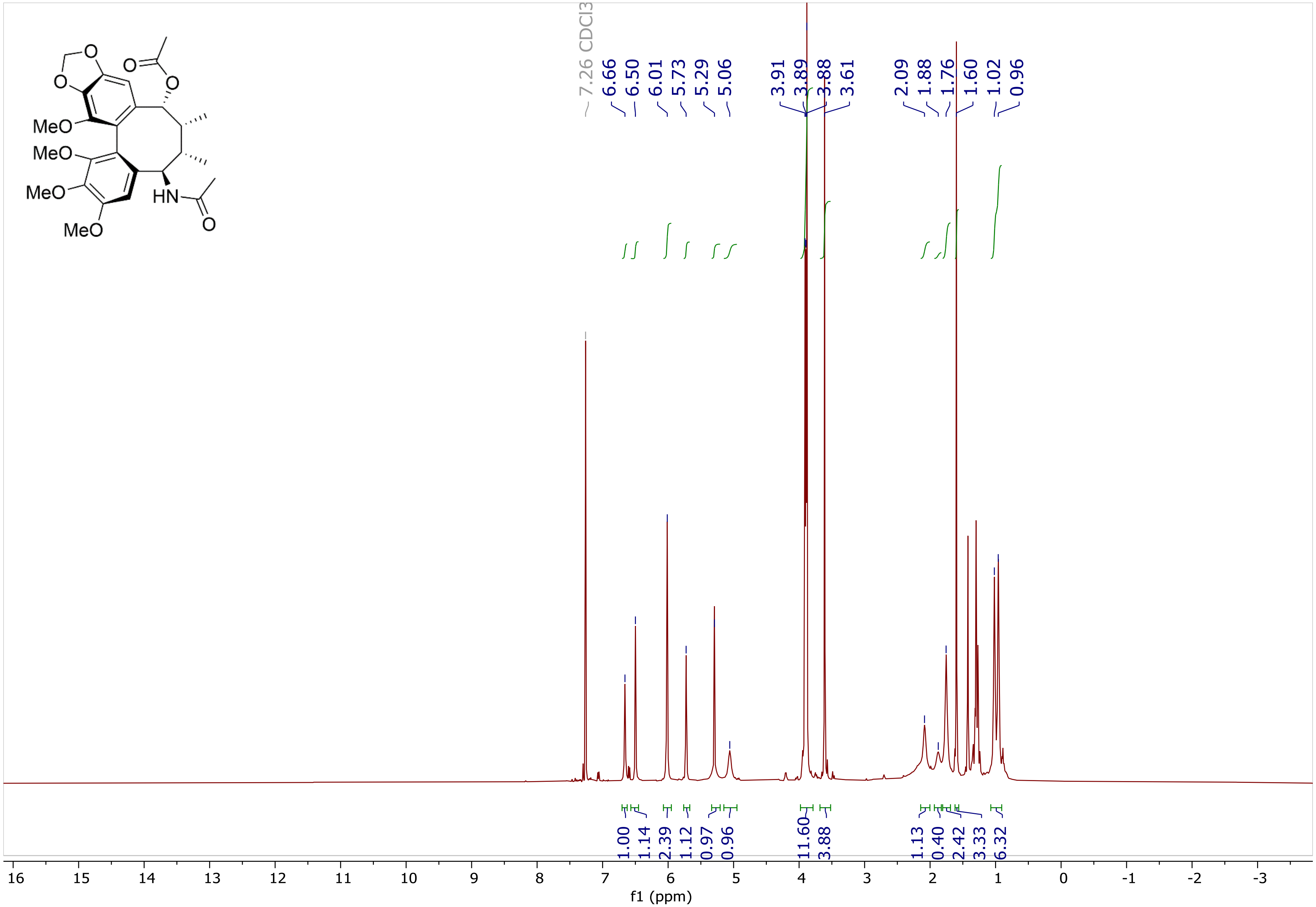

Figure S41. ^{1}H NMR spectrum of **3** in $CDCl_3$ at 323K (500 MHz).

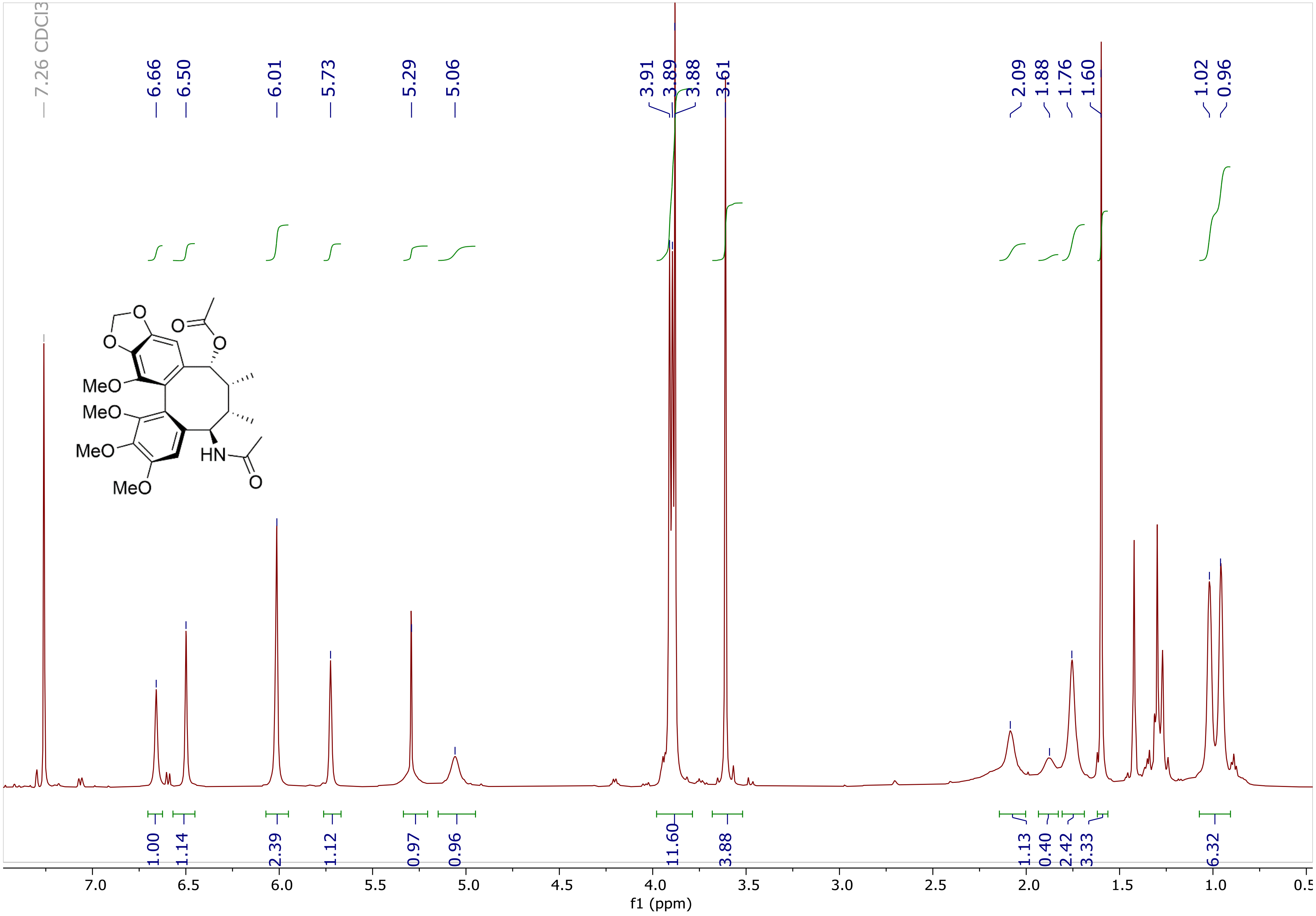

Figure S42. Zoomed ^{1}H NMR spectrum of **3** in $CDCl_3$ at 323K (500 MHz)

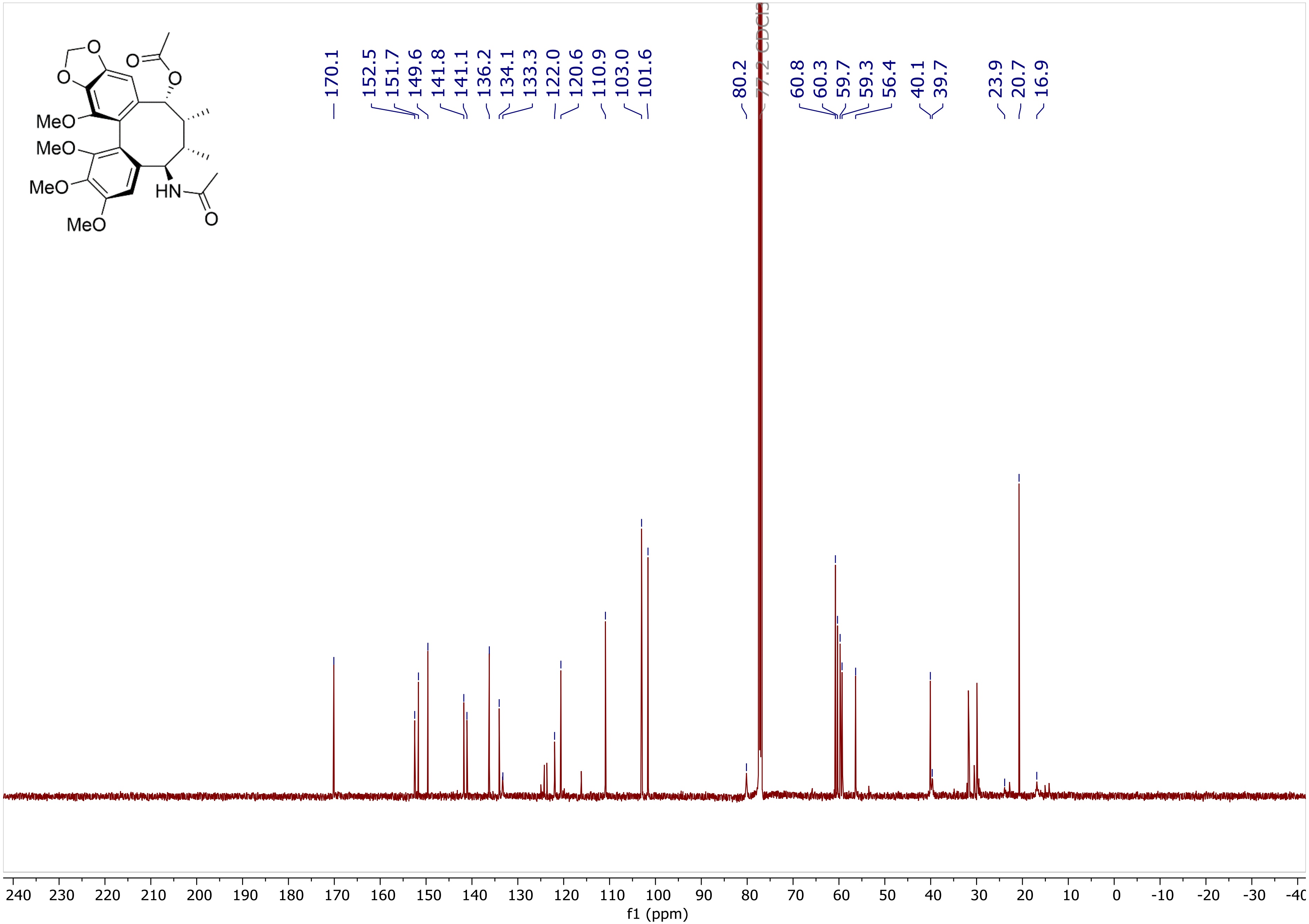

Figure S43. ^{13}C NMR spectrum of **3** in $CDCl_3$ at 323K (125 MHz)

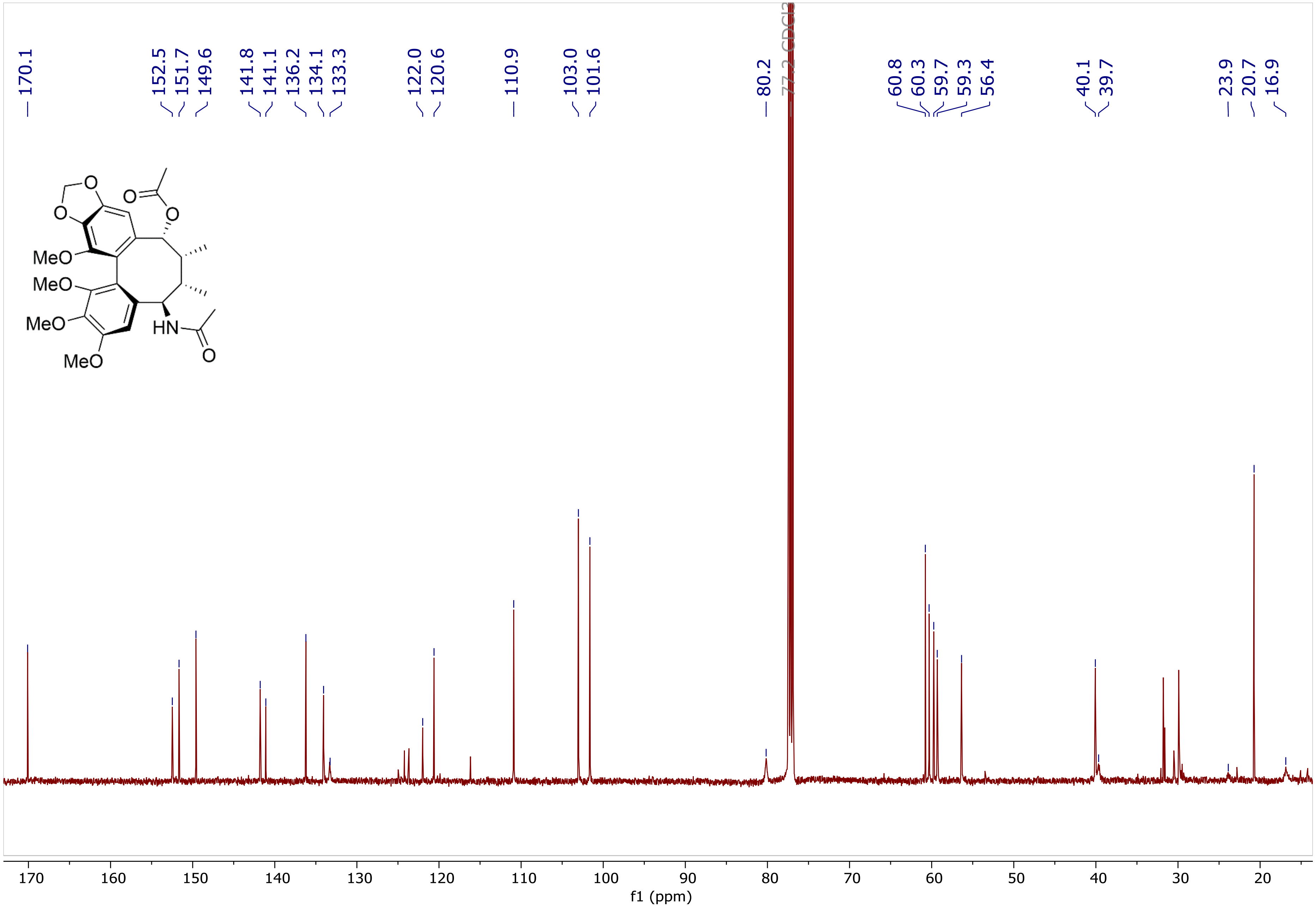

Figure S44. Zoomed ^{13}C NMR spectrum of **3** in $CDCl_3$ at 323K (125 MHz)

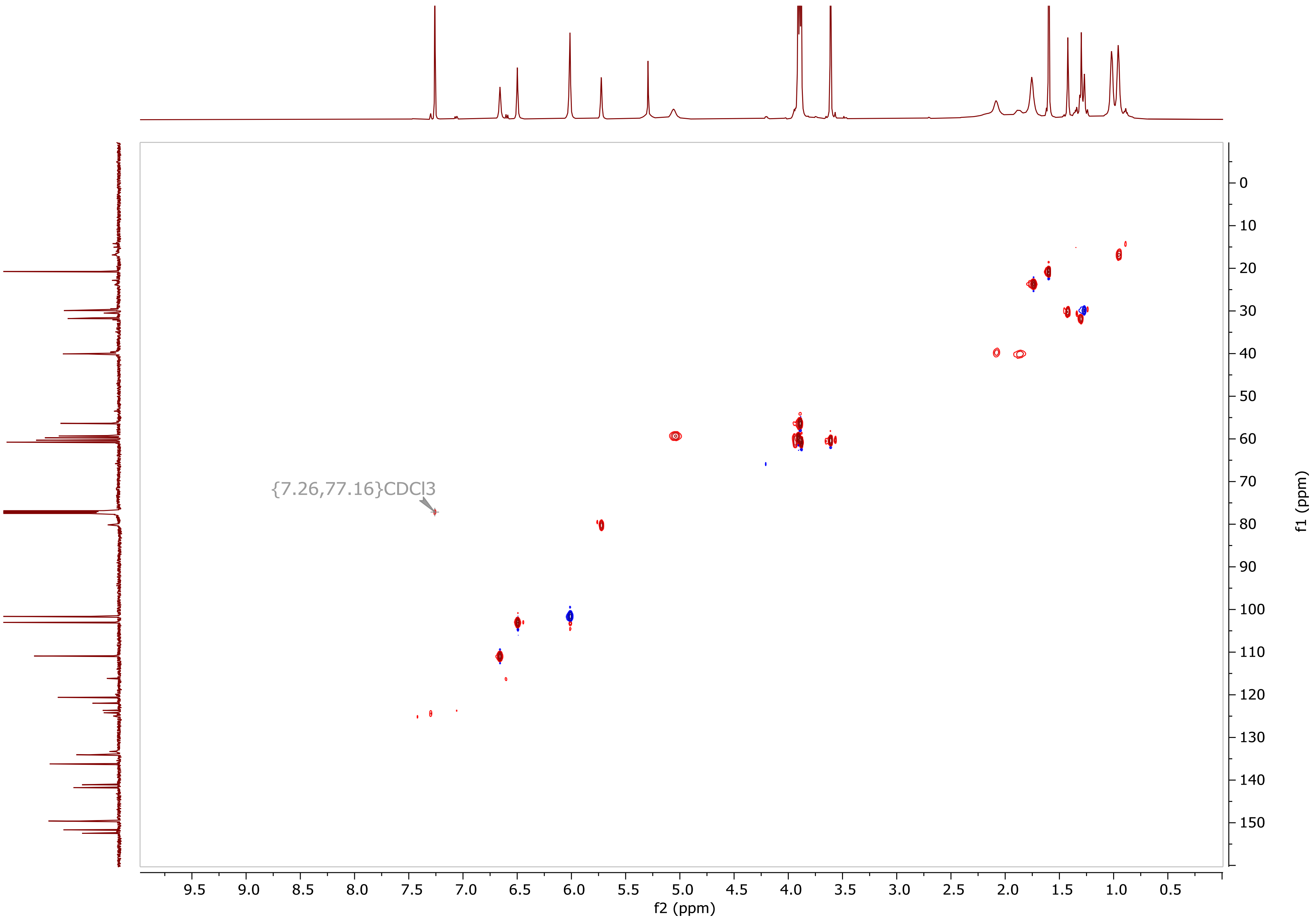

Figure S45. HSQC NMR spectrum of **3** in $CDCl_3$ at 323K (500 MHz).

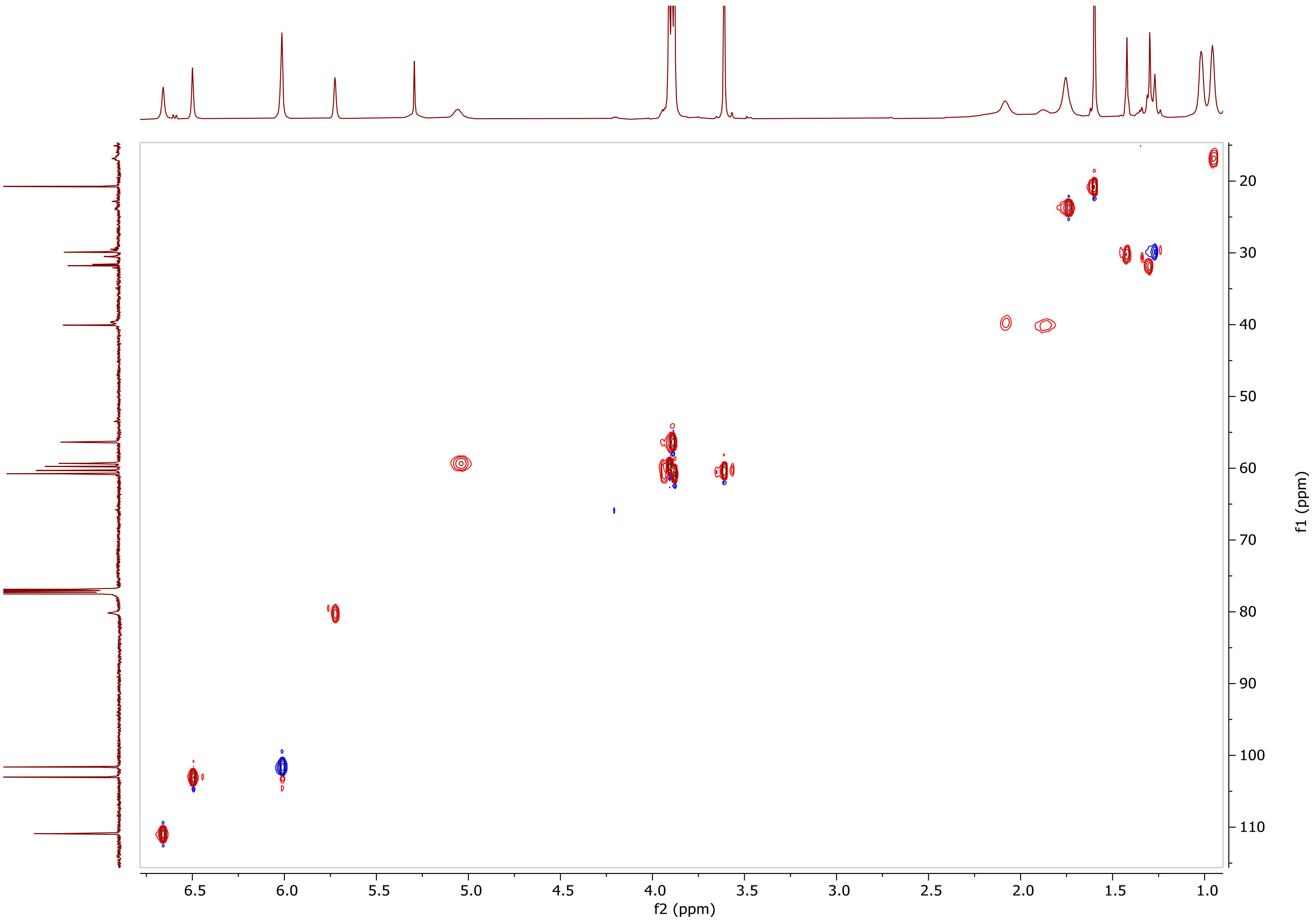

Figure S46. Zoomed HSQC NMR spectrum of **3** in $CDCl_3$ at 323K (500 MHz).

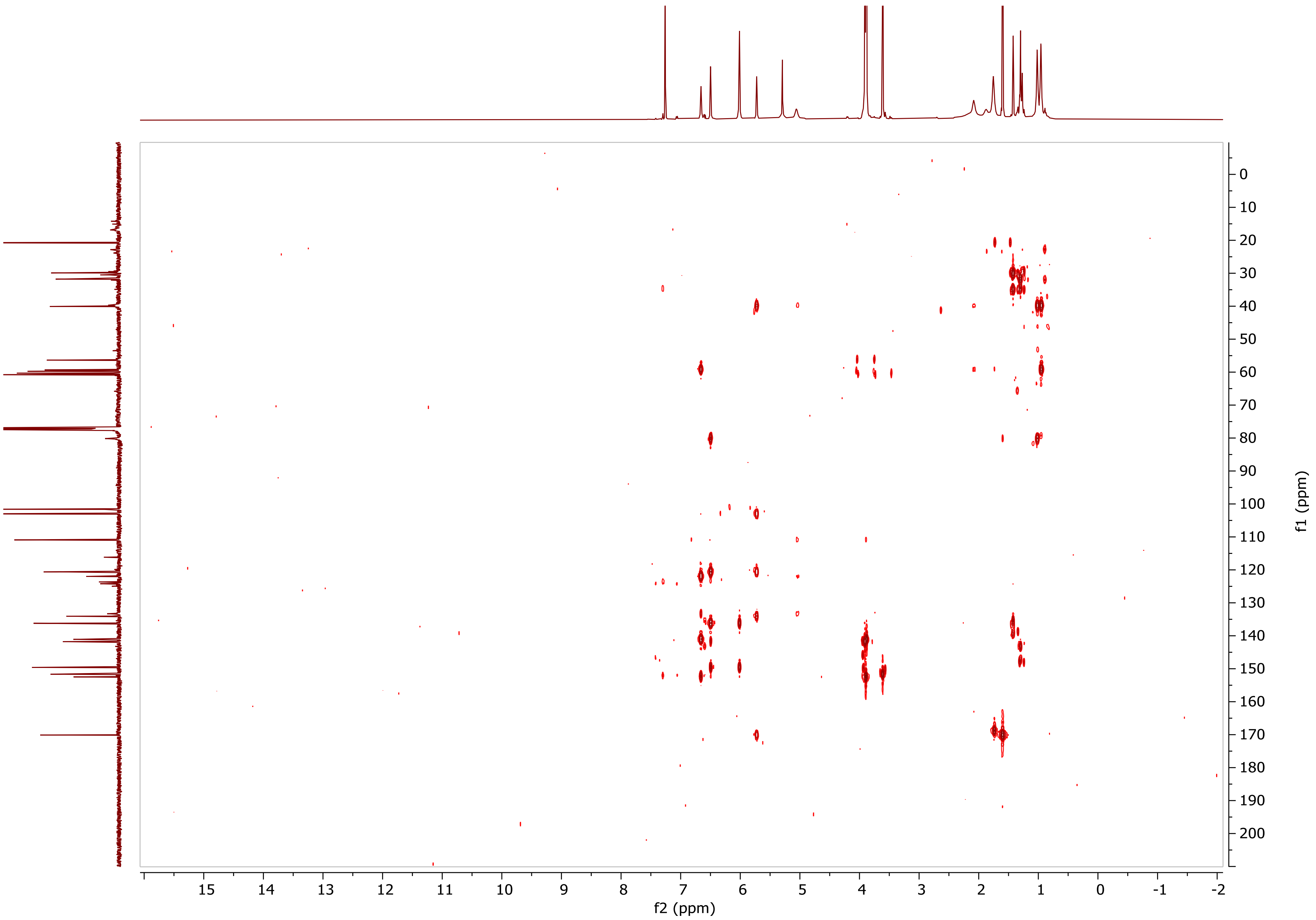

Figure S47. HMBC NMR spectrum of **3** in $CDCl_3$ at 323K (500 MHz).

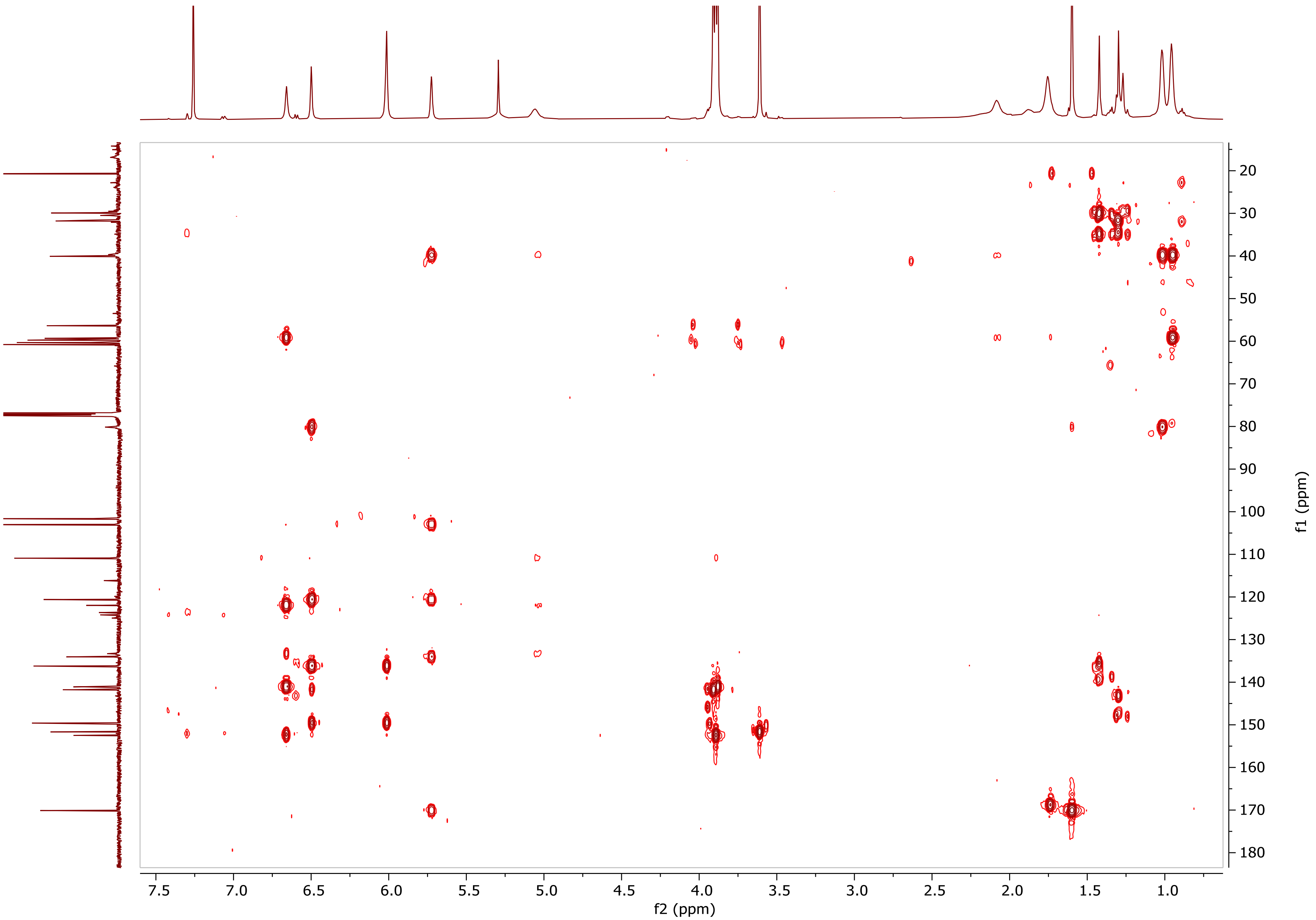

Figure S48. Zoomed HMBC NMR spectrum of **3** in $CDCl_3$ at 323K (500 MHz).

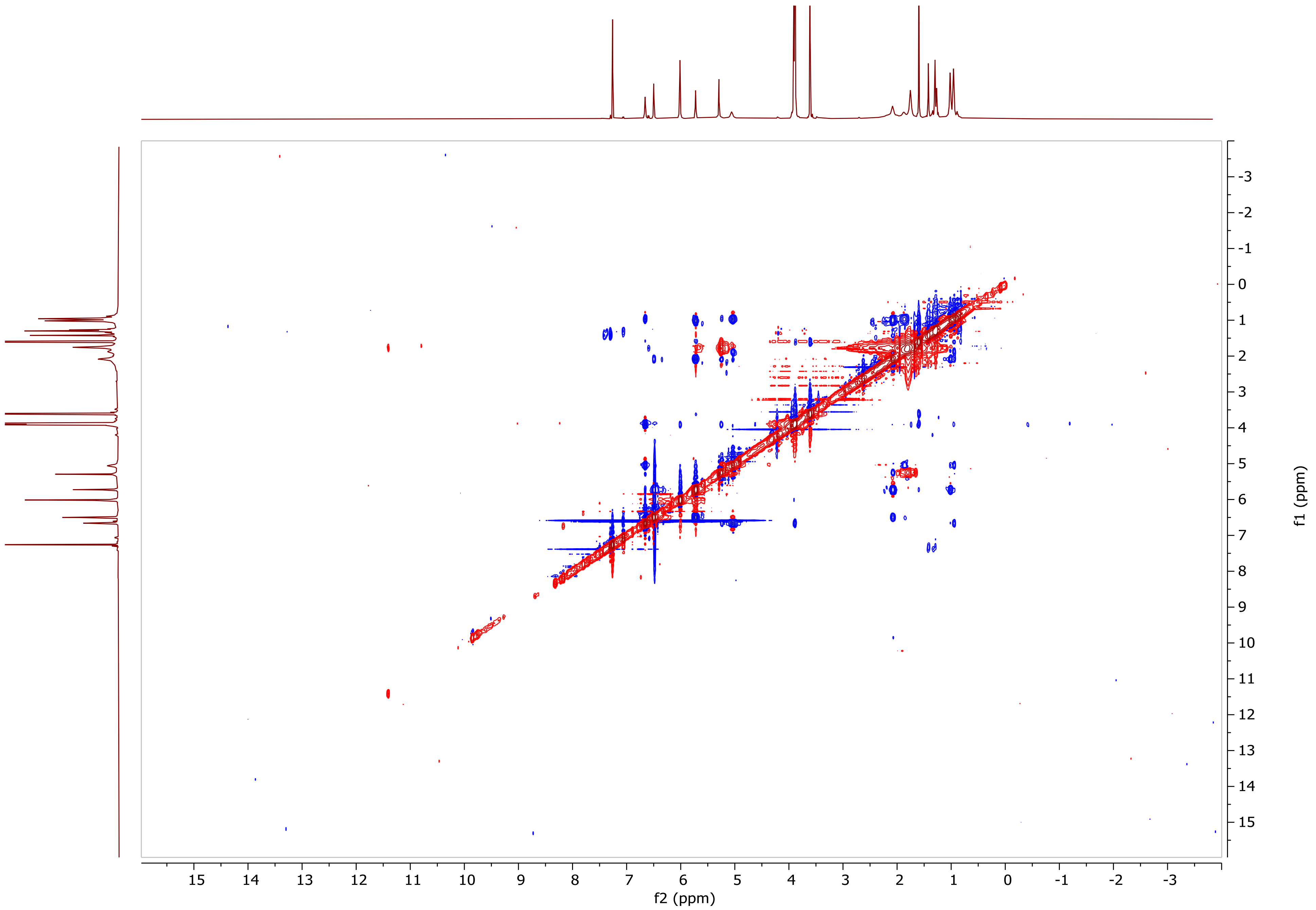

Figure S49. NOESY NMR spectrum of **3** in $CDCl_3$ at 323K (500 MHz).

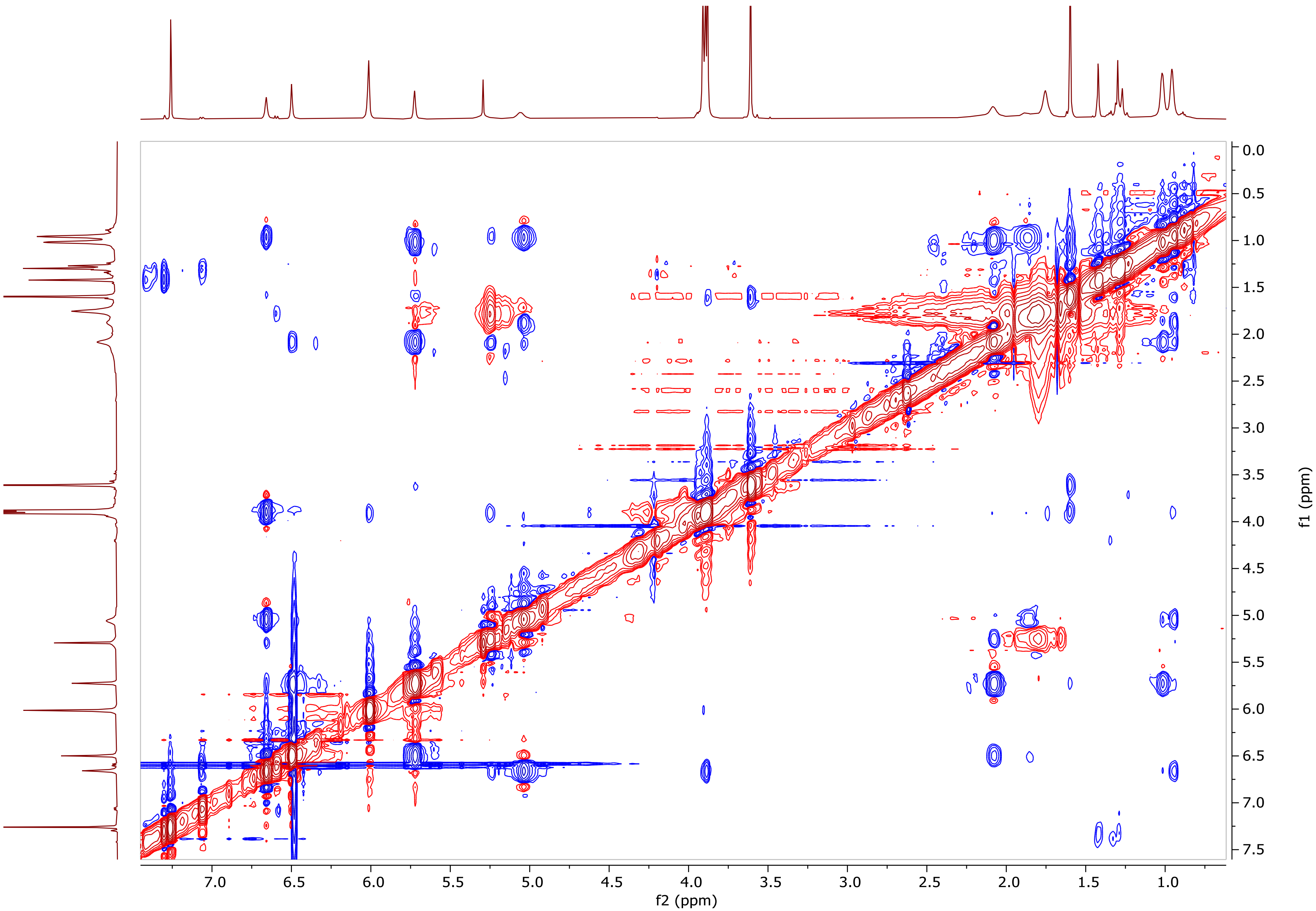

Figure S50. Zoomed NOESY NMR spectrum of **3** in $CDCl_3$ at 323K (500 MHz).

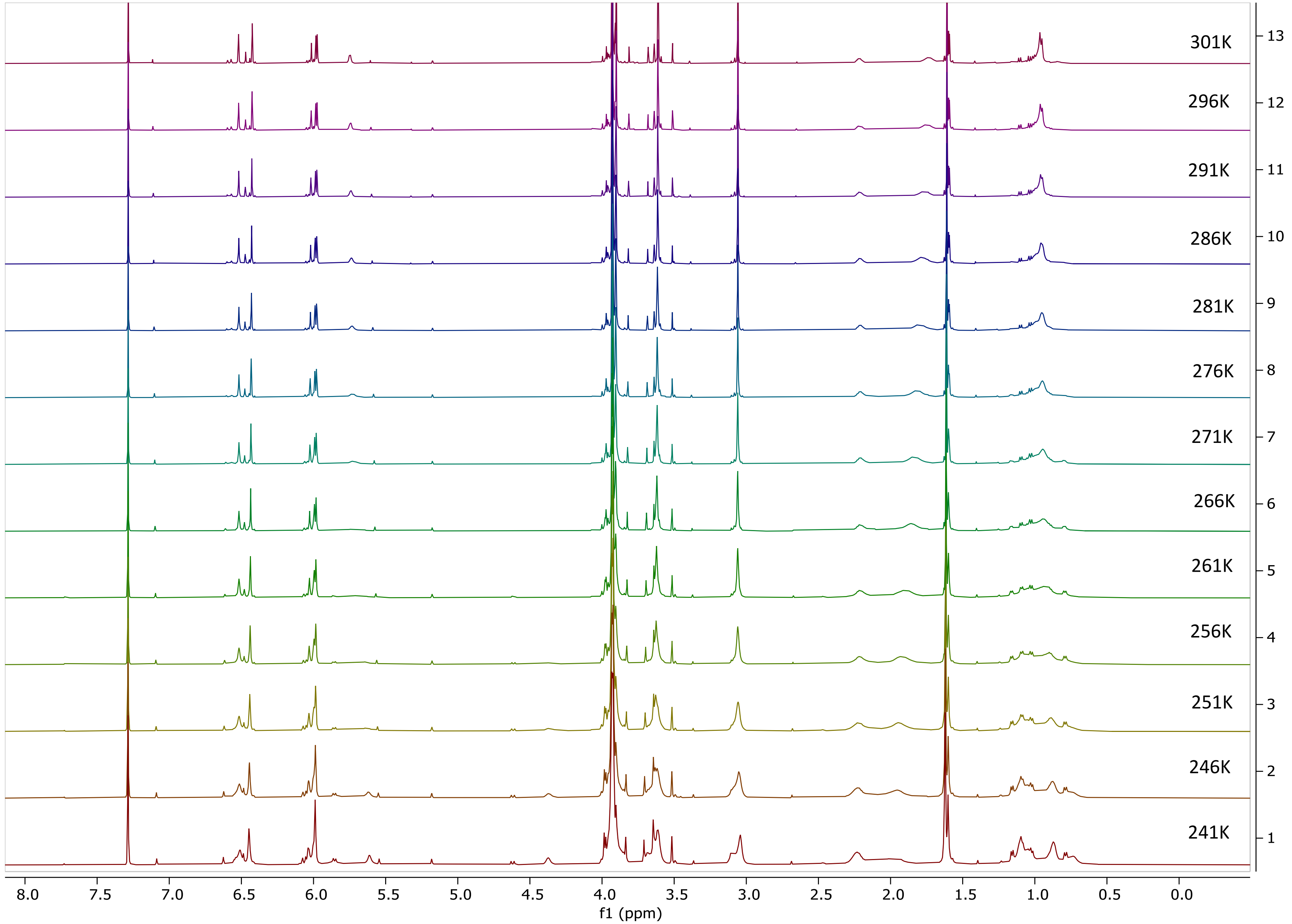

Figure S51. ^{1}H VT-NMR data for **2** in $CDCl_3$ (241-301K) (500 MHz).

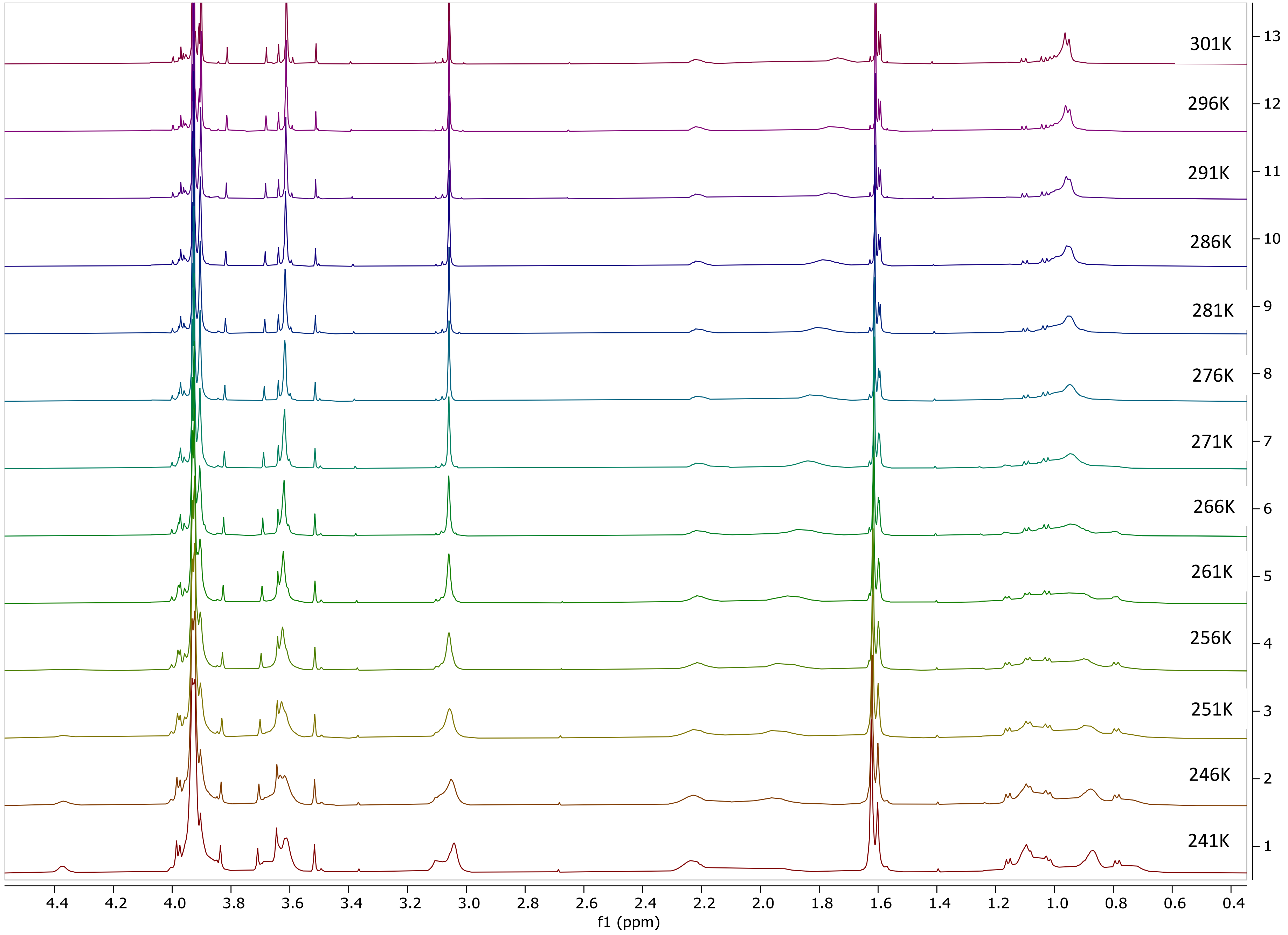

Figure S52. Upfield zoomed ^{1}H VT-NMR data for **2** in $CDCl_3$ (241-301K) (500 MHz).

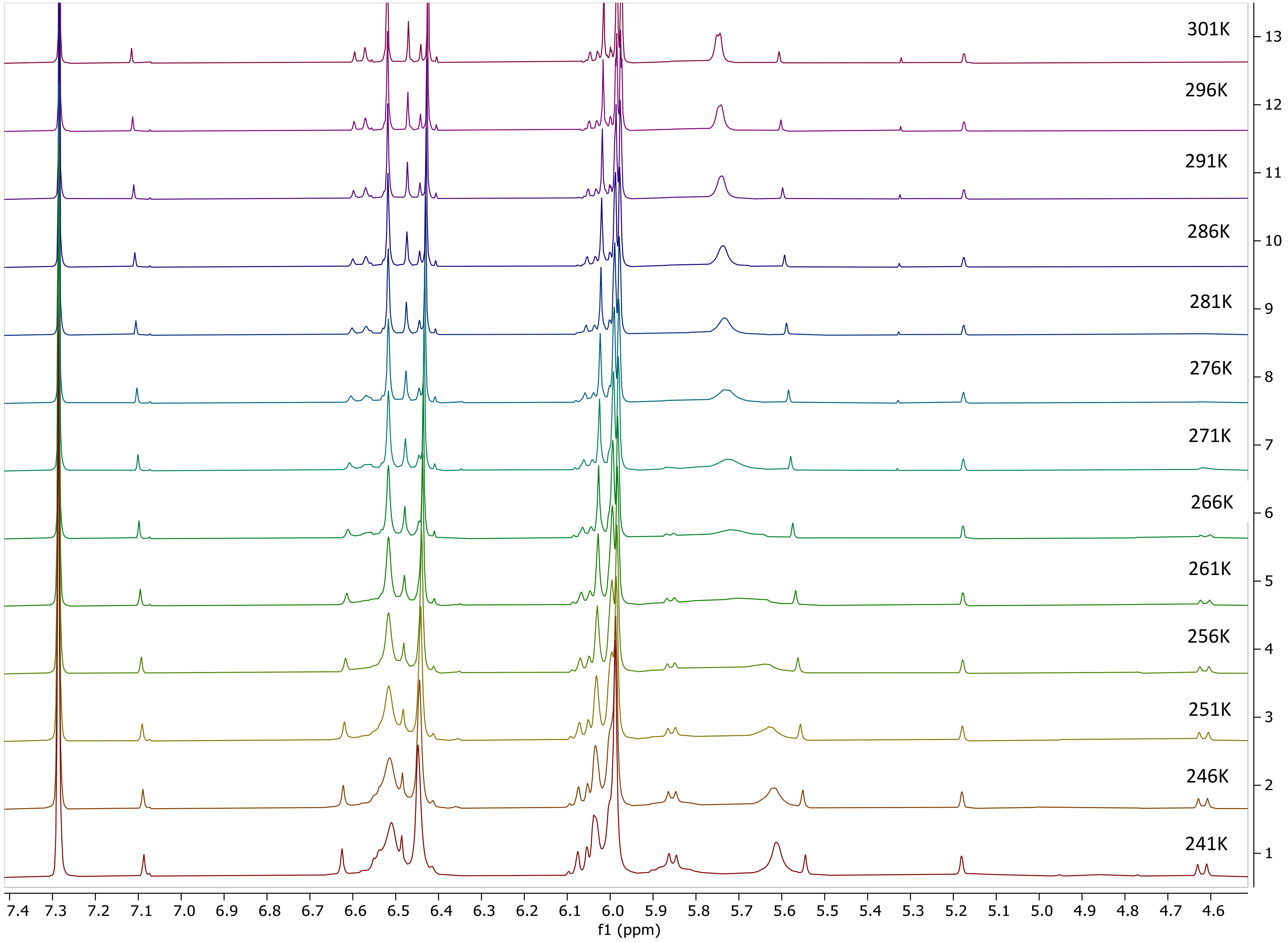

Figure S53. Downfield zoomed ^{1}H VT-NMR data for **2** in $CDCl_3$ (241-301K) (500 MHz).

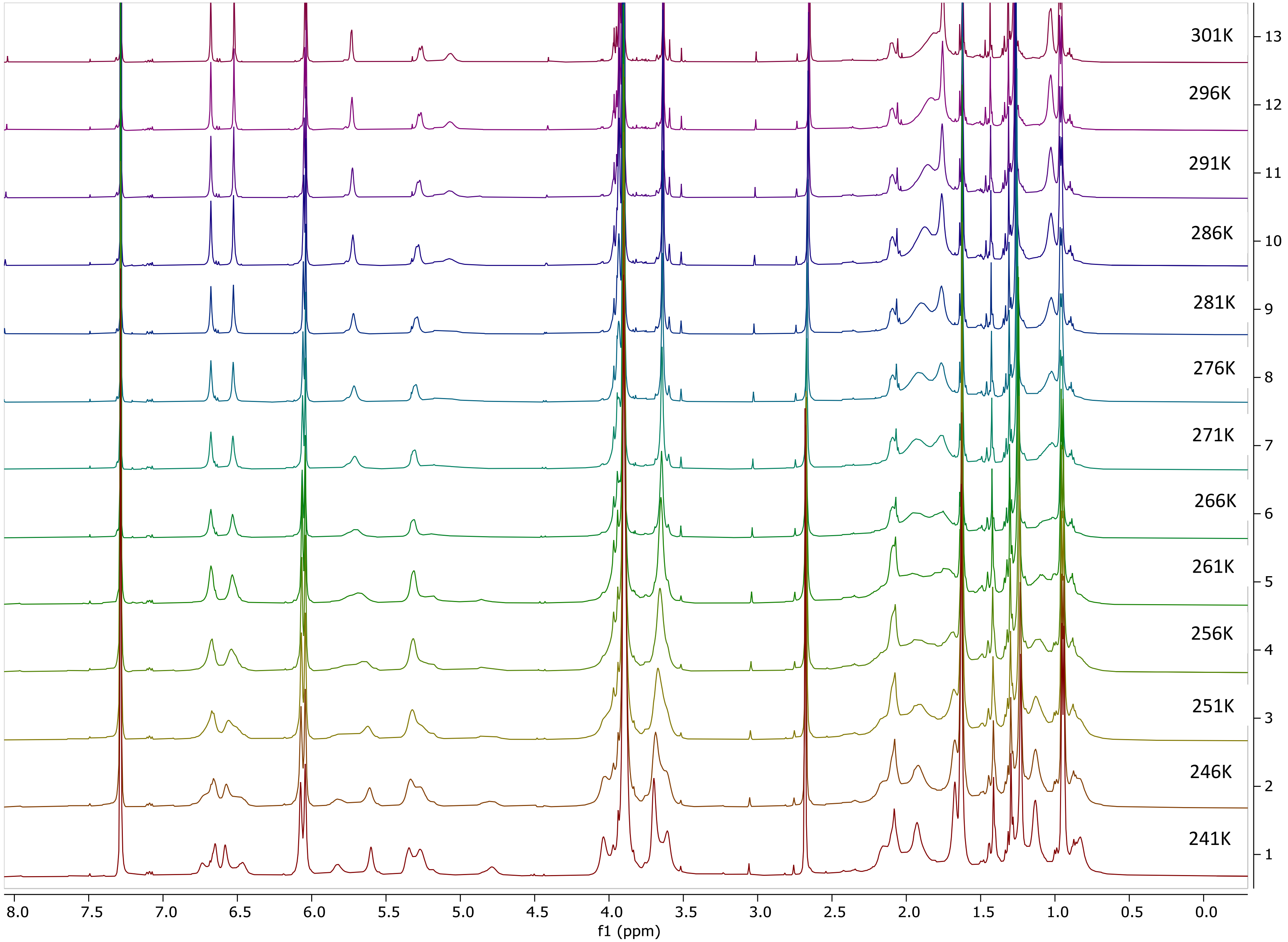

Figure S54. ^{1}H VT-NMR data for **3** in $CDCl_3$ (241-301K) (500 MHz).

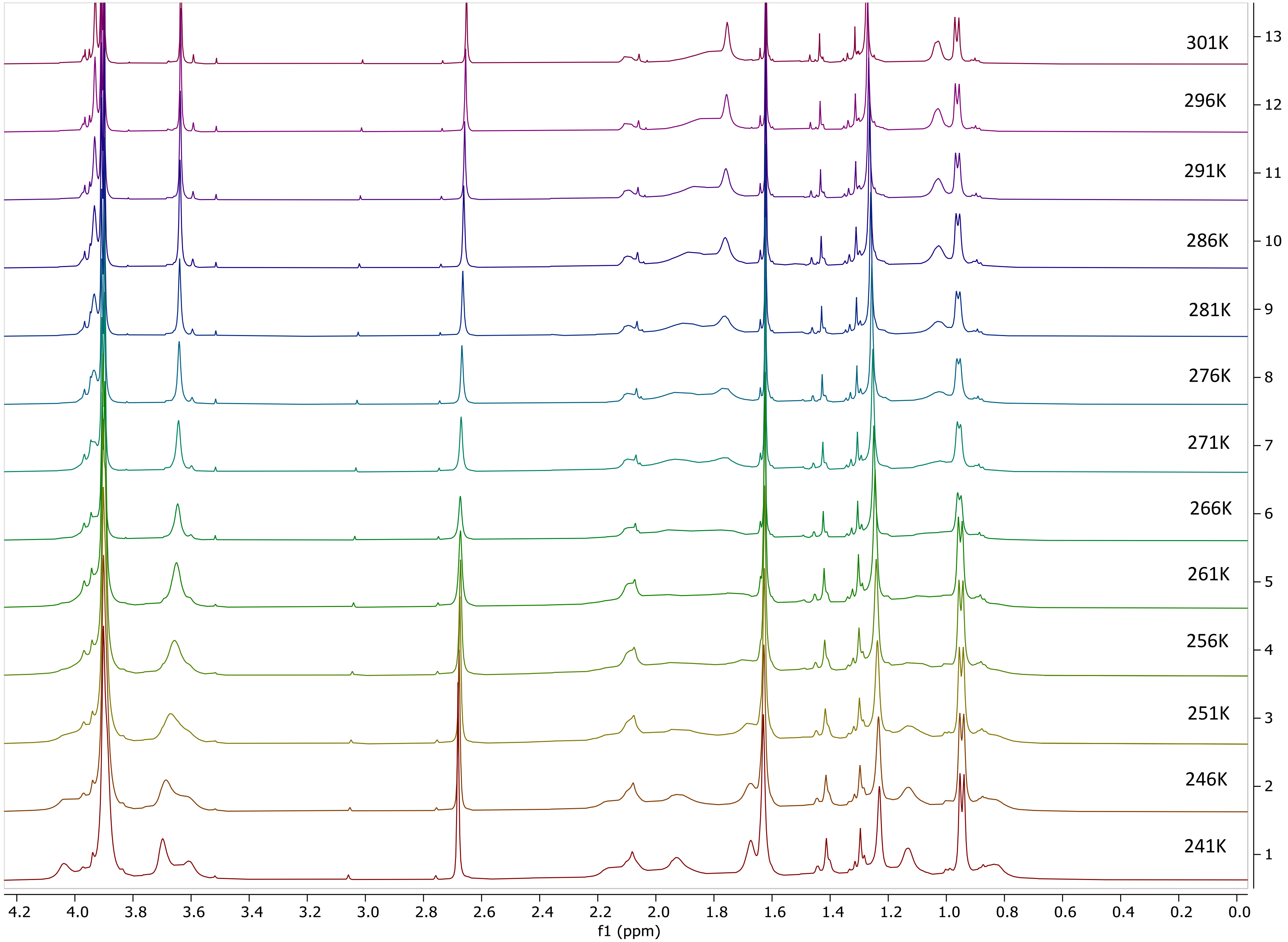

Figure S55. Upfield zoomed ^{1}H VT-NMR data for **3** in $CDCl_3$ (241-301K) (500 MHz).

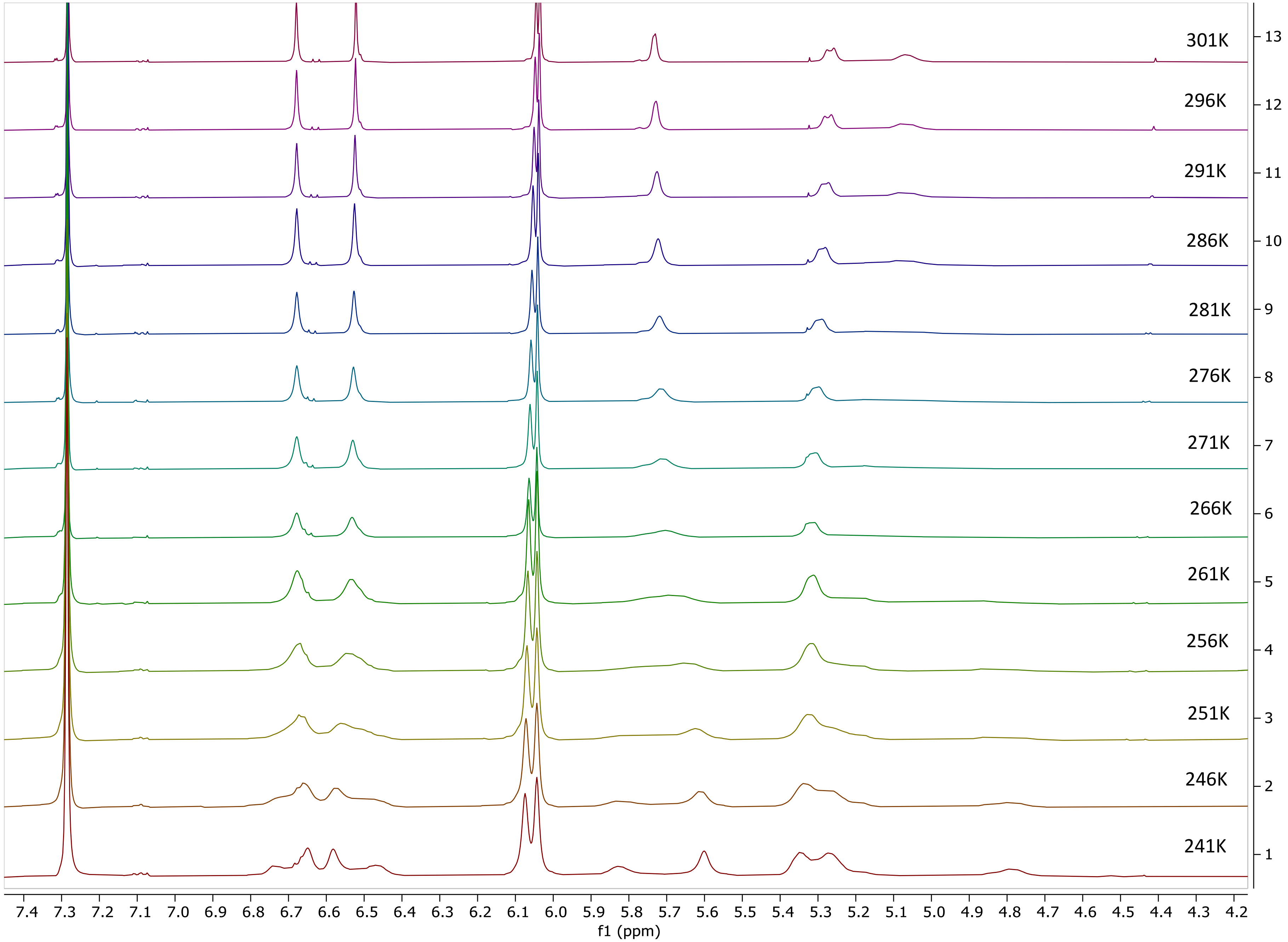

Figure S56. Downfield zoomed ^{1}H VT-NMR data for **3** in $CDCl_3$ (241-301K) (500 MHz).

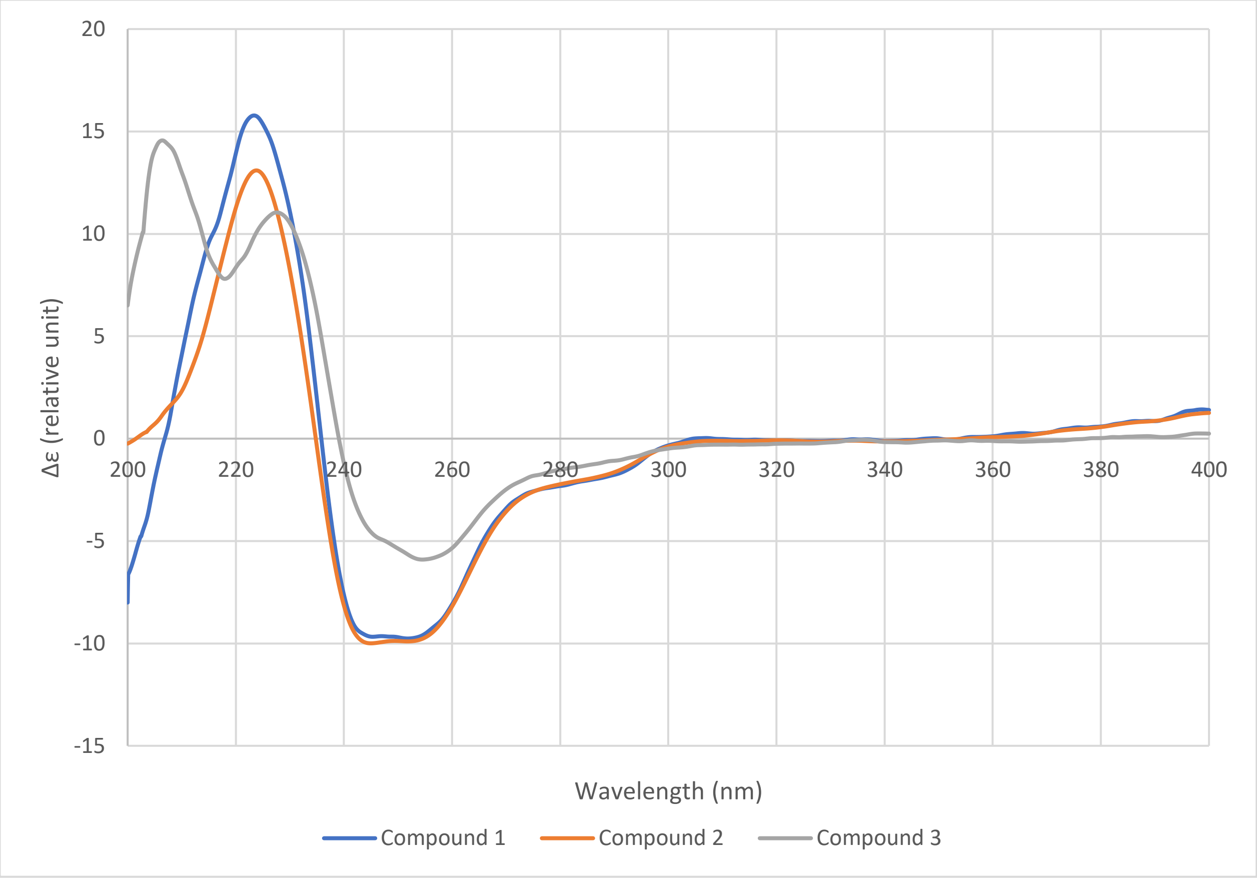

Figure S57. ECD spectra of **1-3**

Section 2a: Calculation results for model compounds

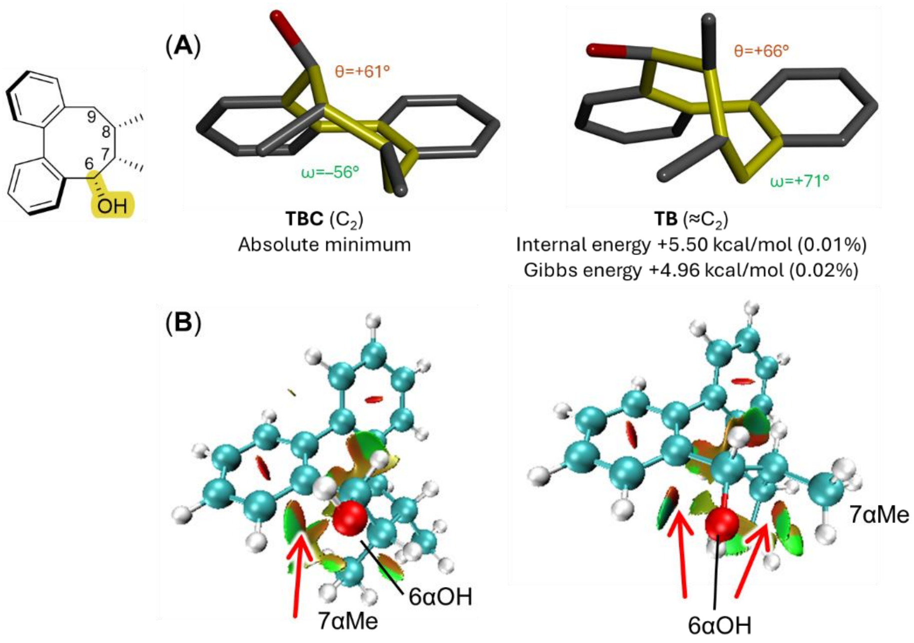

Figure S58. (A) DFT-optimized low-energy structures of (6*S*,7*S*,8*S*)-6-hydroxy-7,8-dimethyldibenzo cyclooctadiene (6αOH). **(B)** NCI analysis showing repulsive (steric) interactions as orange areas indicated by red arrows and Van der Waals interactions as green areas.

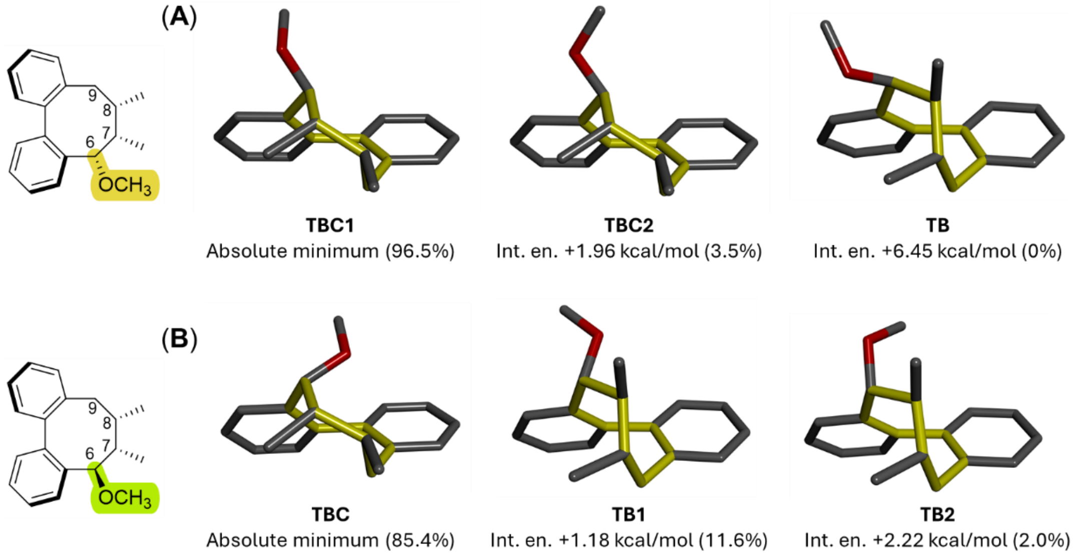

Figure S59. DFT-optimized low-energy structures with relative internal energies of (**A**) (6*S*,7*S*,8*S*)-6-methoxy-7,8-dimethyldibenzo cyclooctadiene (6αOMe) and **(B)** (6*R*,7*S*,8*S*)-6-methoxy-7,8-dimethyl dibenzo cyclooctadiene (6βOMe).

Section 2b: Calculation results for natural products

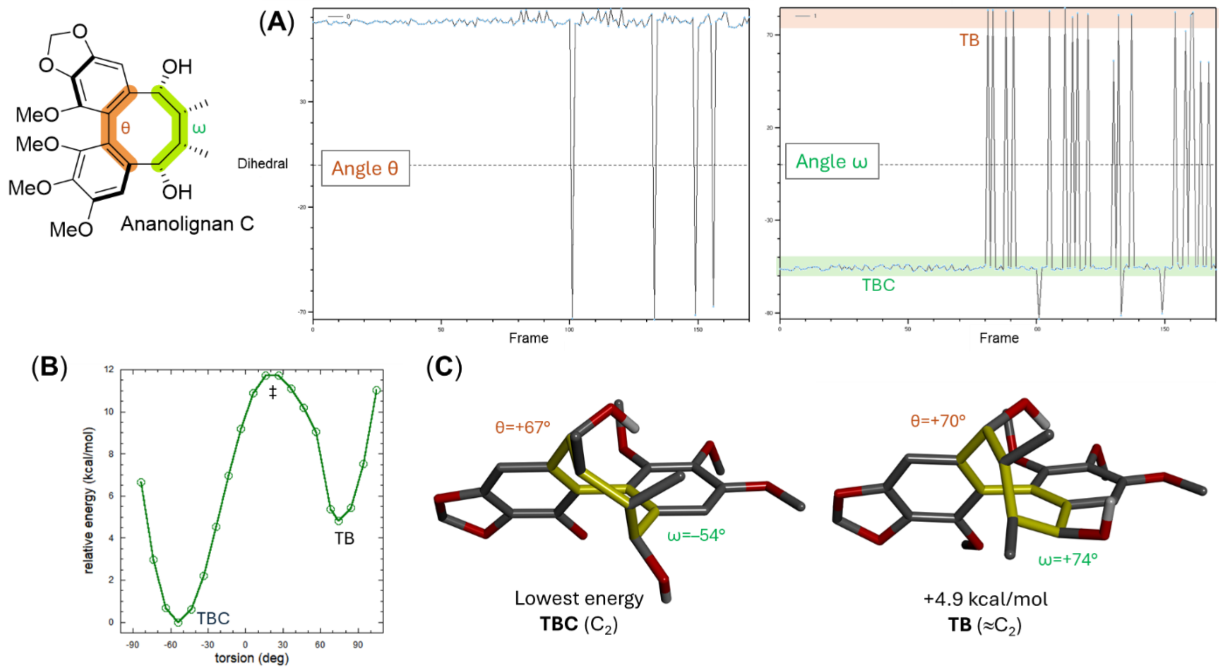

Figure S60. (A) Distribution of angles θ and ω along the CREST dynamics of Ananolignan C, a compound displaying *sharp* ^{13}C-NMR peaks. The coloured stripes highlight the two ranges of values assumed by angle ω for the correct sign of θ (positive). **(B)** Ring torsion energy profiles at 10º steps along C-6/C-7/C-8/C-9 coordinate (relaxed scans at B3LYP-D3BJ/6-31+G(d,p)). Marked minima correspond to structures aside. **(C)-(D)** DFT-optimized structures (B3LYP-D3BJ/6-31+G(d,p)) for the lowest energy conformers, with relative internal energies.

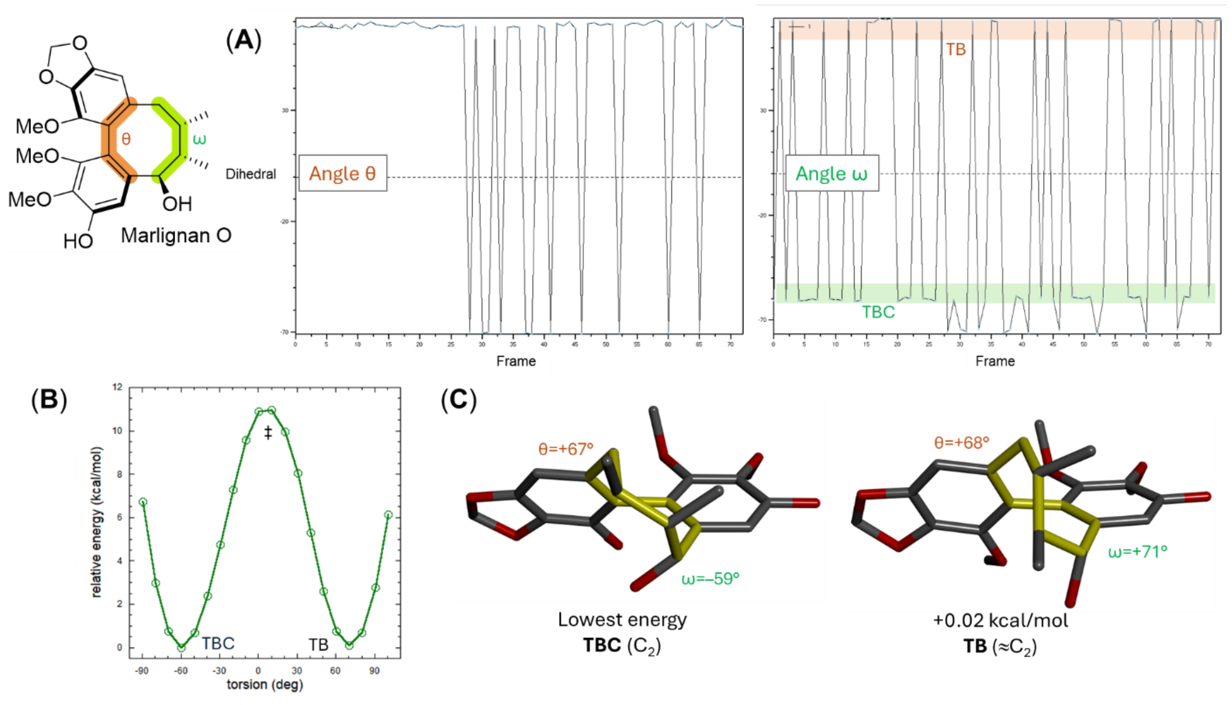

Figure S61. (A) Distribution of angles θ and ω along the CREST dynamics of Marlignan O, a compound displaying *broad* ^{13}C-NMR peaks. The coloured stripes highlight the two ranges of values assumed by angle ω for the correct sign of θ (positive). **(B)** Ring torsion energy profiles at 10º steps along C-6/C-7/C-8/C-9 coordinate (relaxed scans at B3LYP-D3BJ/6-31+G(d,p)). Marked minima correspond to structures aside. **(C)-(D)** DFT-optimized structures (B3LYP-D3BJ/6-31+G(d,p)) for the lowest energy conformers, with relative internal energies.

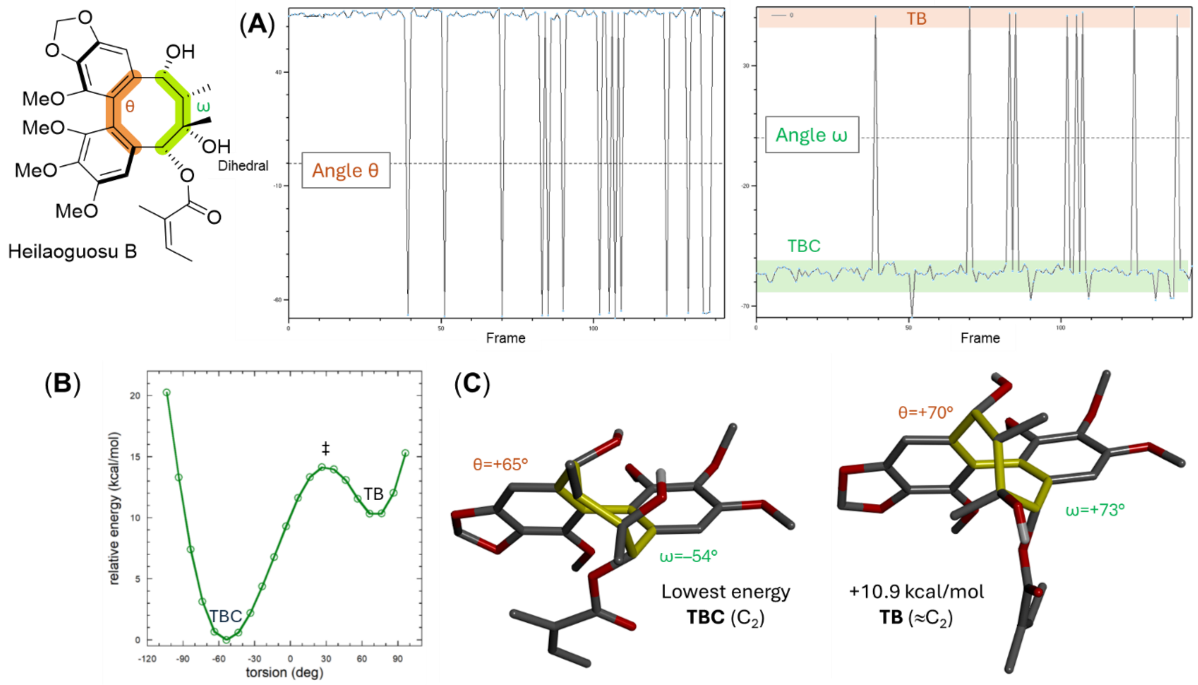

Figure S62. (A) Distribution of angles θ and ω along the CREST dynamics of Heilaohuguosu B, a compound displaying *sharp* ^{13}C-NMR peaks. The coloured stripes highlight the two ranges of values assumed by angle ω for the correct sign of θ (positive). **(B)** Ring torsion energy profiles at 10° steps along C-6/C-7/C-8/C-9 coordinate (relaxed scans at B3LYP-D3BJ/6-31+G(d,p)). Marked minima correspond to structures aside. **(C)-(D)** DFT-optimized structures (B3LYP-D3BJ/6-31+G(d,p)) for the lowest energy conformers, with relative internal energies.

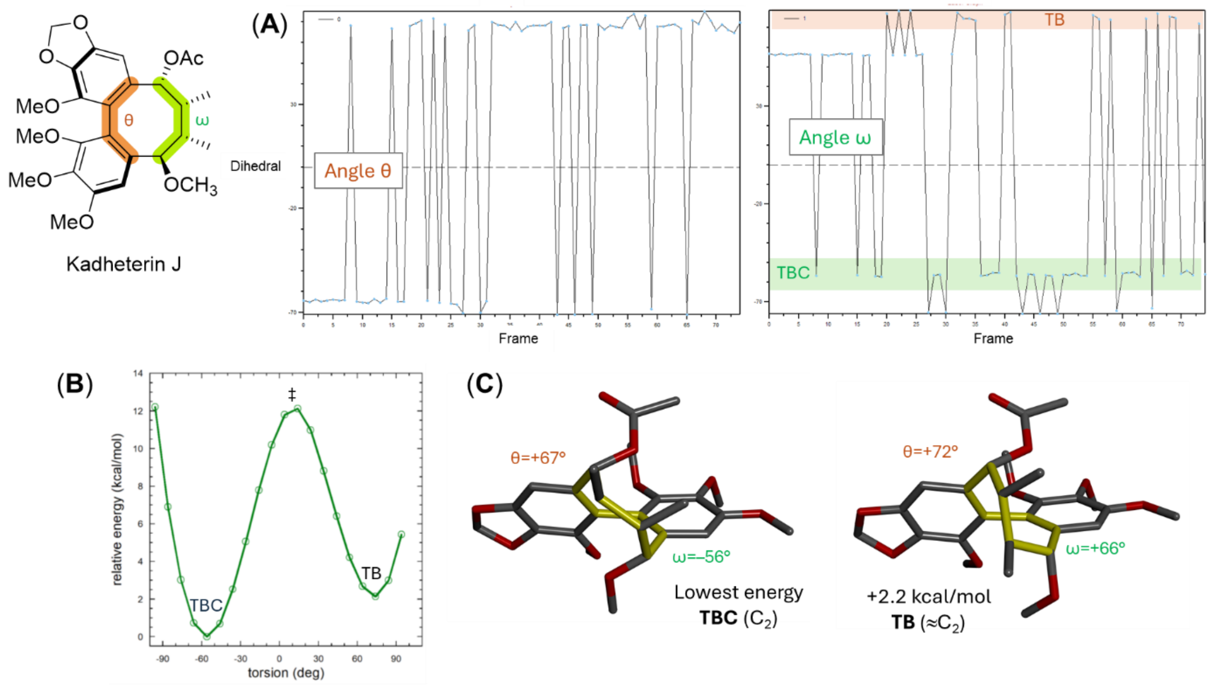

Figure S63. (A) Distribution of angles θ and ω along the CREST dynamics of Kadheterin J, a compound displaying *broad* ^{13}C-NMR peaks. The coloured stripes highlight the two ranges of values assumed by angle ω for the correct sign of θ (positive). **(B)** Ring torsion energy profiles at 10° steps along C-6/C-7/C-8/C-9 coordinate (relaxed scans at B3LYP-D3BJ/6-31+G(d,p)). Marked minima correspond to structures aside. **(C)-(D)** DFT-optimized structures (B3LYP-D3BJ/6-31+G(d,p)) for the lowest energy conformers, with relative internal energies.

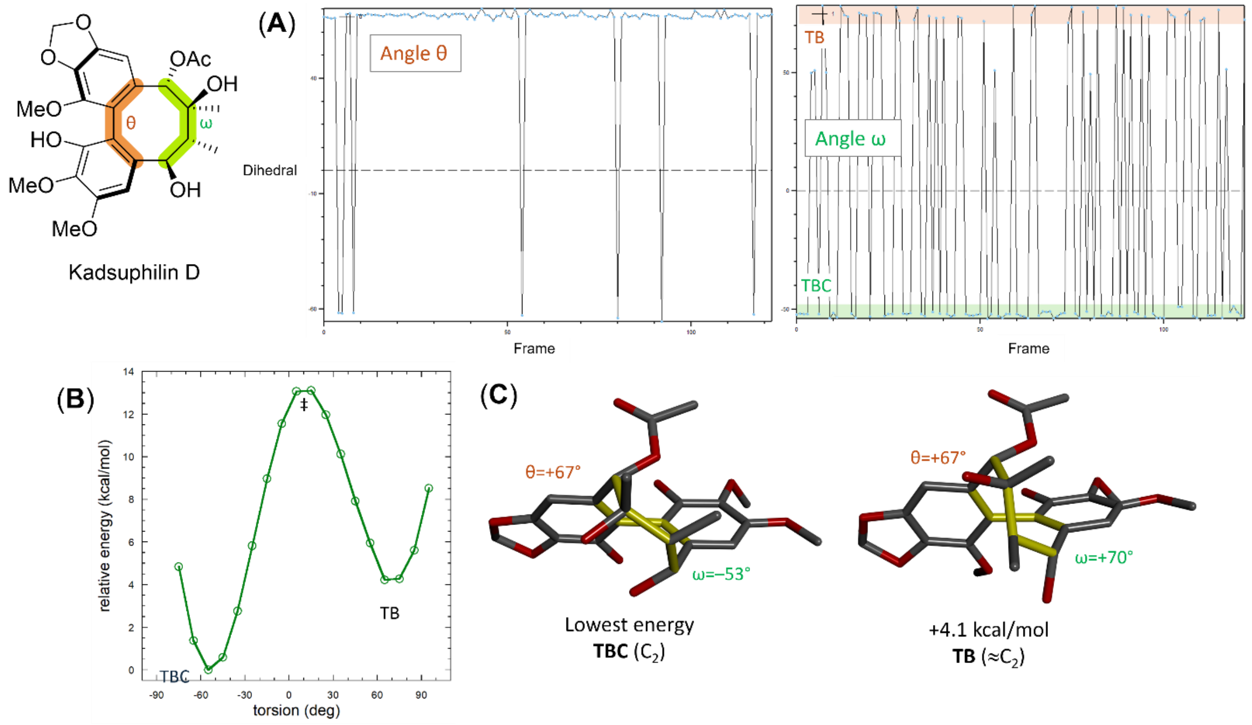

Figure S64. (A) Distribution of angles θ and ω along the CREST dynamics of kadsuphilin D, a compound *for which the* ^{13}*C-NMR spectra could not be obtained*. The coloured stripes highlight the two ranges of values assumed by angle ω for the correct sign of θ (positive). **(B)** Ring torsion energy profiles at 10º steps along C-6/C-7/C-8/C-9 coordinate (relaxed scans at B3LYP-D3BJ/6-31+G(d,p)). Marked minima correspond to structures aside. **(C)-(D)** DFT-optimized structures (B3LYP-D3BJ/6-31+G(d,p)) for the lowest energy conformers, with relative internal energies.

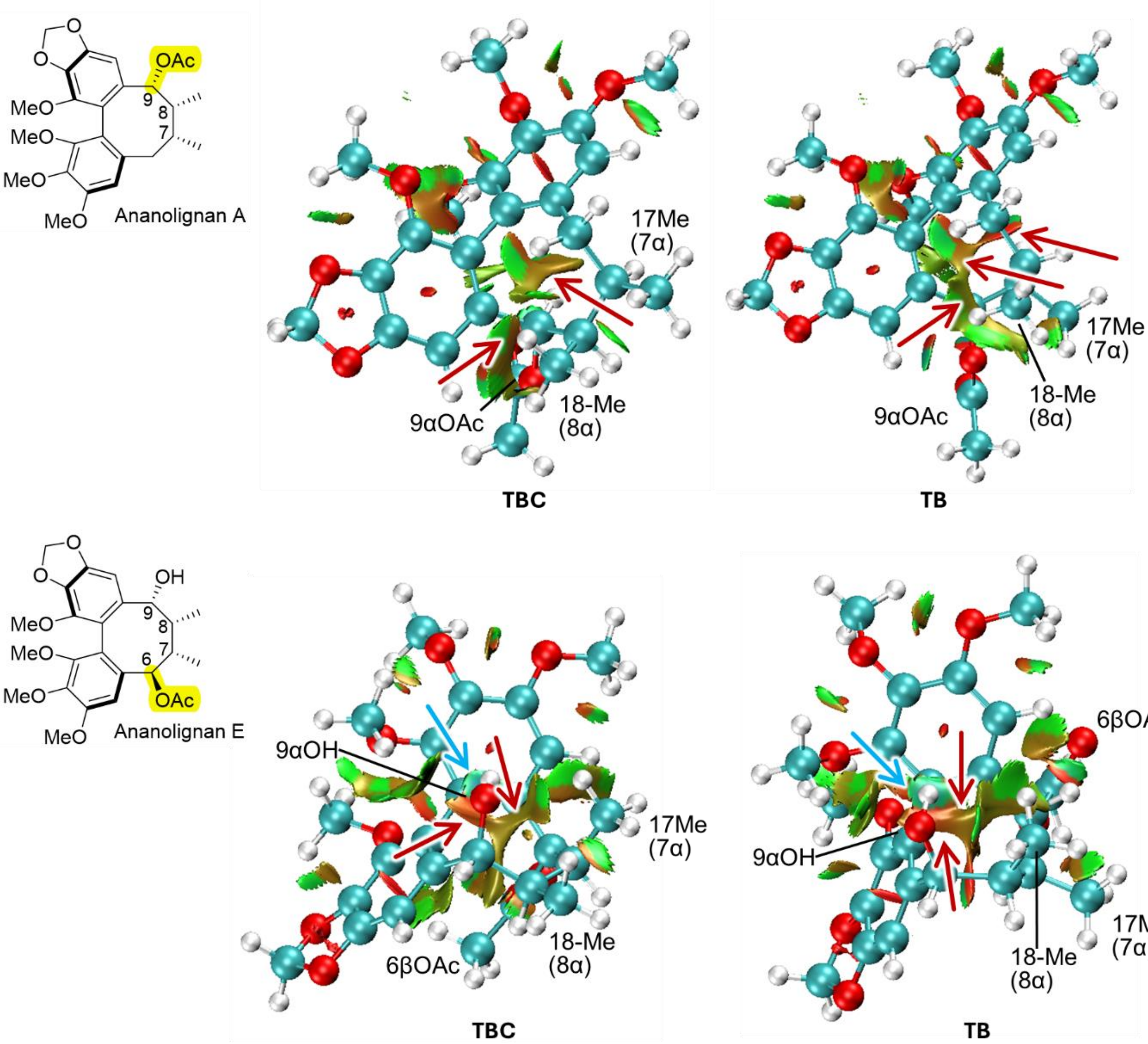

Figure S65. Non-covalent interactions (NCI) analysis for ananolignan A (top) and E (bottom), showing repulsive (steric) interactions as orange areas indicated by red arrows, Van der Waals interactions as green areas, and attractive OH-π interactions as cyan areas indicated by cyan arrows.

Section 3: Anti-inflammatory activity testing

Table S3. Information for cell reporter assays.

Cell assay	Stimulation	Inhibitor
**SW982-NF-κB**	TNF-α (10 ng/mL) + IL-1β (10 ng/mL)	Bay11 (2 µM)
**Jur-NF-κB**	α-CD3 mAb (1.25 µg/mL) + α-CD28 mAb (1.25 µg/mL)	AP-1/NF-κB dual inhibitor (200 nM)
**Jur-NFAT**	Ionomycin (5 µM) **OR** Ionomycin (0.5 µM) + PMA (16 nM)	Cyclosporin A (0.1 µM)
**HEK293-STAT3**	LIF (0.5 ng/mL)	STAT3-inhibitor III (5 µM)
**U937-STAT5**	human GM-CSF (0.4 ng/mL)	STAT5 inhibitor (200 µM) **OR** IQDMA STAT5 inhibitor (5 µM)

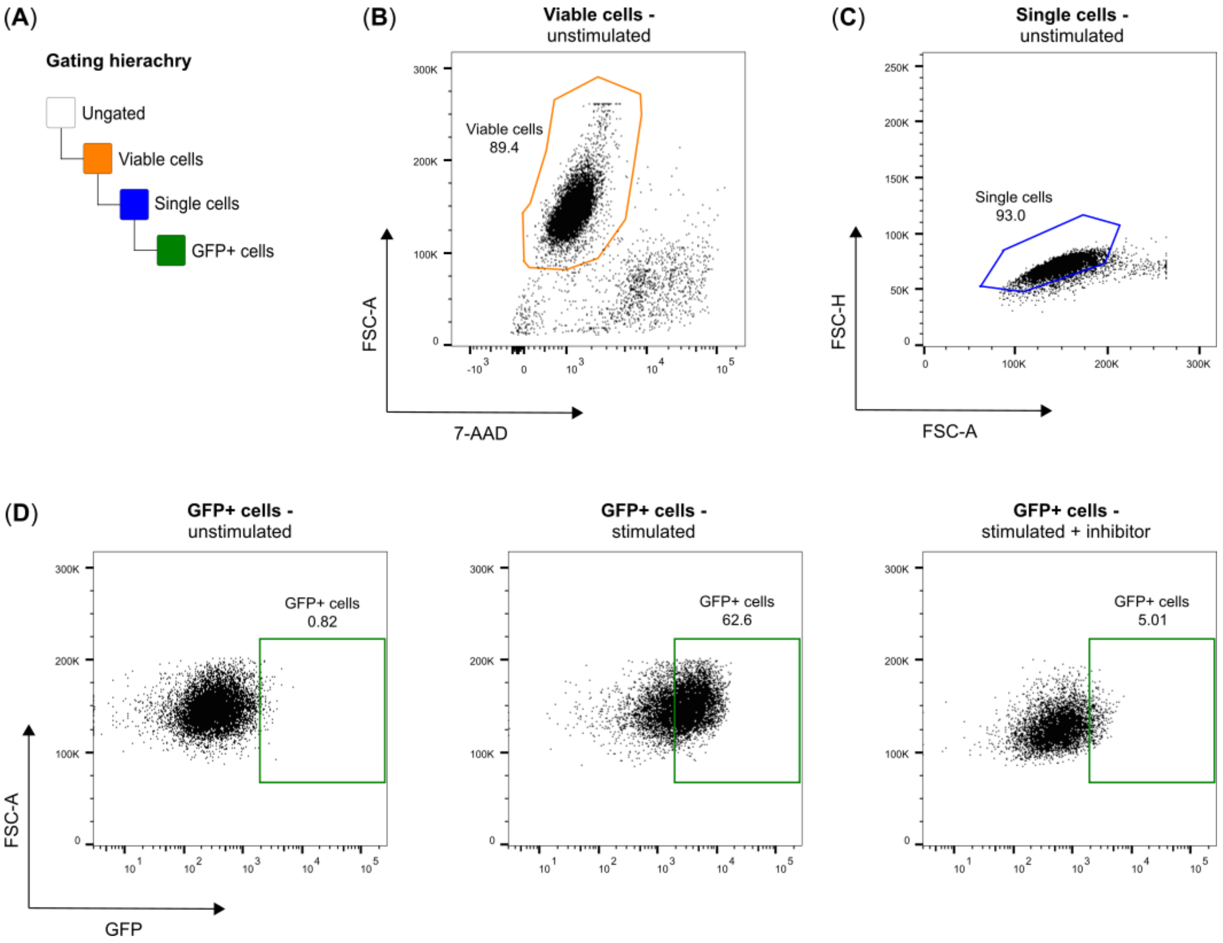

Figure S66. General gating strategy for cell reporter assays. **(A)** Overview over gating hierarchy. **(B)** Gating on viable cells which are 7-AAD negative. **(C)** Doublet discrimination within viable cells. Cells that are too close together to be recognized as single cells appear bigger and are excluded. **(D)** Gating on GFP+ cells in different control conditions within single cells. Unstimulated cells are used to place the gate appropriately. Stimulated cells are used to normalize data and stimulated + inhibitor is used as a control for the assay. Displayed are plots from a representative experiment with U937-STAT5 experiment. Percentages of viable cells, singlets and GFP+ cells vary between cell lines.

Section 4: References

(1) Chang, J.; Reiner, J.; Xie, J. Progress on the chemistry of dibenzocyclooctadiene lignans; *Chem. Rev.* **2005**, *105*, 4581-4609.
